# Multi-omics discovery of exome-derived neoantigens in hepatocellular carcinoma

**DOI:** 10.1186/s13073-019-0636-8

**Published:** 2019-04-30

**Authors:** Markus W. Löffler, Christopher Mohr, Leon Bichmann, Lena Katharina Freudenmann, Mathias Walzer, Christopher M. Schroeder, Nico Trautwein, Franz J. Hilke, Raphael S. Zinser, Lena Mühlenbruch, Daniel J. Kowalewski, Heiko Schuster, Marc Sturm, Jakob Matthes, Olaf Riess, Stefan Czemmel, Sven Nahnsen, Ingmar Königsrainer, Karolin Thiel, Silvio Nadalin, Stefan Beckert, Hans Bösmüller, Falko Fend, Ana Velic, Boris Maček, Sebastian P. Haen, Luigi Buonaguro, Oliver Kohlbacher, Stefan Stevanović, Alfred Königsrainer, HEPAVAC Consortium, Hans-Georg Rammensee

**Affiliations:** 1 Department of General, Visceral and Transplant Surgery, University Hospital Tübingen, Hoppe-Seyler-Str. 3, D-72076 Tübingen, Germany; 2 Interfaculty Institute for Cell Biology, Department of Immunology, University of Tübingen, Auf der Morgenstelle 15, D-72076 Tübingen, Germany; 3 German Cancer Consortium (DKTK) and German Cancer Research Center (DKFZ) Partner Site Tübingen, Tübingen, Germany; 4 Department of Clinical Pharmacology, University Hospital Tübingen, Auf der Morgenstelle 8, D-72076 Tübingen, Germany; 5 Institute for Translational Bioinformatics, University Hospital Tübingen, Tübingen, Germany; 6 Quantitative Biology Center (QBiC), University of Tübingen, Auf der Morgenstelle 10, D-72076 Tübingen, Germany; 7 Center for Bioinformatics, University of Tübingen, Sand 14, D-72076 Tübingen, Germany; 8 Department of Computer Science, Applied Bioinformatics, Sand 14, D-72076 Tübingen, Germany; 9 Present address: European Molecular Biology Laboratory, European Bioinformatics Institute (EMBL-EBI), Wellcome Trust Genome Campus, Hinxton, Cambridgeshire, CB10 1SD, United Kingdom; 10 Institute of Medical Genetics and Applied Genomics, University Hospital Tübingen, Calwerstr. 7, D-72076 Tübingen, Germany; 11 Present address: Immatics Biotechnologies GmbH, Paul-Ehrlich-Str. 15, D-72076 Tübingen, Germany; 12 NGS Competence Center Tübingen (NCCT), University of Tübingen, Tübingen, Germany; 13 Present address: Department of General and Visceral Surgery, Schwarzwald-Baar Hospital, Klinikstr. 11, D-78052 Villingen-Schwenningen, Germany; 14 Institute of Pathology and Neuropathology, University Hospital Tübingen, Liebermeisterstr. 8, D-72076 Tübingen, Germany; 15 Interfaculty Institute for Cell Biology, Proteome Center Tübingen (PCT), University of Tübingen, Auf der Morgenstelle 15, 72076 Tübingen, Germany; 16 Internal Medicine, Department for Oncology, Hematology, Immunology, Rheumatology and Pulmonology, University of Tübingen, Otfried-Müller-Str. 10, D-72076 Tübingen, Germany; 17 Cancer Immunoregulation Unit, Istituto Nazionale per lo Studio e la Cura dei Tumori, “Fondazione Pascale” – IRCCS, 80131 Naples, Italy; 18 Max Planck Institute for Developmental Biology, Biomolecular Interactions, Spemannstr. 35, D-72076 Tübingen, Germany

**Keywords:** Hepatocellular carcinoma, HLA, HLA ligandomics, Immunoinformatics, Immunotherapy, Liver cancer, Mass spectrometry, Multi-omics, Neoantigen, Next-generation sequencing, Peptide prediction, Personalized medicine

## Abstract

**Background:**

Although mutated HLA ligands are considered ideal cancer-specific immunotherapy targets, evidence for their presentation is lacking in hepatocellular carcinomas (HCCs). Employing a unique multi-omics approach comprising a neoepitope identification pipeline, we assessed exome-derived mutations naturally presented as HLA class I ligands in HCCs.

**Methods:**

In-depth multi-omics analyses included whole exome and transcriptome sequencing to define individual patient-specific search spaces of neoepitope candidates. Evidence for the natural presentation of mutated HLA ligands was investigated through an in silico pipeline integrating proteome and HLA ligandome profiling data.

**Results:**

The approach was successfully validated in a state-of-the-art dataset from malignant melanoma, and despite multi-omics evidence for somatic mutations, mutated naturally presented HLA ligands remained elusive in HCCs. An analysis of extensive cancer datasets confirmed fundamental differences of tumor mutational burden in HCC and malignant melanoma, challenging the notion that exome-derived mutations contribute relevantly to the expectable neoepitope pool in malignancies with only few mutations.

**Conclusions:**

This study suggests that exome-derived mutated HLA ligands appear to be rarely presented in HCCs, *inter alia* resulting from a low mutational burden as compared to other malignancies such as malignant melanoma. Our results therefore demand widening the target scope for personalized immunotherapy beyond this limited range of mutated neoepitopes, particularly for malignancies with similar or lower mutational burden.

**Electronic supplementary material:**

The online version of this article (10.1186/s13073-019-0636-8) contains supplementary material, which is available to users.

## Background

Hepatocellular carcinoma (HCC) is among the malignancies with the highest death toll on a global scale [1] and with very limited therapeutic options. Particularly in advanced stage, long-term survival is uncommon [2]. Although it has been shown that the microenvironment of the liver is tolerogenic and impairs immune responses [3], antigen-specific T cell responses do occur [4]. Since infiltration of HCCs with T cells [5] and spontaneous immune responses correlate with longer survival [6] but mostly prove weak and insufficient on their own, immunotherapies unleashing the immune system hold great promise.

Immune checkpoint (ICP) inhibitors demonstrating the potency and effectiveness of the immune system to fight malignancy [7] have set the stage for cancer immunotherapies. In contrast to established cytostatic treatments for cancer, this new class of drugs has enabled long-term survival in advanced and metastatic disease previously considered incurable [8]. However, although in some malignancies ICP inhibitors have proven highly effective, results for other cancers remain disappointing. One probable mode of action for ICP inhibitors is the induction and/or restoration of T cell effector functions against individual somatic tumor mutations presented by HLA molecules (i.e., mutated neoepitopes) [9]. Since these mutated HLA ligands were unacquainted to the immune system before carcinogenesis, they have been proposed as ideal tumor-specific targets [10, 11].

In malignant melanoma (Mel), where ICP inhibitors were established first, mutational load was shown to strongly correlate with survival [12]. This has been corroborated in lung cancer [13] and colorectal carcinoma, where in the latter impressive survival benefits remained strictly limited to mismatch repair-deficient carcinomas featuring very high numbers of mutations [14]. As elevated somatic mutation rates raise the odds for generating neoantigens, this supports the notion they may be critical for ICP inhibitor effectiveness [15]. Another line of evidence suggests that neoantigens recognized by T cells can generate impressive clinical effects, when identified and exploited for therapeutic purposes. This has been shown in remarkable case reports *inter alia* in advanced Mel [16] and metastatic cholangiocarcinoma [17].

With current affordable next-generation sequencing (NGS) and bioinformatics, an array of approaches predicting HLA-restricted neoantigens from virtually any tumor has emerged [18–20]. Indeed, at present most attempts are restricted to in silico analyses, lacking actual proof that the predicted neoantigens are relevant or even exist. So far, tangible evidence is scarce and mainly restricted to T cell recognition [21]. Therefore, one frequently missing link is proof of neoantigen presentation on native tumor tissue. Such an endeavor is very challenging and has been shown feasible in mouse models [22] and cell lines [23] but in human solid tumors hitherto merely in Mel at low numbers using mass spectrometry (MS), defining the current state-of-the-art [24, 25].

Since both individual cancer traits and mutational load vary strongly between different tumor entities [26, 27], these properties may ultimately restrict the foreseeable success and feasibility of neoantigen-targeted precision cancer medicine. In HCCs, only a small proportion of about 10% of patients showed mutations potentially accessible for drug therapy [28], whereas preliminary data for ICP inhibitors showed objective response rates in 15–20% of patients combined with a manageable safety profile [29], making neoantigens in principle an interesting case for precision cancer medicine and the use of NGS.

Hence, we performed unprecedented in-depth multi-omics analyses encompassing whole exome and transcriptome sequencing, combined with proteome and HLA ligandome profiling in selected HCC patients aiming to obtain evidence for the natural presentation of exome-derived mutated HLA ligands, employing various strategies.

## Materials and methods

### Clinical specimens

Clinical specimens from patients (*n* = 16; median age: 74 years; min.–max. 55–85 years; 75% men) undergoing liver resection for hepatocellular carcinomas (HCCs), encompassing both non-malignant and malignant liver tissue as well as peripheral blood, were obtained directly after surgery and cryopreserved (for patients’ tumor characteristics, see Additional file 1: Table S1). HCC diagnosis and predominant tumor fraction within samples were histologically confirmed by an expert pathologist. All included patients were negative for chronic viral hepatitis (hepatitis B and C) and without systemic pretreatment for their malignancy.

### Next-generation sequencing

DNA and RNA were extracted from fresh frozen tissue and PBMCs, respectively (a sample and analysis overview is provided in Additional file 1: Table S2). After sample preparation and enrichment, paired-end whole exome sequencing (WES) and whole transcriptome sequencing were performed on an Illumina system (details are provided in Additional file 2).

### HLA typing

Typing at four-digit resolution using WES data was performed by OptiType [30] for HLA class I alleles (see Additional file 1: Table S3) as previously described [31] and confirmed in selected cases by molecular HLA typing (using clinically validated LUMINEX and sequence-based typing) during clinical routines.

### Isolation of naturally presented HLA ligands from tissues for HLA ligandomics

HLA class I-peptide complexes were isolated from HCC and corresponding (non-malignant) liver tissue samples by immunoaffinity purification using the pan-HLA class I-specific monoclonal antibody W6/32 [32] (produced in-house at the Department of Immunology, Tübingen, Germany) and eluted using 0.2% trifluoroacetic acid as described previously [33].

### Analysis of HLA ligands by liquid chromatography-coupled tandem mass spectrometry (LC-MS/MS)

HLA class I ligand extracts were measured once or in multiple technical replicates, as described previously [33, 34]. Samples were separated by UHPLC and eluting peptides were analyzed using collision-induced dissociation (CID) in an online coupled Orbitrap mass spectrometer. In addition to data-dependent acquisition (DDA), selected ion monitoring (SIM) and parallel reaction monitoring (PRM) targeted tandem MS (tMS2) was performed for selected samples to enhance the sensitivity and robustness of neoantigenic peptide identification (details are provided in Additional file 2).

### HLA ligandomics data analysis

MS data obtained from HLA immunoprecipitates was analyzed using tools of the open-source software library for LC/MS OpenMS (2.3) [35]. Identification and post-scoring were performed using the OpenMS adapters to Comet 2016.01 rev. 3 [36] and Percolator 3.1.1 [37] at a peptide spectrum match (PSM) false discovery rate (FDR) threshold of 5%. Database search was performed against a personalized version of the human reference proteome (Swiss-Prot, reviewed UP000005640), including the patient-specific mutanome without enzymatic restriction and methionine oxidation as the only variable modification.

### Database matching

HLA ligandome database queries refer to the in-house database (maintained at the Department of Immunology) encompassing > 300,000 unique HLA class I-eluted peptides identified through LC-MS/MS in diverse tissues (non-malignant samples as well as samples with pathologies including malignancies). Database matching was carried out using rSQL, querying for an exact string match of a wild-type ligand (WT^lig^) corresponding to the respective predicted mutated neoepitope (PNE). All HLA class I allotypes of the HCC and Mel cohort were covered by respective samples in the database. Each sample containing the WT^lig^ was counted as a separate match (further details are provided in Additional file 2). Besides neoepitopes, we additionally screened our HCC HLA class I ligandome dataset against cancer-testis antigens (CTAs) as deposited in the CTDatabase (http://www.cta.lncc.br; [38]).

### Protein in-gel digestion for shotgun protein identification

Sample lysates were separated by SDS-PAGE. Coomassie-stained gel pieces were digested using trypsin. Peptides were desalted using C18 Stage tips and subjected to LC-MS/MS analysis.

### Shotgun protein tandem mass spectrometry

Liquid chromatography-coupled tandem mass spectrometry (LC-MS/MS) analyses were performed on an EasyLC nano-HPLC system (Proxeon Biosystems, Roskilde, Denmark) coupled to an LTQ Orbitrap Elite mass spectrometer (ThermoFisher) (additional details are provided in Additional file 2).

### Proteomic data analysis

MS data were processed with MaxQuant software suite v.1.5.2.8 [39]. Database search was performed using the Andromeda search engine [40], integrated into the MaxQuant framework. The human reference database was obtained from UniProt (taxonomy ID 9606, containing 91,646 protein entries and 285 commonly occurring laboratory contaminants) and concatenated with the patient-specific mutanome. Endoprotease trypsin was fixed as enzyme with a maximum of two missed cleavages. Oxidation of methionines and *N*-terminal acetylation were specified as variable modifications, whereas carbamidomethylation of cysteines was defined as a fixed modification. Initial maximum allowed mass tolerance was set to 6 ppm. Re-quantify was enabled. A FDR of 1% was applied at peptide and protein level.

### Bioinformatics

Data management and bioinformatic analysis was performed through the qPortal instance at the Quantitative Biology Center (QBiC), Tübingen, if not stated otherwise [41].

### Variant calling

Reads were processed using the megSAP pipeline (https://github.com/imgag/megSAP) and the ngs-bits package (https://github.com/imgag/ngs-bits) by the Department of Medical Genetics and Applied Genomics (Tübingen, Germany). Reads were mapped against the Genome Reference Consortium Human Build 37 (GRCh37) using BWA-mem [42]. Somatic variant calling was performed using Strelka and Strelka2 [43, 44] or with a proprietary software (CeGaT GmbH, Tübingen, Germany). Somatic mutations were annotated using SnpEff 4.1 k [45]. Further details are provided in Additional file 2.

### Gene expression analysis

Gene expression values were calculated as fragments per kilobase of exon per million reads mapped (FPKM) of the corresponding transcripts and RNA tumor sequencing depth at the corresponding variant position. Mapping of RNA reads was done using TopHat 2 (v2.0.12) [46]. Details are provided in Additional file 2.

### Protein quantification analysis of shotgun proteomics data

Label-free protein quantification was done using MaxQuant v1.5.00 [39]. Parameter groups were defined for non-malignant tissue- and tumor-derived raw files, respectively. The multiplicity was set to one. Protein *N*-terminal acetylation as well as oxidation of methionine residues were selected as variable modifications, whereas carbamidomethylation of cysteine residues was set as fixed modification. Trypsin was selected as protease with a specific digestion mode. Further, we specified the match type as *MatchFromAndTo* and set the number of *MaxMissedCleavages* to two. Requantification and matching between runs were enabled. As a reference, we specified the Swiss-Prot reviewed human proteome (*version UP000005640*, derived: 02/16/2016).

### Peptide prediction

To define the sample-specific mutated peptide search space (PSS), peptides of 8–11 amino acid length were constructed by sliding a shifting window of the peptide length over the affected mutated positions. Resulting peptides were filtered against the human proteome (UniProt *UP000005640*, derived: 02/29/16) and the Ensembl proteome reference (release 84, 04/27/2016) to exclude peptides contained within wild-type proteins. Transcript information was retrieved *via* *BioMart*, based on the stable database version of GRCh37 (http://feb2014.archive.ensembl.org). HLA-binding prediction was performed with SYFPEITHI [47], netMHC 4.0 [48, 49], and netMHCpan 3.0 [50, 51]. The workflow was implemented using FRED2 [52] (see Additional file 2 for further details).

### Differential gene expression analysis and pathway analysis

Differential gene expression analysis was performed using the R package DESeq2 [53]. Expression data of HCC datasets from TCGA were retrieved and analyzed with the recount2 package [54].

Pathway analysis was carried out using clusterProfiler [55] and Pathview [56]. Differentially expressed genes were categorized using DAVID [57]. Details are provided in Additional file 2.

## Results

### A multi-omics approach to detect mutated HLA ligands in HCCs

We performed analyses of malignant and non-malignant liver tissue, resected during surgery for HCCs (Additional file 1: Table S1 & Table S2), by a multi-omics approach encompassing analyses on exome (*n* = 16), transcriptome (*n* = 16), shotgun proteome (*n* = 7), and HLA ligandome level (i.e., HLA-presented peptides; *n* = 16). Multi-allelic HLA class I expression was confirmed in all patients of our HCC cohort (results are provided in Additional file 1: Table S3). The overall aim of our research was to identify individual exome-derived somatic tumor mutations resulting in natural HLA ligands presented to T cells.

### Detection of somatic variants (mutations) in HCCs

**Fig. 1:**
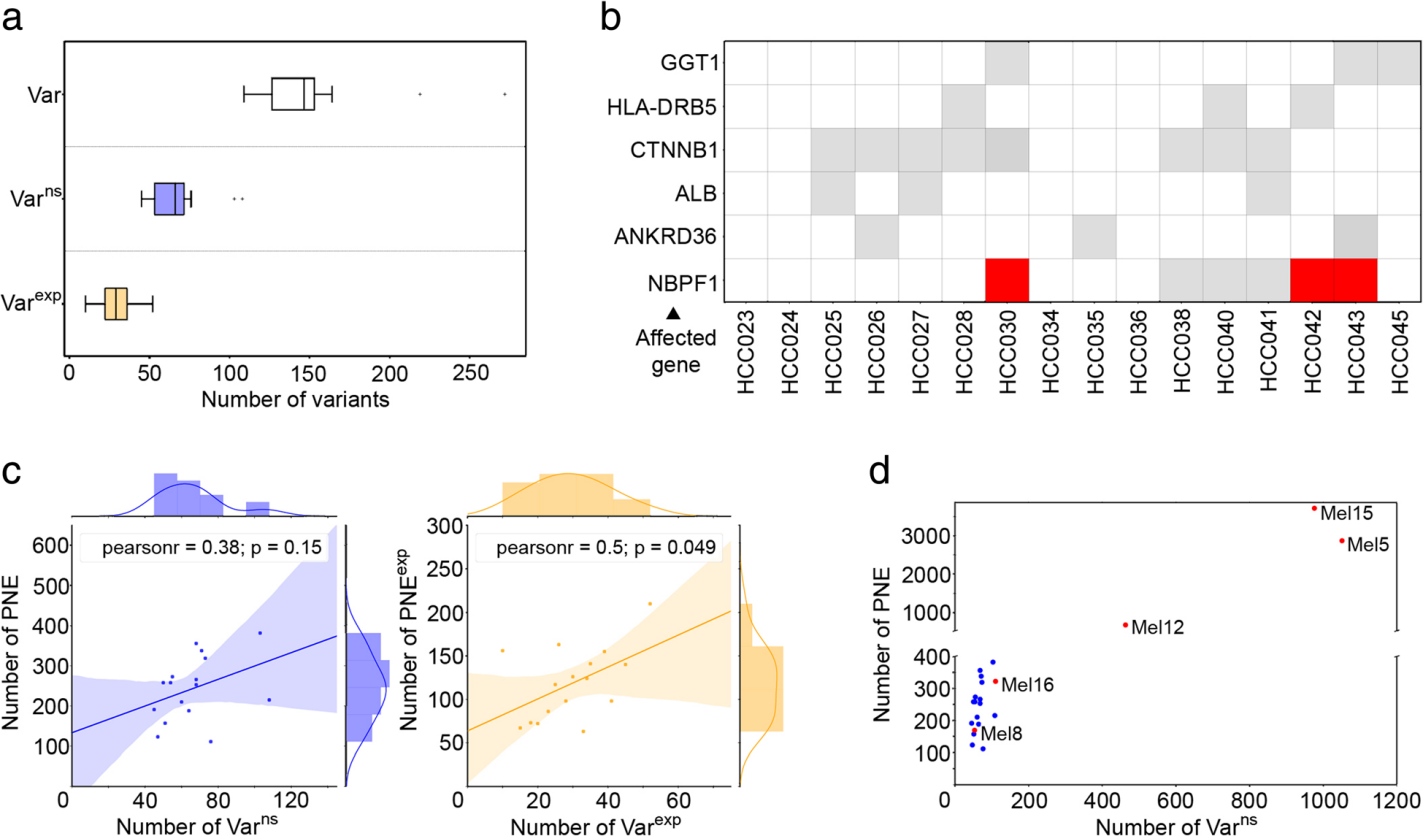
Characterization of somatic variants and their potential for HLA presentation in HCC. **a** Numbers of somatic variants across HCC patients (*n* = 16). Numbers are shown for all variants passing initial filtering **(**Var), coding non-synonymous variants (Var^ns^), and coding non-synonymous variants with RNA level evidence (Var^exp^). Boxplots show means ± SD. **b** Var^exp^ shared among HCC patients. Var^exp^ affecting identical genes in ≥ 3 patients are displayed in gray. Var^exp^ observed at identical genomic positions are displayed in red (*the shown HLA-DR variants should be cautiously interpreted as potential artifacts*). **c** Correlation between Var^ns^ and predicted HLA-binding neoepitopes (PNE) (left; blue). Correlation between Var^exp^ and expressed PNE (PNE^exp^) (right; orange). **d** Scatter plot of numbers of Var^ns^ and PNE in HCC patients (blue) and a benchmarking dataset of melanoma (Mel) patients (red) as previously described by Bassani-Sternberg et al. [24]

On average, we detected 151 ± 40 somatic variants (Var) per HCC, including single nucleotide variants, small insertions/deletions, and frameshift variants; thereof, 44% (66 ± 19) cause changes in the amino acid sequence of the encoded protein (i.e., non-synonymous variants; Var^ns^ - a glossary of abbreviations and terminology used is provided as Table 1), when referenced against DNA from blood. From these Var^ns^, on average about half were also detectable on transcript level (44 ± 10%; Fig. 1a). Across all patients, we observed 1039 unique Var^ns^ in total, affecting 864 different genes and 45% of them (*n* = 392) with additional evidence on RNA level (Var^exp^). This translates to an average tumor mutational burden (TMB; estimated as previously described [58]) of 1.89 ± 0.49 per megabase observed in our HCC cohort (see Additional file 1: Table S4).

**Table 1:** Glossary of relevant abbreviations used

Var	Somatic variant (single nucleotide variant [SNV], insertion/deletion [InDel], frameshift variant)
Var^ns^	Non-synonymous somatic variant (i.e., somatic mutation)
Var^exp^	Expressed non-synonymous somatic variant
PNE	Predicted mutated neoepitope
PNE^exp^	Predicted mutated neoepitope with evidence on transcript level
PNE^prot^	Predicted mutated neoepitope with evidence on proteome level
NE^lig^	Mutated neoantigen with evidence on HLA ligandome level (HLA class I)
WT^lig^	Wild-type peptide corresponding to PNE with evidence on HLA ligandome level (HLA class I)
neoantigen/neoepitope	Mutated HLA-presented peptide (potentially) recognizable by (T cells of) the immune system
TMB	Tumor mutational burden (non-synonymous somatic variants per megabase)

Assessing mutational hotspots, we observed alterations (Var^exp^) in *β-catenin* (*CTNNB1*; 50%) and in *neuroblastoma breakpoint family*, *member 1* (*NBPF1*; 38%), but also in genes encoding proteins typically expressed in the liver, such as *albumin* (*ALB*; 19%), *apolipoprotein b* (*APOB*; 13%), and γ-glutamyltransferase (*GGT1*; 19%) (Fig. 1b). Var^exp^ frequently affected the HLA class II loci *HLA-DRB1* (6%), *HLA-DQA1* (13%), and *HLA-DRB5* (19%). However, due to the highly polymorphic nature of the HLA locus [59], variant detection in these regions is particularly error-prone and results should be cautiously interpreted as potential artifacts. For HLA class I loci, suitable computational pipelines for mutation detection are available [60], whereas for HLA class II to the best of our knowledge this is not the case. Overall, only 1.5% (6/392) of Var^exp^-containing genes were shared among > 2 patients and only one single mutation (in *NBPF1*; Chr. 1:*16891365 G>T)* reoccurred identically in three patients. Considering established driver mutations included in the Cancer Gene Census ( [61]; https://cancer.sanger.ac.uk/census), we observed respective Var^ns^ in most of the HCCs (*n* = 9; 1–3 Var^ns^ per patient), foremost the previously mentioned gene *CTNNB1* (*n* = 8) but also the *androgen receptor*, *mediator complex subunit 12* (*MED12*), *nuclear receptor corepressor 1* (*NCOR1*), *neurogenic locus notch homolog protein 1* (*NOTCH1*) (all *n* = 2), and *NOTCH2/PIK3CA* (*n* = 1). Nevertheless, except from *CTNNB1*, Var^ns^ comprised in the Cancer Gene Census appeared rather infrequently among the examined HCCs.

### Discovery of mutation-derived HLA ligands on different omics levels

#### Exome

In a first step, we sought to assess the number of neoepitopes (PNE) per patient predicted to bind to each individual set of HLA class I alleles, using established binding predictions. On average, 244 ± 77 PNE per HCC patient were predicted from 66 ± 19 Var^ns^, exceeding the respective binding thresholds (Fig. 1c; left panel). The observed increase in PNE numbers compared to Var^ns^ is explained by the fact that Var^ns^ may give rise to multiple PNE due to the shifting window approach used with different peptide lengths (8–11 amino acids) as well as the HLA-binding prediction for up to six individual HLA alleles. Comparing the numbers of PNE to the numbers of protein-altering variants (Var^ns^), this resulted in a very weak correlation (Pearson’s correlation coefficient *r* = 0.38).

#### Transcriptome

When accounting for supplemental evidence for PNE on RNA level, numbers of predicted peptides (PNE^exp^) decreased by half (49 ± 8% of PNE), yielding an average of 118 ± 40 PNE^exp^ per patient. The correlation between expressed protein-changing genomic variants (Var^exp^) and PNE^exp^ also remained moderate (Pearson’s correlation coefficient *r* = 0.50) (Fig. 1c; right panel).

#### Proteome

In order to gain additional protein level evidence for PNE^exp^, we annotated all PNE with log2-intensities from shotgun proteome data (*n* = 7) of HCCs. In this way for a total of 159 PNE (17 ± 14% of PNE^exp^), supportive protein level data was available (on average for 23 ± 21 PNE per patient), mapping to various source proteins (see Additional file 1: Table S5). Only in one patient, no evidence for PNE corresponding to any detected source protein was found (HCC034), yet on average a fraction of 10% (9.8 ± 8.6%) of PNE were supported by additional evidence for occurrence of their source proteins (*n* = 33) on shotgun proteome level.

#### HLA ligandome

To directly assess the presence of mutated HLA ligands, we used the well-established technique of UHPLC-coupled MS/MS to identify naturally presented HLA ligands from HCCs and non-malignant liver tissues. These analyses yielded on average 1403 ± 621 HLA class I-associated peptides from HCC and 1159 ± 525 peptides from non-malignant liver tissue (FDR 5%, length 8–11 amino acids; see also Additional file 3: Figure S1). On average, 51 ± 11% of these peptides were shared between matching malignant and non-malignant liver tissue. When predicting HLA class I binding affinities and filtering for MS-detected peptides exceeding the respective binding threshold for the patients’ HLA allotypes (see Additional file 1: Table S3), on average 1026 ± 451 peptides per tumor (73 ± 10%) and 867 ± 450 peptides per non-malignant liver sample (72% ± 11%) showed HLA-binding properties. This filtering step was performed to enrich for high probability HLA class I ligands, excluding contaminant peptides from downstream analyses. On average, 58 ± 12% of those peptides occurred both in matched malignant and non-malignant liver tissues.

Importantly, we did not find any evidence for naturally presented mutated HLA ligands (NE^lig^) in HCCs, independent of filtering criteria. However, in two HCC patients, we identified one wild-type sequence HLA ligand (WT^lig^) each, corresponding to a PNE.

### Benchmarking HCC and melanoma (Mel) HLA ligandomics datasets

**Fig. 2:**
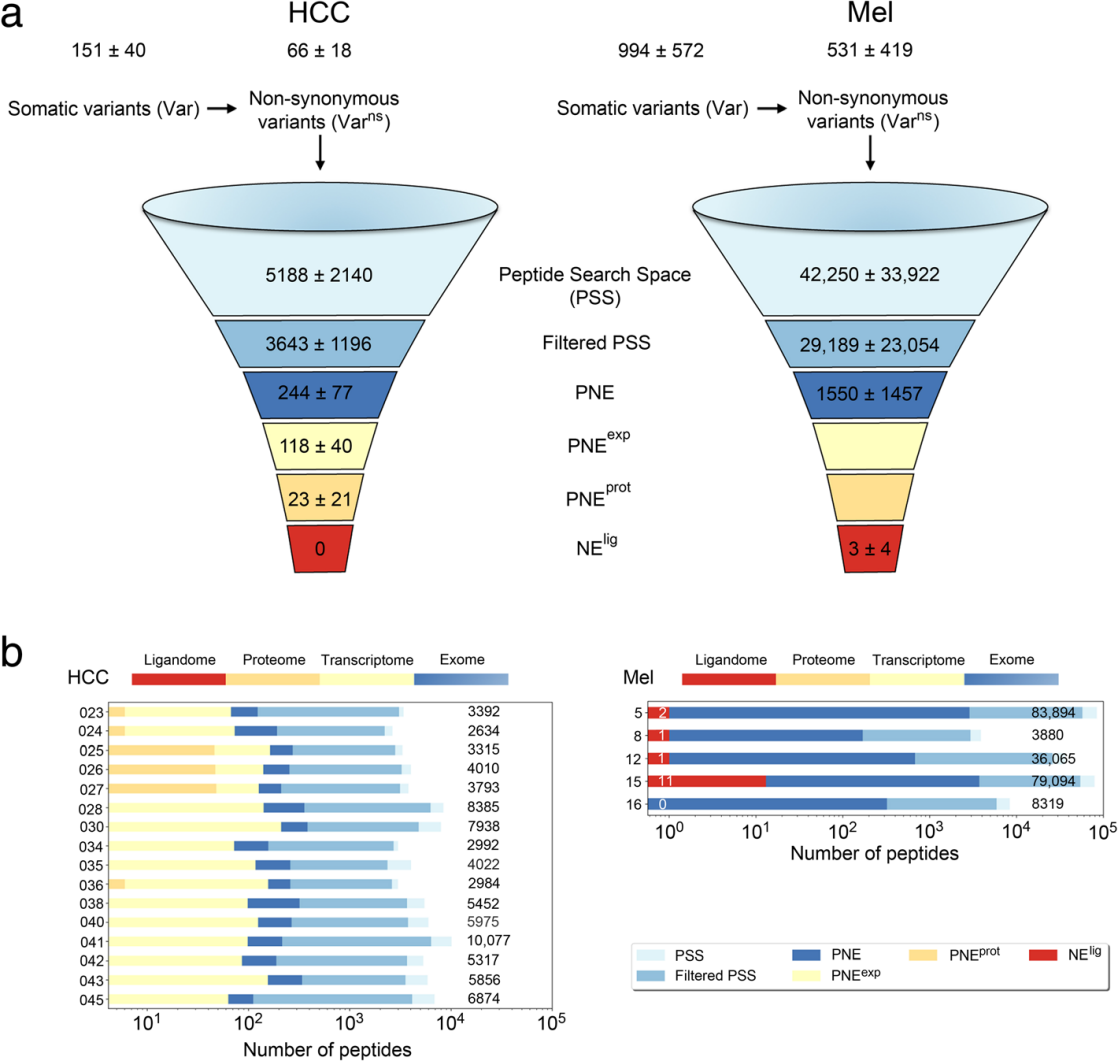
Numbers of predicted neoepitopes with evidence on different omics levels. **a** Numbers of somatic variants and non-synonymous somatic variants (Var and Var^ns^), respectively; peptide search space (PSS), predicted HLA-binding neoepitopes (PNE), and PNE on the different available omics levels: expressed PNE (PNE^exp^), PNE with evidence on shotgun proteome level (PNE^prot^), and neoepitopes observed as natural HLA ligands (NE^lig^) are shown for the HCC dataset (left; *n* = 16) and the Mel dataset (right; *n* = 5) published previously by Bassani-Sternberg et al. [24]. Numbers are given as mean ± SD. **b** Numbers of peptides after processing with our neoepitope identification pipeline are shown on a per patient basis according to the different omics levels as observed in the HCC dataset (left) as well as the Mel dataset (right). For each patient, total counts of predicted peptides (PSS) are annotated in black, numbers of NE^lig^ for Mel patients are shown in red (median = 1.0)

To demonstrate the high sensitivity of our neoepitope identification pipeline, we additionally processed a publicly available dataset of somatic variants from five Mel patients as a reference [24]. The numbers of Var^ns^ and PNE in Mel (Fig. 1d; red dots) showed remarkable differences from our HCC dataset (blue dots). Whereas in two cases, Mel samples showed comparable properties to the HCCs analyzed with respect to the numbers of Var^ns^ and resulting PNE (Mel8, Mel16), these counts were substantially higher in the majority of Mel samples (Mel5, Mel12, Mel15). This resulted in an average number of 531 Var^ns^ in Mel in comparison to only 66 Var^ns^ in HCC, corresponding to an eightfold increased mutated peptide search space (PSS) in Mel. Derived predicted neoepitopes amount to an average of 243 PNE in HCC in contrast to 1550 PNE in the Mel data (Fig. 2a), resulting from a tenfold increased TMB in Mel (on average: 19.06 ± 13.97 per megabase; see Additional file 1: Table S4).

On a per patient basis (Fig. 2b), the HCC dataset proved much more homogenous (PSS: ~ 2500 to 10,000; PNE: 111 to 382) than the Mel data, where the PSS ranged from 4000 to 84,000 (PNE: 169 to 3717).

**Fig. 3:**
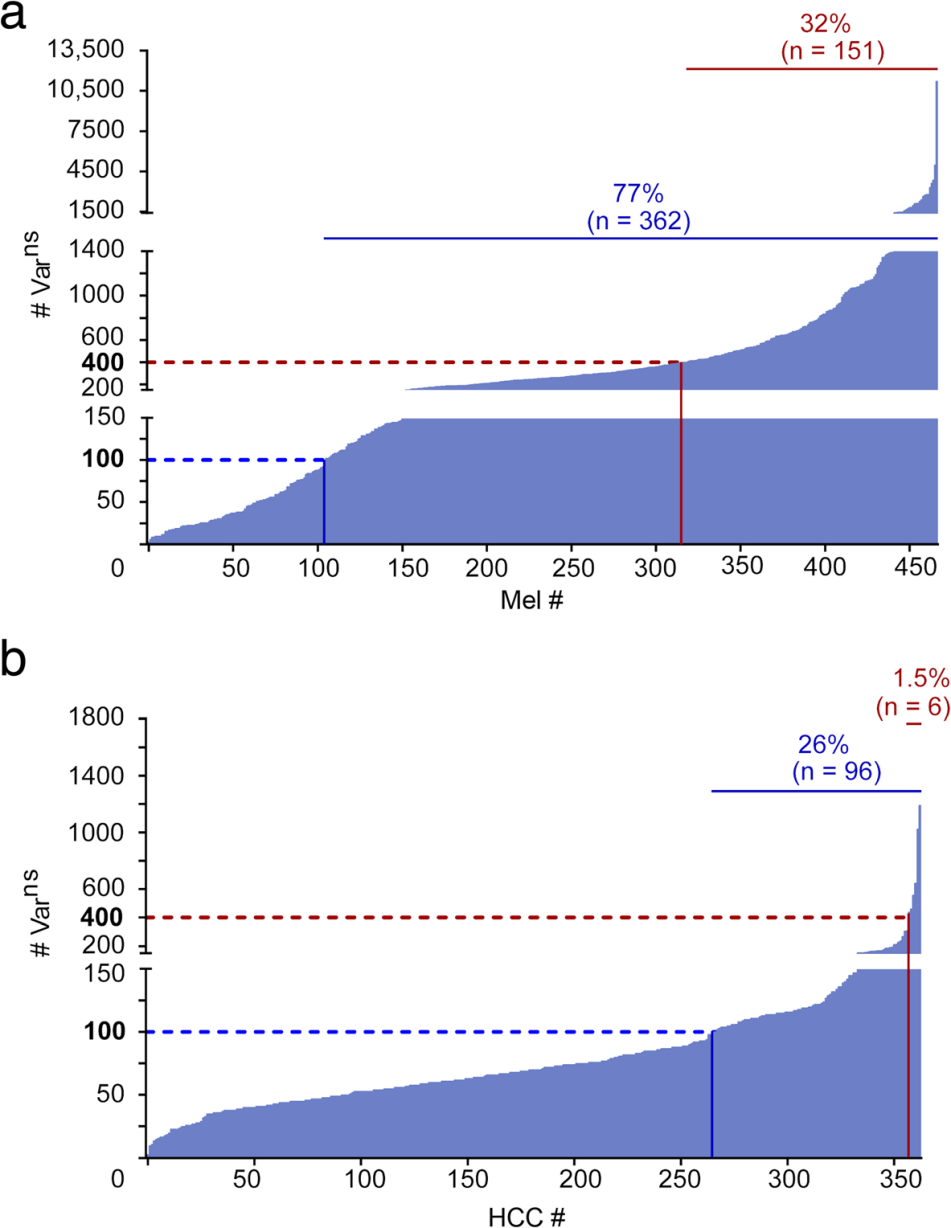
Comparison of the mutational burden in Mel and HCC. **a** Number of mutations (# Var^ns^) of TCGA cases in Mel (*n* = 476). **b** Number of mutations (# Var^ns^) of TCGA cases in HCC (*n* = 363). The data were retrieved from Genomics Data Commons Data Portal (https://portal.gdc.cancer.gov/, access date: 2018-09-16). Variants were filtered for missense variants, frameshift variants, inframe deletions, inframe insertions, and coding sequence variants. Variants that were called by Mutect2 are considered. The number of mutations was assessed with respect to high tumor mutational burden (> 400 Var^ns^, red) and the fraction of tumors with > 100 Var^ns^ (blue)

This was corroborated by analyzing datasets from The Cancer Genome Atlas (TCGA; https://cancergenome.nih.gov/) for both entities, showing a mean number (± SD) of Var^ns^ of 90 ± 100 for HCC (*n* = 363) and 461 ± 761 for Mel (*n* = 467) (Additional file 3: Figure S2). Assessing only the fraction of tumors with > 100 Var^ns^ as suggested previously [12], this amounted to a share of 26% in HCC vs. 77% in Mel. Selecting the share of malignancies that exhibits a high TMB as defined by Goodman et al., we observed merely 1.5% of high TMB tumors among HCC vs. 32% among Mel [62] (Fig. 3).

Employing our HLA ligandomics identification pipeline, we were able to reconfirm all of the NE^lig^ that had been discovered previously by Bassani-Sternberg et al. in their MS dataset (Mel5 (*n* = 2); Mel8 (*n* = 1); Mel15 (*n* = 8)) [24, 63]. Furthermore, we discovered one additional NE^lig^ for Mel12 and three additional NE^lig^ for Mel15 that could be validated by matching spectra from synthetic peptides (see Additional file 1: Table S6). Only one of those NE^lig^ was discovered in a sample (Mel8) with properties comparable to our HCC cohort. Importantly, all other NE^lig^ identified on MS level (10/11) were identified on Mel with high TMB (Fig. 2b). Therefore, it can be stated that our comparatively homogenous HCC cohort, for which no NE^lig^ could be discovered, differs substantially (by at least one order of magnitude concerning TMB) from the properties of Mel patients previously published [24]. This notion is supported by a thorough comparison of both datasets as shown in Fig. 2, as well as by comparisons with comprehensive TCGA datasets (Fig. 3 and Additional file 3: Figure S2).

### Evidence for mutated proteins on shotgun proteome level

**Fig. 4:**
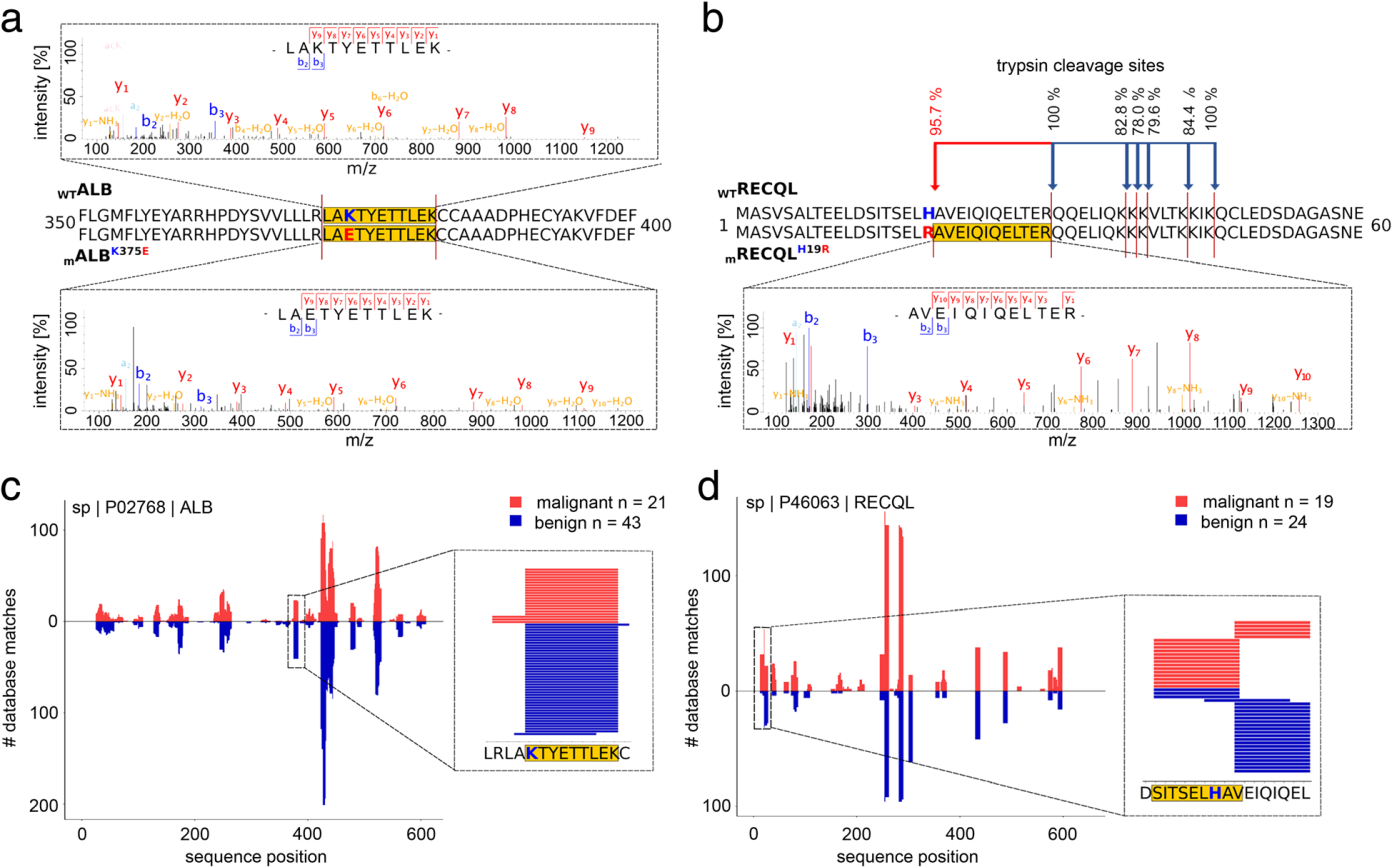
Evidence for mutated proteins in the shotgun proteome and database matching. **a** Annotated spectra of albumin (ALB) showing sequences of wild-type (LAKTYETTLEK; top) and mutated (LAETYETTLEK; bottom) protein measured by LC-MS/MS. **b** Annotated spectra of RecQ like helicase (RECQL) showing sequences of the peptide AVEIQIQELTER resulting from an additional tryptic cleavage side added directly in front of this sequence through a mutation from histidine to arginine, evidenced in HCC tissue only. **c** Database matching of natural HLA ligands with wild-type peptide sequence (with diverse HLA restrictions) covering the exact position evidenced as mutated in ALB. **d** Database matching of natural HLA ligands with wild-type peptide sequence (with diverse HLA restrictions) covering the exact position evidenced as mutated in RECQL

To obtain the best available evidence for the presence of mutated proteins, in absence of tangible data on HLA ligandome level (NE^lig^), we employed shotgun proteomics in HCC tissue samples. To this end, we used a tryptic digest of cell lysates, aware that detection of respective variants is difficult and technology-related sensitivity limitations apply [64] that are governed by a variety of influencing factors and the fraction of genomic alterations detectable on protein level by this approach was reported to be about 2% [65]. Indeed, we discovered one somatic mutation in albumin (ALB^K375E^) on proteome level represented by the tryptic peptide LAETYETTLEK in HCC025 (Fig. 4a), which was corroborated on both exome (Var^ns^) and transcriptome (Var^exp^) levels. Strikingly, we not only detected the tryptic wild-type peptide LAKTYETTLEK but unexpectedly also the mutation-derived peptide LAETYETTLEK in the proteome of non-malignant liver tissue. To investigate the source of this unexpected finding, we obtained two additional serum samples at different time points from the patient and performed shotgun proteomics on them. Patient HCC025 showed tumor recurrence and active disease at both time points and the mutated peptide was detected in both samples, proving that the tumor synthesized a mutated ALB protein secreted into circulation. For HCC026, a Var^exp^ in the ATP-dependent DNA helicase Q1 (RECQL; H19R) could be verified based on an additional tryptic cleavage site introduced through the arginine gained by mutation, which resulted in the proteotypic peptide AVEIQIQELTER. This peptide was not detected in the corresponding non-malignant liver tissue (Fig. 4b).

### Targeted mass spectrometry for discovery of mutated HLA ligands

As NE^lig^ could not be confirmed in HLA ligandomics data of HCC obtained by data-dependent acquisition mode tandem mass spectrometry (DDA-MS/MS), we adopted measures to avoid limitations by missing values and semi-random sampling inherent to this approach [66]. Thus, we attempted to corroborate the PNE^prot^ observed in ALB and RECQL by targeted MS approaches as well as other carefully selected PNE^exp^ in three chosen patients. We selected sets of PNE from three HCCs (HCC025–27) for a selected ion monitoring (SIM) approach using heavy isotope-labeled peptides as a reference to increase the sensitivity for the MS/MS method and improve the probability of detection (Additional file 1: Table S7). Nevertheless, we could not validate any of the candidates and comparisons of low confidence annotations with synthetic peptides did not yield evidence for peptide presentation.

Since peptides harboring the mutations confirmed by proteomics (PNE^prot^) seemed of particular interest (i.e., ALB^K375E^ in HCC025 and RECQL^H19R^ in HCC026), we additionally performed parallel reaction monitoring (PRM) targeted tandem MS (tMS2) measurements targeting the best ranking PNE as well as corresponding wild-type HLA ligands (WT^lig^), covering the mutation site (for details, see Additional file 2). Despite a high number of HLA class I peptides in DDA-MS/MS (HCC025 malignant: 5063; HCC025 non-malignant: 1497; HCC026 malignant: 3678; >HCC026 non-malignant: 3197), PRM tMS2 could not corroborate any of the PNE^prot^ (Fig. 4) as naturally presented HLA ligands in HCC (Additional file 1: Table S8 & Table S9).

### Prioritizing predicted mutated HLA ligands in absence of HLA ligandome evidence

**Fig. 5:**
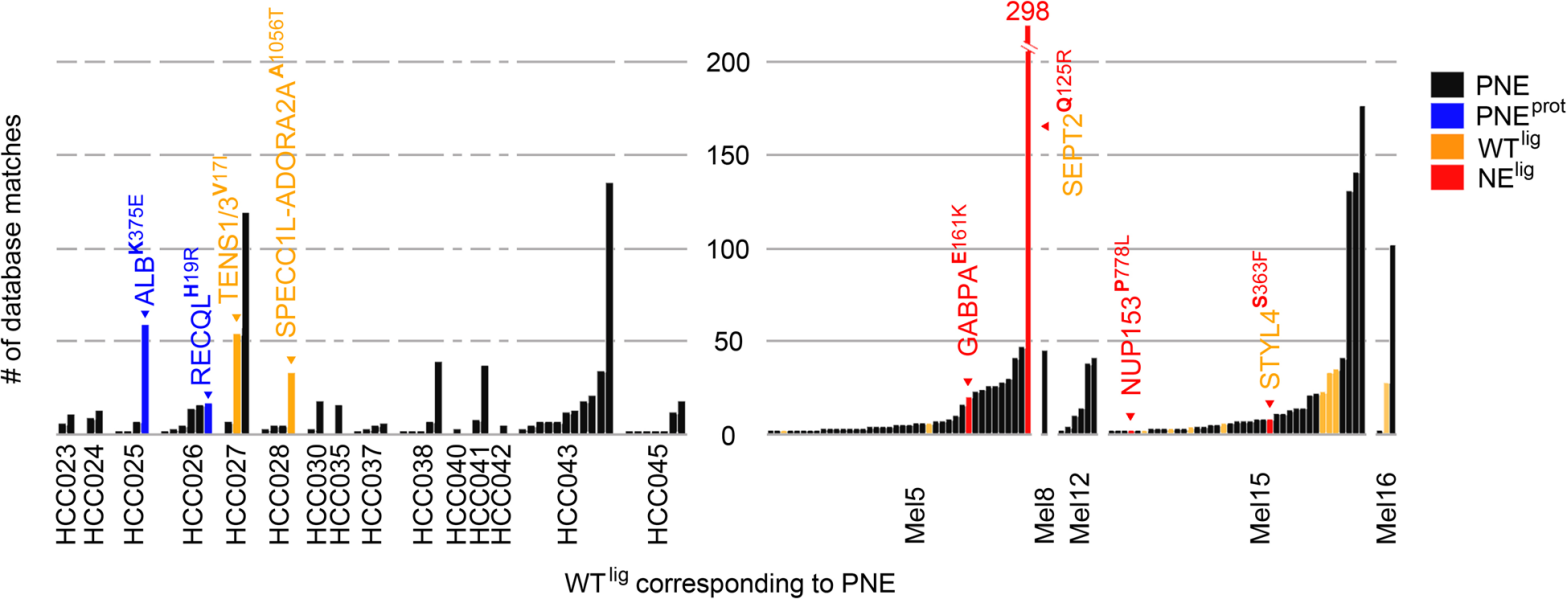
Number of database matches of wild-type ligands (WT^lig^) corresponding to predicted mutated neoepitopes (PNE). PNE with additional evidence in HCC and Mel [24] are highlighted: (1) black: wild-type sequence of PNE contained in database; (2) yellow: wild-type sequence peptide corresponding to PNE confirmed in autologous tissue as natural HLA ligand by mass spectrometry; (3) blue: mutated protein confirmed by shotgun proteomics - PNE^prot^; (4) red: PNE confirmed as natural HLA ligand by mass spectrometry - NE^lig^

Lacking detection of mutated HLA ligands (NE^lig^) does not equal their absence due to several reasons: *inter alia* (1) detection limits of the LC-MS/MS instrumentation, (2) lacking ionizability of respective peptides, (3) particularly strongly hydrophilic and hydrophobic peptides may be missed by the UHPLC method, (4) unknown temporal dynamics of the HLA ligandome [67]. As one way for PNE prioritization, we propose a knowledge-based approach using previously measured wild-type HLA ligands (WT^lig^). Hence, we assumed that the more frequently a WT^lig^ was already detected as a natural HLA ligand by MS the more likely its corresponding NE^lig^ counterpart should exist, provided that the mutation does not negatively impact its HLA-binding affinity, or the respective HLA allele was lost. To this end, we compared the number of database matches of all WT^lig^ in HCC and Mel to an in-house database of HLA ligands measured over the last decades (Fig. 5, Additional file 1: Table S10 & Table S11). Almost all of the malignancies assessed carry at least one mutation (Var^ns^) that could potentially give rise to a PNE whose corresponding WT^lig^ was previously measured multiple times as an HLA-eluted ligand by MS. Interestingly, four of the 15 MS-detected NE^lig^ in Mel support this approach, since also their corresponding WT^lig^ produced multiple hits in our database, including GA-binding protein alpha chain (GABPA; 20 matches), synaptotagmin like 4 (SYTL4; 8 matches), nucleoporin 153 (NUP153; 2 matches), and outstandingly septin 2 (SEPT2; 298 matches). Moreover, the two pinpointed PNE^prot^ in ALB (59 matches) and RECQL (17 matches) give rise to the two most frequently contained WT^lig^ of the respective patients in our database. In addition, WT^lig^ tensin 1/3 (TENS1/3; 54 matches; HCC027) and SPECC1L-ADORA (33 matches; HCC028) were detectable in the respective tumor HLA immunoprecipitates, proving that at least the wild-type sequence peptide is processed and presented on HLA. Speculatively, this might favor the presentation of their NE^lig^ counterparts on HLA ligandome level, although it could not be detected by us. The four mentioned WT^lig^ from HCC (ALB; RECQL; TENS1/3; SPECC1L-ADORA) and two from Mel (SEPT2; SYTL4) have also been documented in the immune epitope database (https://www.iedb.org/ access date: September 2018), which might also guide the way. Ultimately, these results may question HLA ligandome level detection depth and call for establishing large community-based HLA peptidomics databases [67, 68], since individual attempts do not seem reasonable, given the extent of this task.

### Narrowing the scope on alternative (immunological) targets

As alternative targets among HLA ligands with potential therapeutic relevance, we screened our HCC dataset for proteins previously described as cancer-testis antigens (CTA) and found eight different HLA class I ligands mapping to six CTA. These few CTA encompass ARMC3 (Q5W041), ATAD2 (Q6PL18), MAEL (Q96JY0), PRAME (P78395), proteins of the SSX family, and TFDP3 (Q5H9I0) (Table 2).

**Table 2:** Cancer-testis antigens covered by HLA ligands detected in HCC

#	CTA (UniprotID)	Peptide (HLA-)	Sample ID HCC-
1	ARMC3 (Q5W041)	EQIEDLAKY (A*26:01)	045
2	ATAD2 (Q6PL18)	AYAIIKEEL (A*24:02) AEFRTNKTL (B*44:03)	023 045
3	MAEL (Q96JY0)	MVVLDAGRY (A*26:01)	045
4	PRAME (P78395)	SLLQHLIGL (B*08:01)	041
5	SSX1 (Q16384)	AFDDIATYF (C*04:01)	035
6	SSX^●^	RLRERKQLV (B*08:01)	041
7	TFDP3 (Q5H9I0)	EVVGELVAKF (A*26:01)	045

^●^Peptide maps to SSX1 (Q16384); SSX2 (Q16385); SSX3 (Q99909); SSX4 (O60224); SSX6 (Q7RTT6); SSX7 (Q7RTT5); SSX9 (Q7RTT3)

Further, we identified a limited number of CTA among different patients on shotgun proteome level (Additional file 1: Table S12).

Additionally, gene expression analysis revealed 213 differentially expressed (DE) genes, resulting from comparison of autologous tumor and non-malignant tissues. All but one DE gene showed downregulation when compared to matching non-malignant liver (Additional file 3: Figure S3). Respective results indicate apparent differences in the underlying gene expression patterns of tumor and non-malignant liver samples, corroborating the separation of the tumor and non-malignant liver samples in the PCA (on principal component one level; Additional file 3: Figure S4). Visualization by heatmaps and accompanying dendrograms clearly support these observations and show that tumor and non-malignant tissue samples mostly separate in two distinct clades. This separation is seen in most tumor samples except for six patients (HCC024/ 028/ 034/ 035/ 043/ 045), which rather group with the non-malignant tissue samples on the heatmap. To benchmark results from DE expression analysis to publicly available RNA-Seq datasets of HCC, we used recount2, a multi-experiment resource of analysis-ready RNA-Seq datasets with the R package recount. We performed a simple pairwise comparison of the TCGA dataset between non-malignant (*n* = 50) and tumor (*n* = 374) samples using DESeq2. We identified 6044 genes that were DE, based on a statistical significance that was determined by a multiple-testing adjusted *p* value < 0.05 and log2 fold-change > 1 or < − 1. From the 213 DE genes observed in our HCC cohort, about half (*n* = 105) were also found differentially regulated in the TCGA dataset.

Gene functional classification analysis (using DAVID [69, 70]) pointed to mono-oxygenases (CYP450 enzymes) as most prominently inhibited class among DE genes. This gene list was mapped to unique Entrez IDs (*n* = 115), which were mapped to 14 significantly enriched pathways in return (Additional file 1: Table S13).

Finally, we assessed mutations evidenced in our HCC cohort on transcriptome level (Var^exp^) regarding their potential druggability. As previously published [28], also in our HCC patient cohort, mutations druggable by approved pharmaceuticals were missing. Instead, we found one mutation (PIK3CA^E542K^; HCC041; https://www.mycancergenome.org/content/disease/lung-cancer/pik3ca/7/) that has been implicated with lacking drug response to anti-epidermal growth factor (EGFR) antibodies [71].

## Discussion

Neoepitopes, i.e., unique peptides derived from tumor-specific mutations presented as natural HLA ligands and recognized by T cells, have been suggested as highly attractive targets for cancer immunotherapy. It is undisputable that there is mounting (indirect) evidence to suggest that increased numbers of mutations may render malignancies more immunogenic through their neoantigenic repertoire (i.e., mutated HLA ligands) and ultimately more amenable to immunotherapies [9]. Particularly for tumors that are characterized by a high tumor mutational burden (TMB), a correlation with benefits of ICP inhibition has been shown [12–14, 62].

One of the greatest challenges in understanding and ultimately harnessing this neoantigenic repertoire of cancers is the selection and validation of suitable targets from an array of predicted neoepitopes (PNE) derived from computational algorithms [72]. In this connection, it is very plausible to assume that most PNE are irrelevant and would ultimately fail to make an impact on treatment outcomes of individual patients. On the other hand, the selection of a single suitable neoepitope may have unprecedented therapeutic consequences [17, 73] and such a single neoepitope has already been shown to be a target of T cells induced by ICP inhibition [22]. Certainly, this notion is not limited to neoepitopes, but it also applies to tumor-associated antigens, which can possess a comparable immunogenicity [74]. Consequently, non-mutated tumor-specific or highly tumor-associated antigens should be considered prime choice for personalized immunotherapy, when they can be individually validated [75]. Although many assumptions regarding mutated neoepitopes are theoretically and bio-mechanistically plausible [15], there is a fundamental lack of knowledge concerning the precise immunological underpinnings behind tumor specificity [76] and therapeutic implications.

Moreover, biomarkers predicting response to ICP inhibitors with higher precision than TMB [62] are sought-after [77]. A respective biomarker might not only assess the odds for ICP therapy success but may simultaneously allow the development of tailored neoantigen-targeted immunotherapies.

In contrast to the vast array of data available relating to PNE [78, 79], often derived from data of consortia like the International Cancer Genome Consortium (ICGC) or TCGA, current physical evidence for exome-derived mutated HLA ligands (NE^lig^) seems anecdotal (reviewed in [63]) and positive examples for finding this proverbial needle in the haystack are scarce. Hence, to be able to benchmark our results obtained in HCC, we used the best evidence available to us, provided by a dataset from Bassani-Sternberg et al. [24]. Even though this dataset from malignant melanoma (Mel) differs fundamentally from HCCs in a variety of aspects, including *inter alia* a tenfold increased average TMB and a sixfold higher PNE count, this approach enabled benchmarking our pipeline against a dataset containing the required targets (NE^lig^). This notion was also confirmed on a larger scale by TCGA data, corroborating that the average mutation numbers were typically fivefold increased in Mel vs. HCC and the proportion of tumors with high TMB (< 100 Var^ns^) was elevated from 1.5% in HCC to 32% in Mel.

Our HCC dataset is characterized by close to 70 amino acid-changing mutations (Var^ns^) on average translating to a TMB of about two per megabase, numbers corresponding very well with data from a comprehensive set of resectable HCCs [80]. These mutations encompass established hotspots, and a limited number of genes was found to be recurrently mutated [80], affecting the well-established *CTNNB1* primarily but also *NBPF1*. The latter remained the only gene with a repeat identical mutation in our patient cohort, emphasizing that in combination with an individual set of HLA class I allotypes, a neoepitope-targeted therapy needs to be strictly personalized [76]. Since in HCCs only about half of the initially 244 Var^ns^ could be corroborated by RNA level evidence (Var^exp^), this bisected the computationally predicted neoepitope numbers to an average of 118 expressed PNE (PNE^exp^). Further, the correlation of both PNE and PNE^exp^ numbers with mutation counts, showed only a weak correlation. This may imply that there is no direct interconnection between mutation frequency and respective HLA ligands but rather a probabilistic model applies [22], which is governed by the HLA ligandome with distinct rules of presentation [81]. Since we had shotgun proteomics data available, we also assessed whether we could establish any additional physical evidence for the respective source proteins (PNE^prot^) constituting the immediate proteomic context of NE^lig^, which was the case in about one fifth of PNE^exp^ and comprised about 10% of the initial PNE pool. Nevertheless, since this neither implies the actual detection of a mutation in the proteomics dataset (only the identification of at least one tryptic peptide matching the respective protein), nor the HLA presentation of a NE^lig^, we assessed the eluted HLA ligands and searched for any PNE with actual evidence for HLA presentation by LC-MS/MS. Although the ~ 1400 HLA-bound peptides detected on average in HCCs are generally comparable with the numbers previously published in solid cancers [34, 82], they do fall short of the considerable depth reached in Mel, particularly in one single exceptional case, for which more than 20,000 HLA-bound peptides were reported (Mel15; [24]). Since this Mel dataset was available to us and could be processed by our pipeline, we can prove that we would be able to discover NE^lig^ when MS/MS spectra are acquired. In this way, we corroborated all NE^lig^ previously reported [24], as well as four additional NE^lig^ previously unidentified, validated by matching spectra from synthetic peptides. However, in this direct comparison, it becomes particularly clear that Mel and HCC, despite both representing solid tumors, feature fundamental differences on a variety of biological levels. Those differences may imply disparities in antigenicity, determining the odds for immunotherapy success [15]. This notion is confirmed by an extensive analysis of 30 cancer types using comprehensive sequencing data from ICGC and TCGA [26], with striking differences concerning the PNE pool between HCCs and Mel or lung and colorectal cancer [79]. Indeed, we only found a single case with comparable Var^ns^ counts among Mel [24] similar to our relatively homogeneous HCC cohort, where a NE^lig^ could be verified. Hence, chances for presentation of exome-derived NE^lig^ in HCC may be commonly very low, possibly due to cancer immunoediting [83], and this limited target scope may need to be widened to better estimate the odds of neoantigen targeted immunotherapy success in HCC.

This notion is supported by our findings in two out of seven patients where we could confirm a mutation in the proteome, once directly and in the other case through the introduction of an additional tryptic cleavage site by mutation. A comparable approach has been published for rhabdomyosarcoma xenografts, claiming this might be a way to infer relevance for PNE determined by bioinformatics algorithms [78].

Searching for alternatives, we assessed cancer-testis antigens contained among HLA ligands in HCC, which was unrewarding. Furthermore, the odds for administering targeted therapies available to HCC patients in our cohort remained small as previously encountered [28], so we additionally assessed RNA expression and benchmarked data to TCGA datasets to pinpoint signaling pathways that might be harnessed for therapeutic purposes in the future.

## Conclusions

We failed to confirm any exome-derived mutated HLA ligands with sophisticated (targeted) MS approaches in HCC, supporting the assumption that in malignancies with low TMB immunoediting may be a relevant driving force shaping the HLA ligand landscape [84]. Certainly, LC-MS/MS comes with specific limitations that must be considered and HLA ligands may be missed, but it remains the best tool currently available.

Paying close attention to the rules of HLA presentation is important and may support choosing suitable NE^lig^ candidates. We therefore screened our HLA ligand database and found that respective knowledge may indeed guide selection. Undoubtedly, since the HLA ligandome is very complex and our data are limited, the required knowledge needs to be generated in a community effort [85]. Even though our results do suggest there may be relevant value in this approach, the attempt will evidently not solve the underlying probabilistic issues encountered with rare NE^lig^ in HCC. As the scope of our work was primarily focused on simple NE^lig^, derived from exome-derived low complexity variants, our analyses suggest this is only a narrow subset of potential targets that might be used for personalized immunotherapies. Among interesting avenues to be pursued in the future are non-mutated neoantigens [86] and tumor alterations influencing the HLA ligandome composition of malignancies [34], as well as RNA editing and splicing [87, 88], post-translational modifications [89, 90] and targets beyond the exome [91]. In this regard, we may curb the enthusiasm for simple NE^lig^ in HCC, simultaneously suggesting that there is a wide array of alternatives available, which is not even tapped remotely today.

## Additional files

Additional file 1: Supplementary Tables. **Table S1.** Tumor characteristics. **Table S2.** Overview of samples and analyses. **Table S3.** HLA class I allotypes of HCC patients. **Table S4.** Coding variants and tumor mutational burden (TMB) per patient. **Table S5.** Source proteins of predicted mutated neoepitopes (PNE) with evidence on shotgun proteome level. **Table S6.** Identified mutated HLA ligands in the Mel dataset. **Table S7.** Predicted mutated neoepitopes (PNE) tested with selected ion monitoring (SIM) approach. **Table S8.** Predicted mutated neoepitopes (PNE) tested with parallel reaction monitoring (PRM) targeted tandem MS (tMS2) approach. **Table S9.** Parallel reaction monitoring (PRM) in HCC and non-malignant liver tissue samples of patients HCC025 and HCC026. **Table S10.** Database matches of peptides observed in Mel dataset. **Table S11.** Database matches of peptides observed in the HCC cohort. **Table S12.** Cancer testis antigens (CTA) characterized in HCC. **Table S13.** Identified pathways with differentially expressed genes. (PDF 3500 kb)

Additional file 2: Extended Materials and Methods. Comprehensive Materials and Methods section with detailed descriptions of experiments and employed materials and tools. (PDF 516 kb)

Additional file 3: Supplementary Figures. **Figure S1.** HLA ligandomics yields. **Figure S2.** Comparison of the tumor mutational burden in Mel and HCC. **Figure S3.** Differential expression heatmap. **Figure S4.** Principal component analysis (PCA) on principal component one level. (PDF 642 kb)

## Abbreviations

ALB: Albumin
CID: Collision-induced dissociation
CTA: Cancer-testis antigen
DAVID: Database for Annotation, Visualization and Integrated Discovery
DDA: Data-dependent acquisition (MS)
DE: Differentially expressed
FDR: False discovery rate
FPKM: Fragments per kilobase of exon per million reads mapped
HCC: Hepatocellular carcinoma
HPLC: High-performance liquid chromatography
ICP: Immune checkpoint
ID: Identifier
LC-MS/MS: Liquid chromatography-coupled tandem mass spectrometry
LTQ: Linear trap quadrupole
Mel: Malignant melanoma
MS: Mass spectrometry
MS/MS: Tandem mass spectrometry
NE^lig^: Mutated neoantigen with HLA ligandome level evidence (HLA class I)
NGS: Next-generation sequencing
PNE: Predicted mutated Neoepitope
PNE^exp^: Predicted mutated Neoepitope with transcript level evidence
PNE^prot^: Predicted mutated Neoepitope with protein level evidence
ppm: Parts per million
PRM: Parallel reaction monitoring
PSS: Peptide search space
RECQL: ATP-dependent DNA helicase Q1
RT: Retention time
SIM: Selected ion monitoring
TCGA: The Cancer Genome Atlas
TMB: Tumor mutational burden
tMS2: Targeted tandem MS
UHPLC: Ultra-high-performance liquid chromatography
Var: Somatic variant
Var^exp^: Expressed non-synonymous somatic variant
Var^ns^: Non-synonymous somatic variant
WES: Whole exome sequencing
WT^lig^: Wild-type peptide corresponding to PNE with evidence on HLA ligandome level (HLA class I)
