# Supplementary material for: Multi-omics discovery of exome-derived neoantigens in hepatocellular carcinoma
Source: Genome Med. 2019 Apr 30;11:28. doi: 10.1186/s13073-019-0636-8 (PMC6492406; doi:10.1186/s13073-019-0636-8)
Supplement: Supplementary file 1 — Supplementary Tables. Table S1. Tumor characteristics. Table S2. Overview of samples and analyses. Table S3. HLA class I allotypes of HCC patients. Table S4. Coding variants and tumor mutational burden (TMB) per patient. Table S5. Source proteins of predicted mutated neoepitopes (PNE) with evidence on shotgun proteome level. Table S6. Identified mutated HLA ligands in the Mel dataset. Table S7. Predicted mutated neoepitopes (PNE) tested with selected ion monitoring (SIM) approach. Table S8. Predicted mutated neoepitopes (PNE) tested with parallel reaction monitoring (PRM) targeted tandem MS (tMS2) approach. Table S9. Parallel reaction monitoring (PRM) in HCC and non-malignant liver tissue samples of patients HCC025 and HCC026. Table S10. Database matches of peptides observed in Mel dataset. Table S11. Database matches of peptides observed in the HCC cohort. Table S12. Cancer testis antigens (CTA) characterized in HCC. Table S13. Identified pathways with differentially expressed genes. (PDF 3500 kb) [file 13073_2019_636_MOESM1_ESM.pdf]

Multi-omics discovery of exome-derived neoantigens in hepatocellular carcinoma

Markus W. Löffler[1,2,3,4,#], Christopher Mohr[5,6,#], Leon Bichmann[2,7,8], Lena Katharina Freudenmann[2,3], Mathias Walzer[2,7,8,9], Christopher M. Schroeder[10], Nico Trautwein[2], Franz J. Hilke[10], Raphael S. Zinser[2], Lena Mühlenbruch[2], Daniel J. Kowalewski[2,11], Heiko Schuster[2,11], Marc Sturm[10], Jakob Matthes[10], Olaf Riess[10, 12], Stefan Czemmel[6], Sven Nahnsen[6], Ingmar Königsrainer[1], Karolin Thiel[1], Silvio Nadalin[1], Stefan Beckert[1,13], Hans Bösmüller[14], Falko Fend[14], Ana Velic[15], Boris Maček[15], Sebastian P. Haen[2,3,16], Luigi Buonaguro[17], Oliver Kohlbacher[3,5,6,7,8,12,18], Stefan Stevanović[2,3], Alfred Königsrainer[1,3], HEPAVAC Consortium and Hans-Georg Rammensee[2,3]

[1] University Hospital Tübingen, Department of General, Visceral and Transplant Surgery, Hoppe-Seyler-Str. 3, D-72076 Tübingen, Germany
[2] University of Tübingen, Interfaculty Institute for Cell Biology, Department of Immunology, Auf der Morgenstelle 15, D-72076 Tübingen, Germany
[3] German Cancer Consortium (DKTK) and German Cancer Research Center (DKFZ) Partner Site Tübingen, Tübingen, Germany
[4] University Hospital Tübingen, Department of Clinical Pharmacology, Auf der Morgenstelle 8, D-72076 Tübingen, Germany
[5] University Hospital Tübingen, Institute for Translational Bioinformatics, Tübingen, Germany
[6] University of Tübingen, Quantitative Biology Center (QBiC), Auf der Morgenstelle 10, D-72076 Tübingen, Germany
[7] University of Tübingen, Center for Bioinformatics, Sand 14, D-72076 Tübingen, Germany
[8] Department of Computer Science, Applied Bioinformatics, Sand 14, D-72076 Tübingen, Germany
[9] Current address: European Molecular Biology Laboratory, European Bioinformatics Institute (EMBL-EBI), Wellcome Trust Genome Campus, Hinxton, Cambridgeshire, CB10 1SD, United Kingdom
[10] University Hospital Tübingen, Institute of Medical Genetics and Applied Genomics, Calwerstr. 7, D-72076 Tübingen, Germany
[11] Current address: Immatics Biotechnologies GmbH, Paul-Ehrlich-Str. 15, D-72076 Tübingen, Germany
[12] NGS Competence Center Tübingen (NCCT), University of Tübingen, Tübingen, Germany
[13] Current address: Department of General and Visceral Surgery, Schwarzwald-Baar Hospital, Klinikstr. 11, D-78052 Villingen-Schwenningen, Germany.
[14] University Hospital Tübingen, Institute of Pathology and Neuropathology, Liebermeisterstr. 8, D-72076 Tübingen, Germany
[15] University of Tübingen, Interfaculty Institute for Cell Biology, Proteome Center Tübingen (PCT), Auf der Morgenstelle 15, D-72076 Tübingen, Germany
[16] University of Tübingen, Internal Medicine, Department for Oncology, Hematology, Immunology, Rheumatology and Pulmonology, Otfried-Müller-Str. 10, D-72076 Tübingen, Germany
[17] Cancer Immunoregulation Unit, Istituto Nazionale per lo Studio e la Cura dei Tumori, "Fondazione Pascale" - IRCCS, 80131 Naples, Italy
[18] Max Planck Institute for Developmental Biology, Biomolecular Interactions, Spemannstr. 35, D-72076 Tübingen, Germany

Corresponding authors: **Markus W. Löffler, MD**, University of Tübingen, Interfaculty Institute for Cell Biology, Department of Immunology, Auf der Morgenstelle 15, D-72076 Tübingen, Germany, E-mail: **markus.loeffler@uni-tuebingen.de**; and **Christopher Mohr, MSc** (Bioinformatics), University of Tübingen, Quantitative Biology Center (QBiC), Auf der Morgenstelle 10, D-72076 Tübingen, Germany, E-mail: **christopher.mohr@uni-tuebingen.de**

Additional File 1.

Supplementary Tables

Contents

Table S1. *Tumor characteristics.* 3

Table S2. *Overview of samples and analyses.* 4

Table S3. *HLA class I allotypes of HCC patients.* 5

Table S4. *Coding variants and tumor mutational burden (TMB) per patient.* 6

Table S5. *Source proteins of predicted mutated neoepitopes (PNE) with evidence on shotgun proteome level.* 7

Table S6. *Identified mutated HLA ligands in the Mel dataset.* 8

Table S7. *Predicted mutated neoepitopes (PNE) tested with selected ion monitoring (SIM) approach.* 9-13

Table S8. *Predicted mutated neoepitopes (PNE) tested with parallel reaction monitoring (PRM) targeted tandem MS (tMS2) approach.* 14-15

Table S9. *Parallel reaction monitoring (PRM) targeted tandem MS (tMS2) in HCC and non-malignant liver tissue samples of patients HCC025 and HCC026.* 16-21

Table S10. *Database matches of peptides observed in Mel dataset.* 22-28

Table S11. *Database matches of peptides observed in the HCC cohort.* 29-33

Table S12. *Cancer-testis antigens (CTA) characterized in HCC.* 34

Table S13. *Identified pathways with differentially expressed genes.* 35

Table S1. *Tumor characteristics.*

TNM tumor staging (according to Union internationale contre le cancer (UICC); https://www.uicc.org/sites/main/files/private/How_to_use_TNM_0.pdf) and grading of HCCs from patients (n=16) contributing biological samples for the study.

Patient ID	T	N	M	G
**HCC023**	pT3	pN0	cM0	2-3
**HCC024**	pT1	pN0	cM0	1
**HCC025**	pT3	pNx	cM0	1-2
**HCC026**	rpT2	pNx	cM0	2
**HCC027**	pT3	pN0	cM0	2-3
**HCC028**	pT1	pNx	cM0	2
**HCC030**	pT3	pNx	cM0	2
**HCC034**	pT3	pNx	cM0	2
**HCC035**	pT1	pNx	cM0	3
**HCC036**	pT3	pN0	cM0	3
**HCC038**	pT1	pNx	cM0	2
**HCC040**	pT1	pN0	cM0	2
**HCC041**	pT2	pNx	cM0	3
**HCC042**	pT2	pNx	cM0	3
**HCC043**	pT1	pNx	cM0	2
**HCC045**	pT1	pNx	cM0	2

Table S2. *Overview of samples and analyses.*

Overview of analyses performed on available tumor (T) and non-malignant liver samples (N) from patients with HCC.

Patient ID	Whole exome sequencing	Transcriptomics	Proteomics	HLA ligandomics		
	T + N	T + N	T + N	Top 5 [a] T + N	SIM [b] T	PRM [c] T + N
**HCC023**	✓	✓	✓	✓	-	-
**HCC024**	✓	✓	✓	✓	-	-
**HCC025**	✓	✓	✓	✓	✓	✓
**HCC026**	✓	✓	✓	✓	✓	✓
**HCC027**	✓	✓	✓	✓	✓	-
**HCC028**	✓	✓	-	✓	-	-
**HCC030**	✓	✓	-	✓	-	-
**HCC034**	✓	✓	✓	✓	-	-
**HCC035**	✓	✓	-	✓	-	-
**HCC036**	✓	T only	✓	✓	-	-
**HCC038**	✓	✓	-	✓	-	-
**HCC040**	✓	✓	-	✓	-	-
**HCC041**	✓	✓	-	✓	-	-
**HCC042**	✓	✓	-	✓	-	-
**HCC043**	✓	✓	-	✓	-	-
**HCC045**	✓	✓	-	✓*	-	-

[a] Top five method in automated data-dependent acquisition (DDA);

[b] Selected ion monitoring (SIM);

[c] Parallel reaction monitoring (PRM)

* multiple T samples

Table S3. *HLA class I allotypes of HCC patients.*

HLA class I allotypes assigned according to Materials and Methods using OptiType for HCC patients (n=16).

Patient ID	HLA-		
	**A***	**B***	**C***
**HCC023**	24:02 / 29:02	37:01 / 44:03	06:02 / 16:01
**HCC024**	03:01 / 68:01	15:01 / 40:01	03:04 / 03:81
**HCC025**	02:01 / 11:01	37:01 / 44:02	06:02 / 07:04
**HCC026**	01:01 / 02:01	08:01 / 51:01	01:02 / 07:01
**HCC027**	03:01 / 24:02	18:01 / 27:05	02:02 / 07:01
**HCC028**	02:01 / 24:02	07:02 / 35:03	04:01 / 07:02
**HCC030**	02:01 / 03:01	14:01 / 27:05	01:02 / 08:02
**HCC034**	11:01 / 23:01	18:01 / 44:03	04:01 / 07:01
**HCC035**	02:01 / 68:01	27:05 / 35:03	02:02 / 04:01
**HCC036**	02:01 / 68:01	27:05 / 44:02	01:02 / 07:04
**HCC038**	02:01	07:02	07:02
**HCC040**	03:01 / 68:01	44:02 / 51:01	07:04 / 15:02
**HCC041**	01:01	08:01	07:01
**HCC042**	01:01 / 03:01	08:01 / 55:01	03:03 / 07:01
**HCC043**	01:01 / 02:01	08:01 / 40:01	03:04 / 07:01
**HCC045**	01:01 / 26:01	44:03 / 47:01	04:01 / 06:02

Table S4. *Coding variants and tumor mutational burden (TMB) per patient.*

Coding variants include synonymous and non-synonymous variants in coding regions. TMB is given as coding variant per megabase, calculated by dividing the number of coding variants by the number of megabases per exome. TMB was estimated as described in Materials and Methods.

ID	Coding variants	TMB
**HCC023**	70	1.40
**HCC024**	70	1.40
**HCC025**	92	1.84
**HCC026**	98	1.96
**HCC027**	102	2.04
**HCC028**	111	1.85
**HCC030**	174	2.90
**HCC034**	75	1.50
**HCC035**	84	1.40
**HCC036**	86	1.72
**HCC038**	109	1.82
**HCC040**	109	1.82
**HCC041**	189	3.15
**HCC042**	89	1.48
**HCC043**	108	1.80
**HCC045**	132	2.20
**Mel5**	1899	37.98
**Mel8**	83	1.66
**Mel12**	811	16.22
**Mel15**	1777	35.54
**Mel16**	195	3.90

Table S5. *Source proteins of predicted mutated neoepitopes (PNE) with evidence on shotgun proteome level.*

Uniprot identifiers with respective gene name of source proteins of PNE assigned to respective patients.

Patient ID	Gene Name	Uniprot Entry
**HCC023**	VPS26A	O75436
**HCC023**	PRRC1	Q96M27
**HCC023**	HUWE1	Q7Z6Z7
**HCC024**	FASN	P49327
**HCC024**	CLTC	Q00610
**HCC025**	PSMD4	P55036
**HCC025**	SERPINI2	O75830
**HCC025**	HADHA	P40939
**HCC025**	ALB	P02768
**HCC025**	SEC63	Q9UGP8
**HCC025**	FGL1	Q08830
**HCC025**	KIAA0196	Q12768
**HCC025**	COG4	Q9H9E3
**HCC025, HCC026**	CTNNB1	P35222
**HCC026**	ANXA11	P50995
**HCC026**	LUM	P51884
**HCC026**	RECQL	P46063
**HCC026**	ACIN1	Q9UKV3
**HCC026**	PLXNB2	O15031
**HCC026**	BPNT1	O95861
**HCC026**	RAD50	Q92878
**HCC026**	SLC4A4	Q9Y6R1
**HCC026**	EEF1A1	P68104
**HCC027**	GPAM	Q9HCL2
**HCC027**	PITRM1	Q5JRX3
**HCC027**	ALDH18A1	P54886
**HCC027**	TXNRD2	Q9NNW7
**HCC027**	HECTD3	Q5T447
**HCC027**	ALDH9A1	P49189
**HCC027**	APOB	P04114
**HCC027**	TNS3	Q68CZ2
**HCC036**	GANAB	Q14697
**HCC036**	FBN1	P35555

Table S6. *Identified mutated HLA ligands in the Mel dataset.*

Identified mutated HLA ligands, validated by matching MS/MS fragment spectra obtained from synthetic peptides, are given for the respective Mel patient with information about their sequence, details on the mutation and HLA restriction. Amino acid exchanges resulting from non-synonymous variants (Var^{ns}) are marked in red.

Patient ID	Neoepitope	Length (AA)	Genomic Position	Gene	Transcript	Mutation AA	HLA restriction
Mel15	GRIAFFLKY	9	X:100687163 G>A	SYTL4	ENST00000263033	p.S363F	B*27:05
	RLFKGYEGSLIK	12	8:30474849 C>T	RBPMS	ENST00000517860	p.P46L	A*03:01
	LPIQYEPVL	9	14:39095964 G>A	SEC23A	ENST00000307712	p.P52L	B*35:03
	RIKQTARK	8	12:31792156 G>A	H3F3C	ENST00000340398	p.T4I	A*03:01
	KLILWRGLK	9	7:158680743 G>A	NCAPG2	ENST00000409339	p.P333L	A*03:01
	KLKLPIIMK	9	14:32822259 G>A	AKAP6	ENST00000280979	p.M1482I	A*03:01
	ASWVVPIDIK	10	14:70733760 C>T	MAP3K9	ENST00000555993	p.E689K	A*03:01
	GRTGAGKSFL	10	10:99845661 C>T	ABCC2	ENST00000370449	p.S1342F	B*27:05
	FVPPTAISHF	10	10:68977540 C>T	DDX21	ENST00000354185	p.S584F	B*35:03
	SRFLSQLDK	9	3:142562852 C>T	ATR	ENST00000350721	p.E183K	B*27:05
	ETLKPGTC*VKR	11	6:17637374 G>A	NUP153	ENST00000537253	p.P778L	A*68:01
Mel8	SPGPVKLEL	9	5:176384171 G>A	NOP16	ENST00000619979	p.P169L	B*07:02
Mel5	ETSKQVTRW	9	21:25752162G>A	GABPA	ENST00000354828	p.E161K	A*25:01
	YIDERFERY	9	2:241337414A>G	SEPT2	ENST00000616972	p.Q125R	A*01:01
Mel12	DFILKPEL	8	19:4445543C>T	UBXN6	ENST00000394765	p.S427F	B*08:01

Table S7. *Predicted mutated neoepitopes (PNE) tested with selected ion monitoring (SIM) approach.*

Heavy isotope-labeled synthetic peptides of PNE for SIM approaches in HCC samples of patients HCC025, HCC026, and HCC027. Indicated m/z values refer to the 2+ precursor ions. Retention times (RT) were assessed in an HLA class I peptide matrix eluted from JY cells. Amino acid exchanges resulting from non-synonymous variants (Var^{ns}) are marked in red.

Protein	UniProt ID	Var^{ns}	Transcripts	PNE	Natural peptide [m/z]	Heavy isotope-labeled peptide [m/z]	RT [min]	SIM scan #	HLA restriction
**HCC025**									
**ALB**	P02768	K375E	ENST00000295897	RLAETYETT[L($^{13}C_6$; ^{15}N)]	598.8115^{++}	602.3201^{++}	46	2	A*02:01
**BLMH**	Q13867	F324V	ENST00000261714	SIKDGEAVW[V($^{13}C_5$; ^{15}N)]	552.2902^{++}	555.2971^{++}	52	3	A*02:01
**DPY19L4**	Q7Z388	K339N	ENST00000414645	QLNVKNGSF[V($^{13}C_5$; ^{15}N)]	553.3037^{++}	556.3105^{++}	43	1	A*02:01
				VKNGSFVAK[I($^{13}C_6$; ^{15}N)]	531.8189^{++}	535.3275^{++}	33	2	A*02:01
				NVKNGSFVA[K($^{13}C_6$; $^{15}N_2$)]	532.2984^{++}	536.3055^{++}	43	2	A*11:01
				LQLN[V($^{13}C_5$; ^{15}N)]KNGSF	560.3115^{++}	563.3184^{++}	50	3	B*37:01
**HADHA**	P40939	G341V	ENST00000380649	VLYHGQV[L($^{13}C_6$; ^{15}N)]	464.7662^{++}	468.2748^{++}	44	3	A*02:01
**PKDREJ**	Q9NTG1	T1875I	ENST00000253255	HIYGSGGYA[L($^{13}C_6$; ^{15}N)]	519.2562^{++}	522.7648^{++}	47	1	A*02:01
**PTPRM**	P28827	Q718R	ENST00000332175<br>ENST00000580170	RVATKGAATP[K($^{13}C_6$; $^{15}N_2$)]	550.3327^{++}	554.3398^{++}	16	3	A*11:01
**SLC38A10**	Q9HBR0	G122R	ENST00000374759<br>ENST00000288439	RLFGFQVG[R($^{13}C_6$; $^{15}N_4$)]	540.3091^{++}	545.3132^{++}	49	2	A*11:01

Protein	UniProt ID	Var^{ns}	Transcripts	PNE	Natural peptide [m/z]	Heavy isotope-labeled peptide [m/z]	RT [min]	SIM scan #	HLA restriction
**SPEN**	Q96T58	P721L	ENST00000375759	RDRDHERR[L($^{13}C_6$; ^{15}N)]	626.8345^{++}	630.3431^{++}	16	2	A*02:01
				LIERSQSP[V($^{13}C_5$; ^{15}N)]	514.7904	517.7973^{++}	32	2	A*02:01
				RLIERSQSP[V($^{13}C_5$; ^{15}N)]	592.8409^{++}	595.8478^{++}	31	1	B*37:01
				LIERSQSPVH[L($^{13}C_6$; ^{15}N)]	639.8619^{++}	643.3705^{++}	40	1	A*02:01
**TDG**	Q13569	G343V	ENST00000392872	YEAAY[V($^{13}C_5$; ^{15}N)]GAY	503.7295^{++}	506.7363^{++}	54	3	B*44:02
**XRCC2**	O43543	I95V	ENST00000359321	RLVTVLEHR[L($^{13}C_6$; ^{15}N)]	618.3828^{++}	621.8914^{++}	45	1	A*02:01
**HCC026**									
**CTNNB1**	P35222	S37Y	ENST00000349496<br>ENST00000396185<br>ENST00000396183<br>ENST00000405570	QSYLDSG[I($^{13}C_6$; ^{15}N)]HY	591.7749^{++}	595.2835^{++}	53	3	A*01:01
**CTNS**	O60931	P80L	ENST00000381870<br>ENST00000046640	LPDEVVVP[L($^{13}C_6$; ^{15}N)]	490.7868^{++}	494.2954^{++}	80	1	B*51:01
			ENST00000381870<br>ENST00000046640	ELPDEVVVP[L($^{13}C_6$; ^{15}N)]	555.3081^{++}	558.8167^{++}	84	1	A*02:01
**EEF1A1**	P68104	E68Q	ENST00000309268<br>ENST00000316292<br>ENST00000331523	KLKAERQRG[I($^{13}C_6$; ^{15}N)]	599.8726^{++}	603.3812^{++}	20	3	A*02:01
**KMT2E**	Q8IZD2	A445P	ENST00000311117<br>ENST00000257745<br>ENST00000334877<br>ENST00000476671	IT[I($^{13}C_6$; ^{15}N)]PFDFDY	565.7739^{++}	569.2824^{++}	95	2	A*01:01

Protein	UniProt ID	Var^{ns}	Transcripts	PNE	Natural peptide [m/z]	Heavy isotope-labeled peptide [m/z]	RT [min]	SIM scan #	HLA restriction
**MPDZ**	O75970	I149V	ENST00000319217<br>ENST00000541718<br>ENST00000381022<br>ENST00000381015	IAVSEEDT[L($^{13}C_6$; ^{15}N)]	488.7453^{++}	492.2539^{++}	52	2	B*51:01
				GIAVSEEDT[L($^{13}C_6$; ^{15}N)]	517.2560^{++}	520.7646^{++}	56	1	A*02:01
**PLXNB2**	O15031	A1554T	ENST00000359337<br>ENST00000449103	GTTLILSK[V($^{13}C_5$; ^{15}N)]	466.2948^{++}	469.3016^{++}	51	1	A*02:01
**RECQL**	P46063	H19R	ENST00000444129<br>ENST00000421138	SITSELRA[V($^{13}C_5$; ^{15}N)]	488.2771^{++}	491.284^{++}	50	2	A*02:01
**SLC4A4**	Q9Y6R1	A973V	ENST00000425175<br>ENST00000264485	WILKSTVA[V($^{13}C_5$; ^{15}N)]	508.8106^{++}	511.8175^{++}	61	1	A*02:01
				ILKSTVAV[I($^{13}C_6$; ^{15}N)]	472.3130^{++}	475.8215^{++}	50	1	B*08:01
				TVAVIIFP[V($^{13}C_5$; ^{15}N)]	479.8022^{++}	482.8091^{++}	94	1	A*02:01
				WILKSTVAV[I($^{13}C_6$; ^{15}N)]	565.3526^{++}	568.8612^{++}	72	3	A*02:01
**SPECC1L**	Q69YQ0	I771V	ENST00000314328<br>ENST00000437398<br>ENST00000541492	DIKSEAQEE[V($^{13}C_5$; ^{15}N)]	574.2775^{++}	577.2844^{++}	41	3	A*02:01
**STK38**	Q15208	W271L	ENST00000229812	NSKRKAET[L($^{13}C_6$; ^{15}N)]	523.8013^{++}	527.3099^{++}	17	3	B*08:01
**STK17B**	O94768	L143F	ENST00000263955<br>ENST00000409228	RLIKQIFEG[V($^{13}C_5$; ^{15}N)]	601.8664^{++}	604.8733^{++}	57	2	A*02:01
				LIKQIFEG[V($^{13}C_5$; ^{15}N)]	523.8159^{++}	526.8227^{++}	63	2	A*02:01
				QIFEGVYY[L($^{13}C_6$; ^{15}N)]	566.2897^{++}	569.7983^{++}	85	2	A*02:01
**YIPF2**	Q9BWQ6	P310L	ENST00000253031<br>ENST00000586748	NIALSPTL[L($^{13}C_6$; ^{15}N)]	471.2869^{++}	474.7955^{++}	80	2	A*02:01
				ALSPTLLQS[L($^{13}C_6$; ^{15}N)]	521.8108^{++}	525.3194^{++}	26	3	A*02:01

Protein	UniProt ID	Var^{ns}	Transcripts	PNE	Natural peptide [m/z]	Heavy isotope-labeled peptide [m/z]	RT [min]	SIM scan #	HLA restriction
**HCC027**									
**ABCC6**	O95255	Q715E	ENST00000205557	GEELDPPW[L($^{13}C_6$; ^{15}N)]	528.2558^{++}	531.7644^{++}	143	3	B*18:01
**ARL6**	Q9H0F7	L146F	ENST00000335979<br>ENST00000463745<br>ENST00000394206	AVTSVK[V($^{13}C_5$; ^{15}N)]SQF	533.3006^{++}	536.3075^{++}	80	2	A*03:01
**GPAM**	Q9HCL2	H233Y	ENST00000348367<br>ENST00000423155	HRSYIDYL[L($^{13}C_6$; ^{15}N)]	590.3115^{++}	593.8201^{++}	107	1	B*27:05
				LLF[L($^{13}C_6$; ^{15}N)]PVHRSY	622.8611^{++}	626.3697^{++}	98	3	A*03:01
				LFLPVHRSY[I($^{13}C_6$; ^{15}N)]	622.8611^{++}	626.3697^{++}	98	2	A*24:02
				SYIDYLL[L($^{13}C_6$; ^{15}N)]TF	624.3315^{++}	627.8401^{++}	160	1	A*24:02
**NFE2L2**	Q16236	D77Y	ENST00000397062	Q[L($^{13}C_6$; ^{15}N)]YEETGEF	558.2482^{++}	561.7568^{++}	101	1	A*03:01
				YEETGEFLP[I($^{13}C_6$; ^{15}N)]	599.2873^{++}	602.7959^{++}	141	3	B*18
				LYEETGEF[L($^{13}C_6$; ^{15}N)]	550.7610^{++}	554.2695^{++}	96	1	A*24:02
				LYEETGEFLP[I($^{13}C_6$; ^{15}N)]	655.8294^{++}	659.3380^{++}	124	3	A*24:02
**TMEM57**	Q8N5G2	R490W	ENST00000374343	EEATAAWA[V($^{13}C_5$; ^{15}N)]	474.2271^{++}	477.2340^{++}	105	3	B*18:01
**TNS3**	Q68CZ2	I17V	ENST00000311160<br>ENST00000398879<br>ENST00000442536<br>ENST00000458317	TYITERIVA[V($^{13}C_5$; ^{15}N)]	582.8348^{++}	585.8417^{++}	102	2	A*24:02
				TER[I($^{13}C_6$; ^{15}N)]VAVSF	511.2875^{++}	514.7961^{++}	99	2	B*18:01

Protein	UniProt ID	Var[ns]	Transcripts	PNE	Natural peptide [m/z]	Heavy isotope-labeled peptide [m/z]	RT [min]	SIM scan #	HLA restriction
**TXNL4A**	P83876	N70S	ENST00000269601	DITEVPDFS[K($^{13}C_6$; $^{15}N_2$)]	575.7850^{++}	579.7921^{++}	99	1	A*03:01
**WDR3**	Q9UNX4	Y588H	ENST00000349139	FLSLHGH[K($^{13}C_6$; ^{15}N2)]	469.7640^{++}	473.7711^{++}	42	2	A*03:01
				FFLSLHGHK[L($^{13}C_6$; ^{15}N)]	599.8402^{++}	603.3488^{++}	87	1	A*24:02
**ZNF407**	Q9C0G0	D1779N	ENST00000299687<br>ENST00000577538	NYGTNVP[V($^{13}C_5$; ^{15}N)]EF	570.272^{++}	573.2789^{++}	118	2	A*24:02

Table S8. *Predicted mutated neoepitopes (PNE) tested with parallel reaction monitoring (PRM) targeted tandem MS (tMS2) approach.*

Heavy isotope-labeled synthetic peptides for PRM tMS2 approaches in HCC and non-malignant liver samples of patients HCC025 and HCC026. For the two mutated antigens with evidence on proteome level (PNE^{Prot}; ALB^{K375E} in HCC025 and $RECQL^{H19R}$ in HCC026), we synthesized PNE as well as corresponding wild-type sequences of the best predicted HLA ligands to perform PRM tMS2. M/z values are indicated for both 2+ and 3+ precursor ions, which were both selected for fragmentation. Retention times (RT) were assessed in an HLA class I peptide matrix eluted from JY cells. Amino acid exchanges resulting from non-synonymous variants (Var^{ns}) are marked in red.

PNE	Natural peptide [m/z]	Heavy isotope-labeled peptide [m/z]	RT [min]	Wild-type sequence	Natural peptide [m/z]	Heavy isotope-labeled peptide [m/z]	RT [min]	HLA restriction
**HCC025: ALB^{K375E} (P02768)**								
AETYETT[L($^{13}C_6$; ^{15}N)]EK	592.7877^{++} 395.5276^{+++}	596.2963^{++} 397.8666^{+++}	37.35	[A($^{13}C_3$; ^{15}N)]KTYETTLEK	592.3139^{++} 395.2117^{+++}	594.3174^{++} 396.5474^{+++}	28.10	A*11:01
V[L($^{13}C_6$; ^{15}N)]LLRLAET	514.3291^{++} 343.2219^{+++}	517.8377^{++} 345.5609^{+++}	71.38	VLLLRL[A($^{13}C_3$; ^{15}N)]KT	513.8553^{++} 342.9060^{+++}	515.8589^{++} 344.2417^{+++}	58.52	A*02:01
ETYETT[L($^{13}C_6$; ^{15}N)]EK	557.2691^{++} 371.8485^{+++}	560.7777^{++} 374.1876^{+++}	35.35	K[T($^{13}C_4$; ^{15}N)]YETTLEK	556.7953^{++} 371.5326^{+++}	559.3006^{++} 373.2028^{+++}	26.29	A*11:01
R[L($^{13}C_6$; ^{15}N)]AETYETTL	598.8115^{++} 399.5434^{+++}	602.3201^{++} 401.8825^{+++}	50.31	RL[A($^{13}C_3$; ^{15}N)]KTYETTL	598.3377^{++} 399.2276^{+++}	600.3412^{++} 400.5633^{+++}	39.40	A*02:01
[L($^{13}C_6$; ^{15}N)]AETYETTLEK	649.3297^{++} 433.2222^{+++}	652.8383^{++} 435.5613^{+++}	45.48	L[A($^{13}C_3$; ^{15}N)]KTYETTLEK	648.8559^{++} 432.9064^{+++}	650.8595^{++} 434.2421^{+++}	35.03	A*11:01

PNE	Natural peptide [m/z]	Heavy isotope-labeled peptide [m/z]	RT [min]	Wild-type sequence	Natural peptide [m/z]	Heavy isotope-labeled peptide [m/z]	RT [min]	HLA restriction
**HCC026: RECQL^{H19R} (P46063)**								
SITSE[L($^{13}C_6$; ^{15}N)]RAV	488.2771^{++} 325.8538^{+++}	491.7857^{++} 328.1929^{+++}	54.66	SITSELH[A($^{13}C_3$; ^{15}N)]V	478.7560^{++} 319.5064^{+++}	480.7596^{++} 320.8421^{+++}	51.69	A*02:01
ITSE[L($^{13}C_6$; ^{15}N)]RAVEI	565.8244^{++} 377.5520^{+++}	569.3330^{++} 379.8911^{+++}	60.29	ITSELH[A($^{13}C_3$; ^{15}N)]VEI	556.3033^{++} 371.2046^{+++}	558.3069^{++} 372.5403^{+++}	57.83	A*02:01
RA[V($^{13}C_5$; ^{15}N)]EIQIQEL	599.8431^{++} 400.2312^{+++}	602.8500^{++} 402.2358^{+++}	63.03	H[A($^{13}C_3$; ^{15}N)]VEIQIQEL	590.3220^{++} 393.8838^{+++}	592.3256^{++} 395.2195^{+++}	62.92	A*02:01

Table S9. *Parallel reaction monitoring (PRM) targeted tandem MS (tMS2) in HCC and non-malignant liver tissue samples of patients HCC025 and HCC026.*

For the two mutated antigens with evidence on proteome level (PNE^{Prot}; ALB^{K375E} in HCC025 and RECQL^{H19R} in HCC026), we performed PRM tMS2 for the best predicted mutated neoepitopes (PNE) as well as corresponding wild-type sequences (WT seq). Amino acid exchanges resulting from non-synonymous variants (Var^{ns}) are marked in red. Transitions of heavy isotope-labeled synthetic peptides measured at 20 fmol [4 fmol/µl] in a matrix of HLA class I peptides eluted from JY cells are shown. The natural counterparts could not be detected in non-malignant or malignant liver samples of HCC025 or HCC026, respectively.

PNE / WT seq.	20 fmol in HLA class I peptide matrix	Non-malignant liver	HCC
**HCC025: ALB^{K375E} (P02768)**			
**AETYETTLEK**	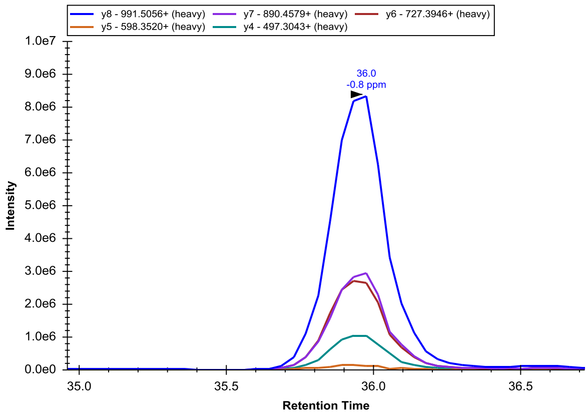	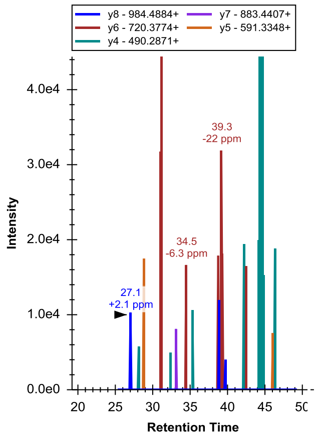	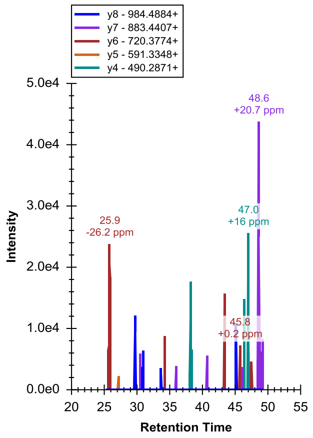
**AKTYETTLEK** (2+ and 3+)	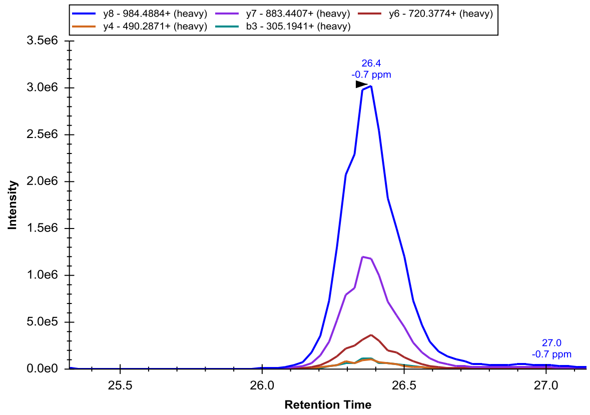	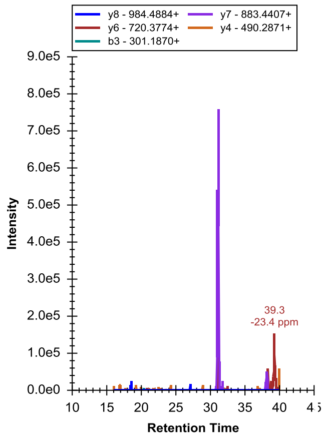	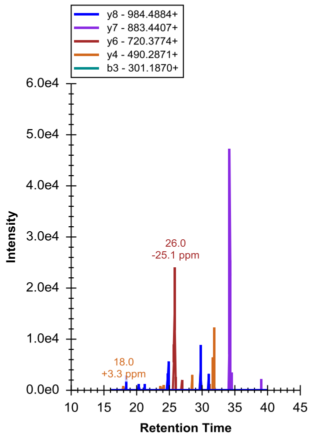
	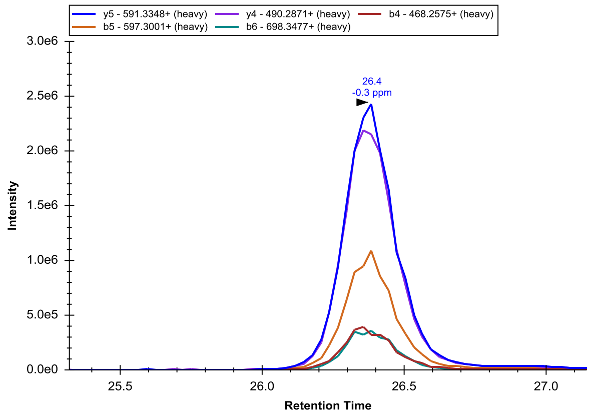	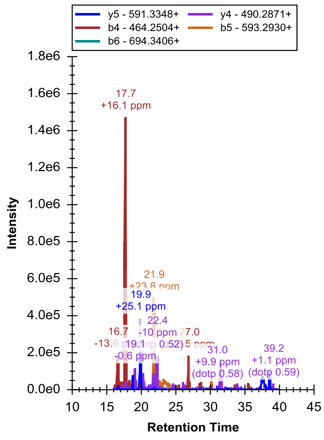	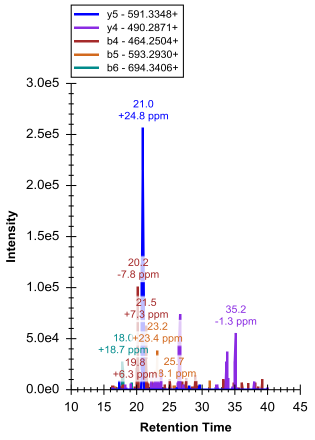

VLLLRLAET

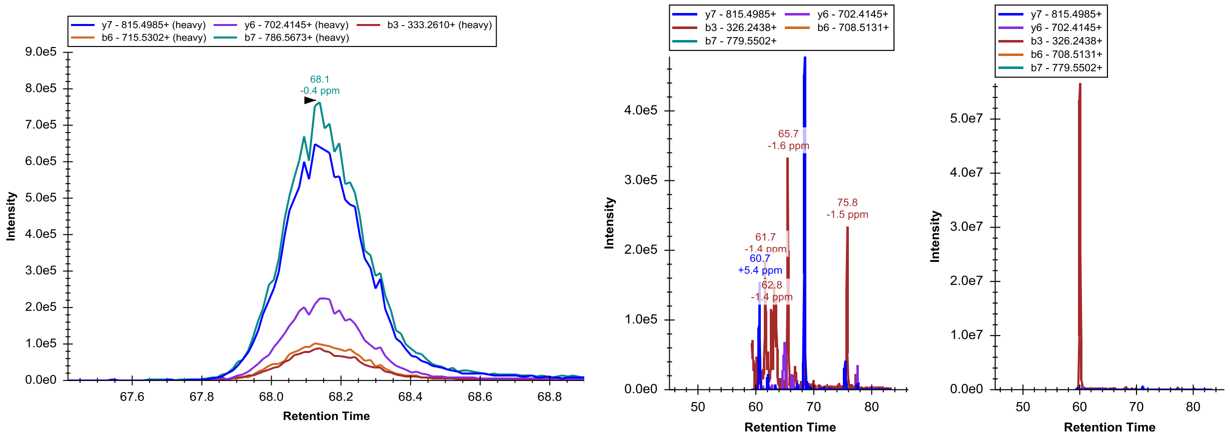

VLLLRLAKT

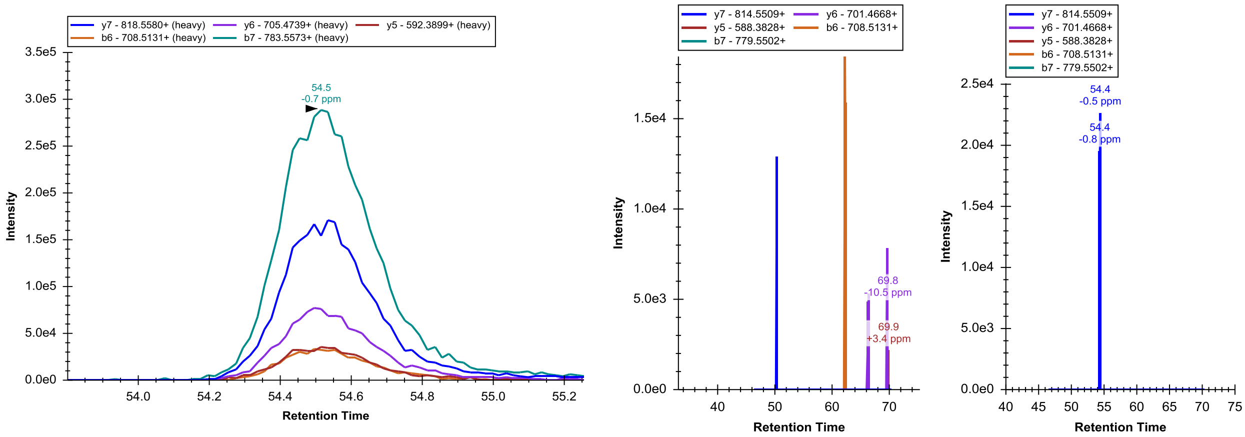

ETYETTLEK

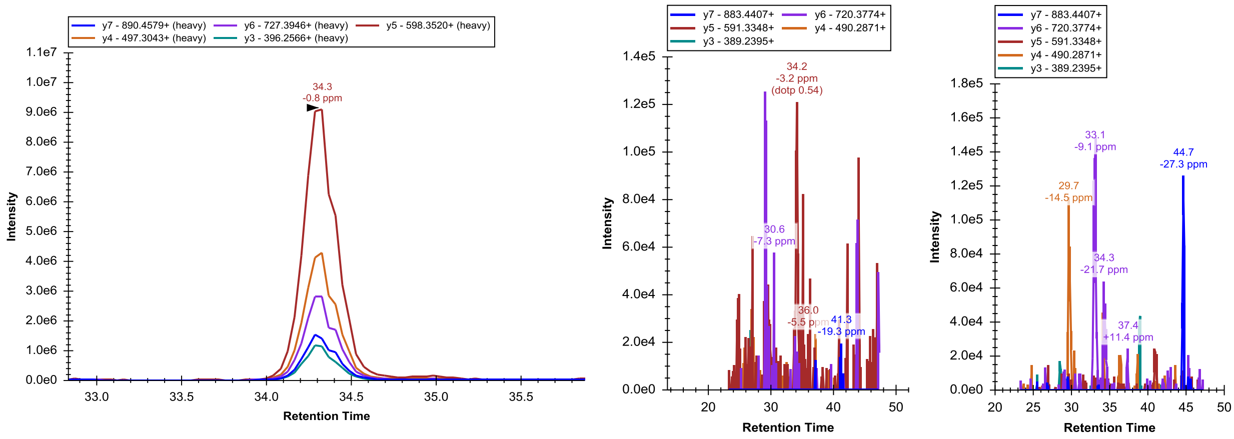

KTYETTLEK

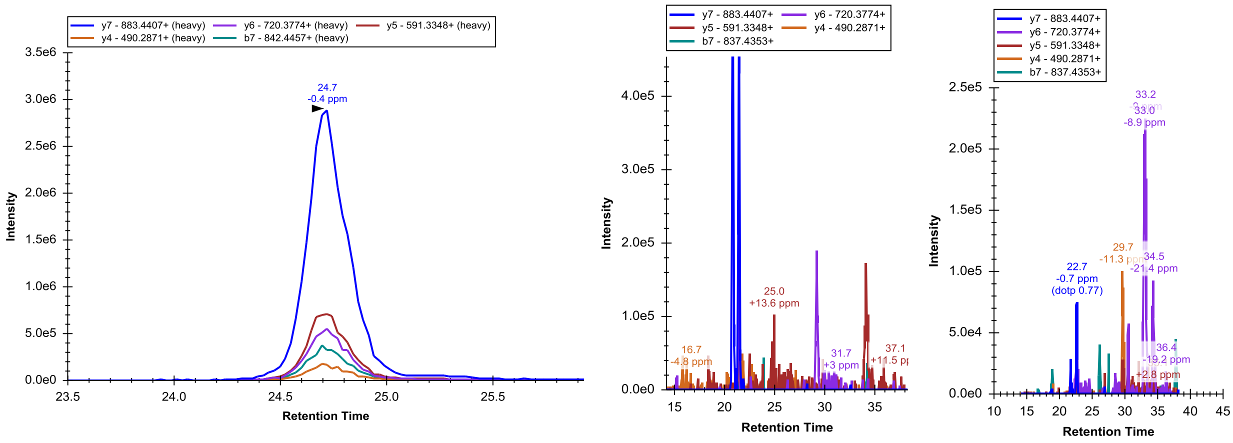

RLAETYETTL

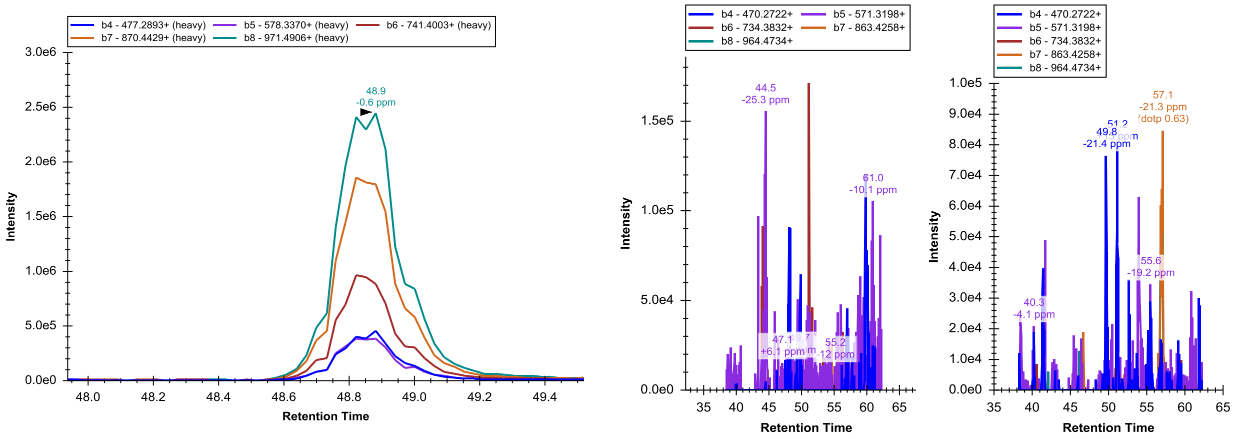

RLAKTYETTL

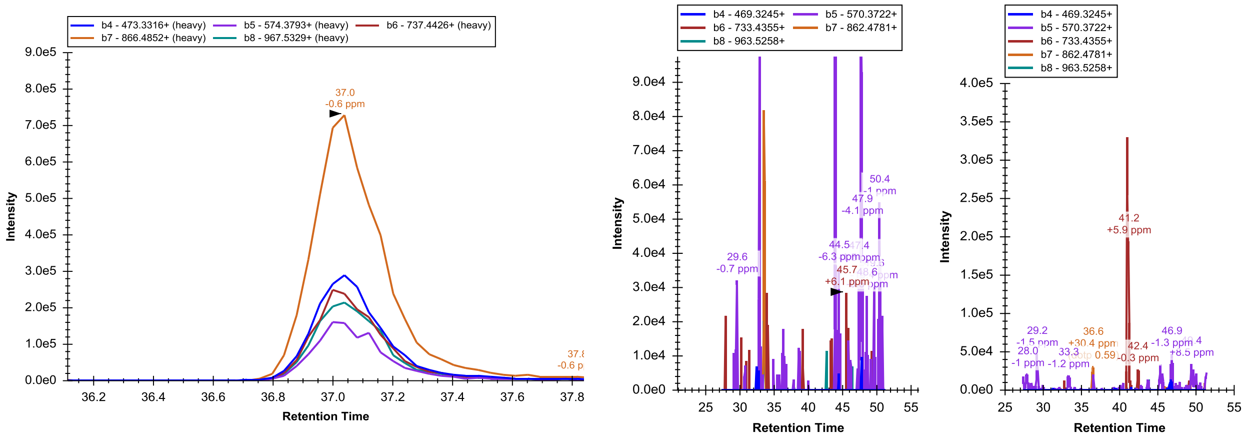

LAETYETTLEK
(2+ and 3+)

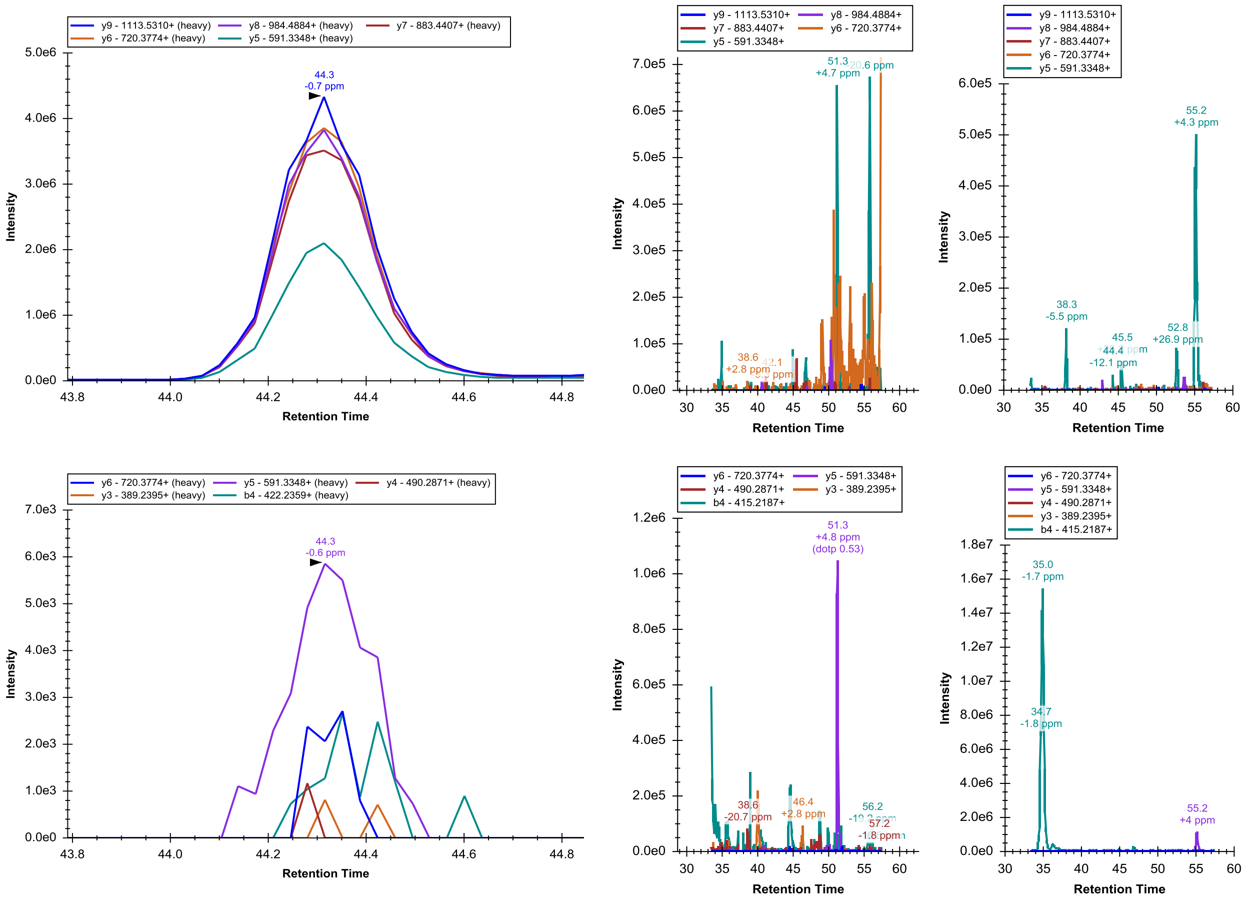

LAKTYETTLEK
(2+ and 3+)

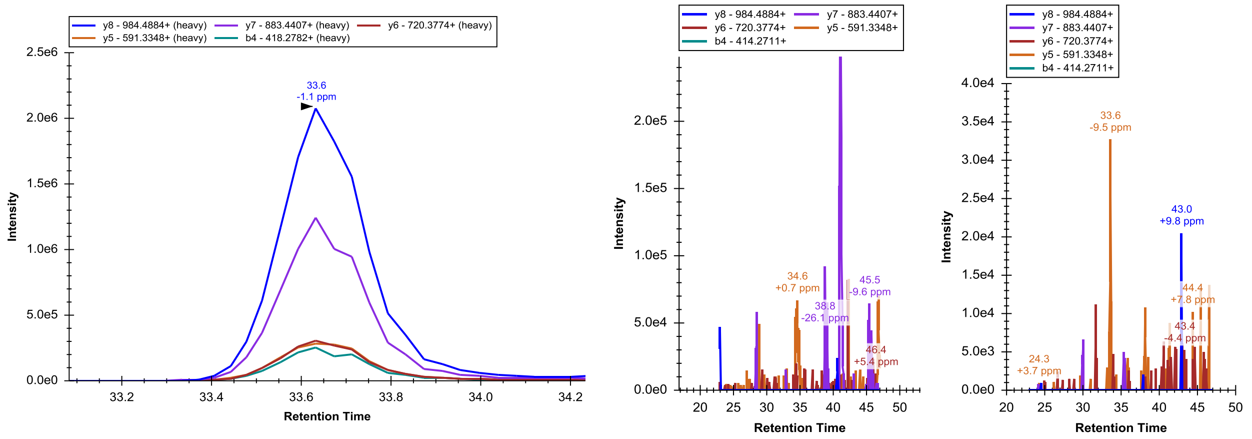

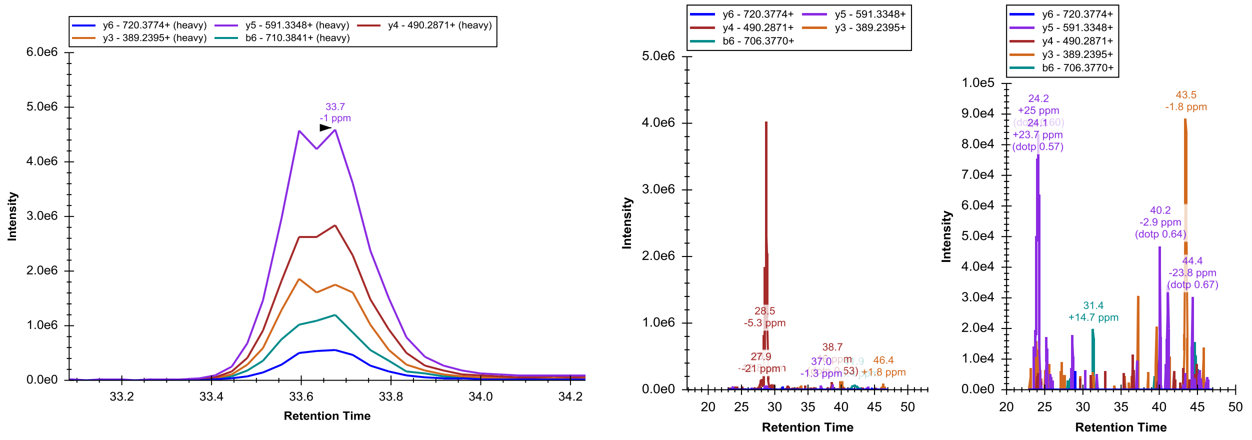

HCC026: RECQL^{H19R} (P46063)

SITSELRAV

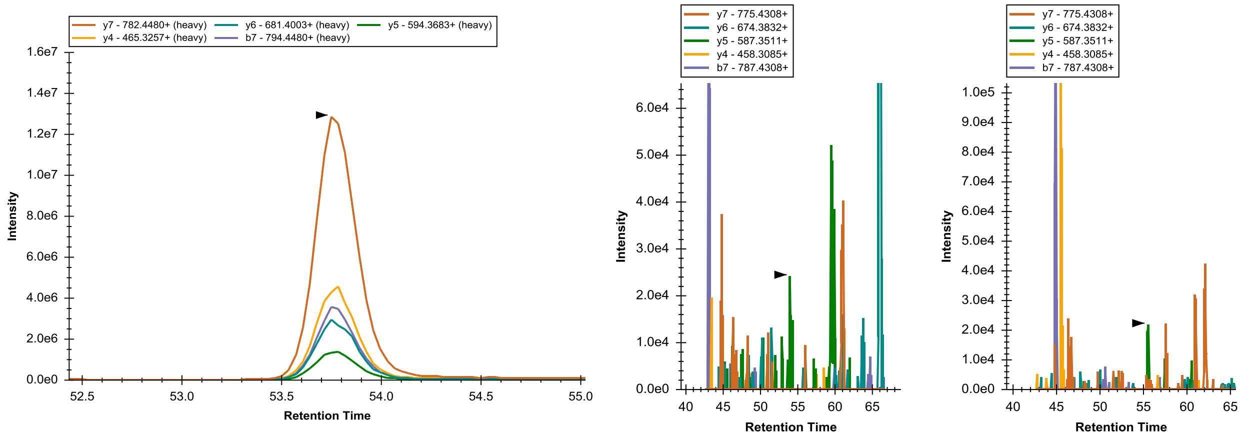

SITSELHAV

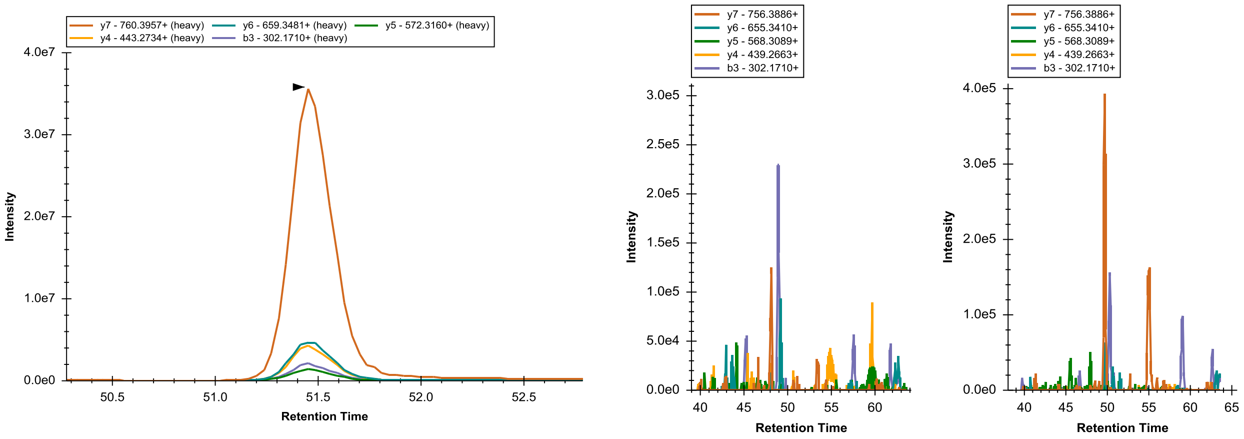

ITSELRAVEI

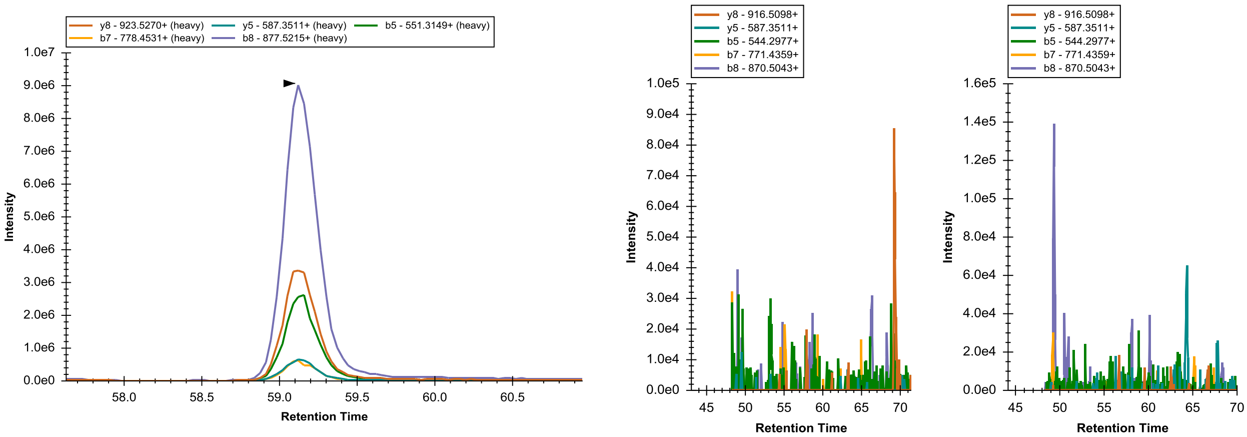

ITSELHAVEI

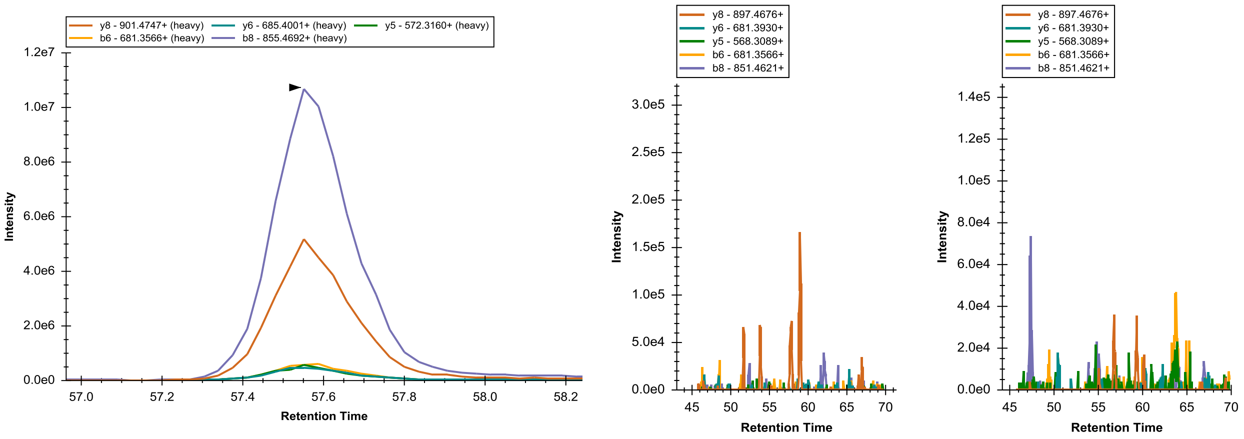

RAVEIQIQEL

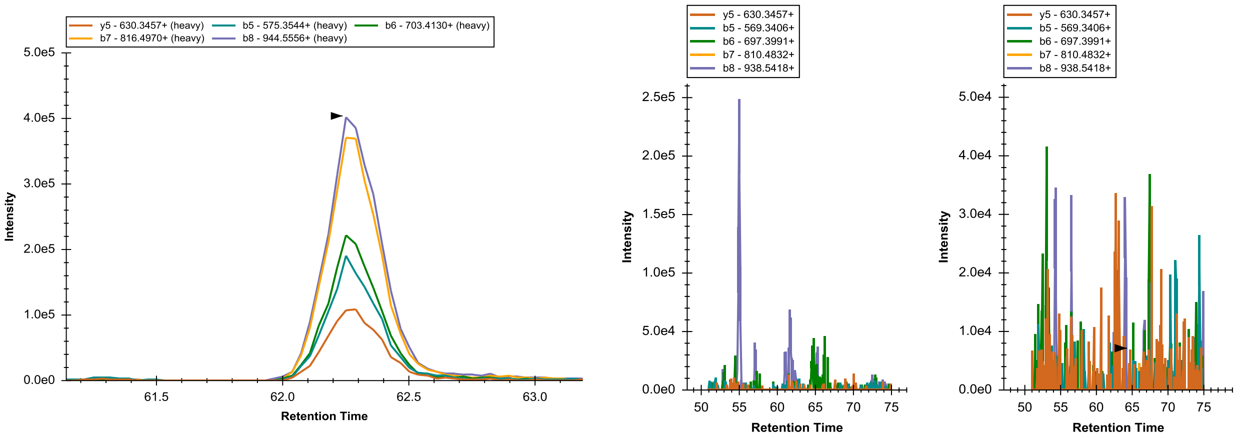

HAVEIQIQEL

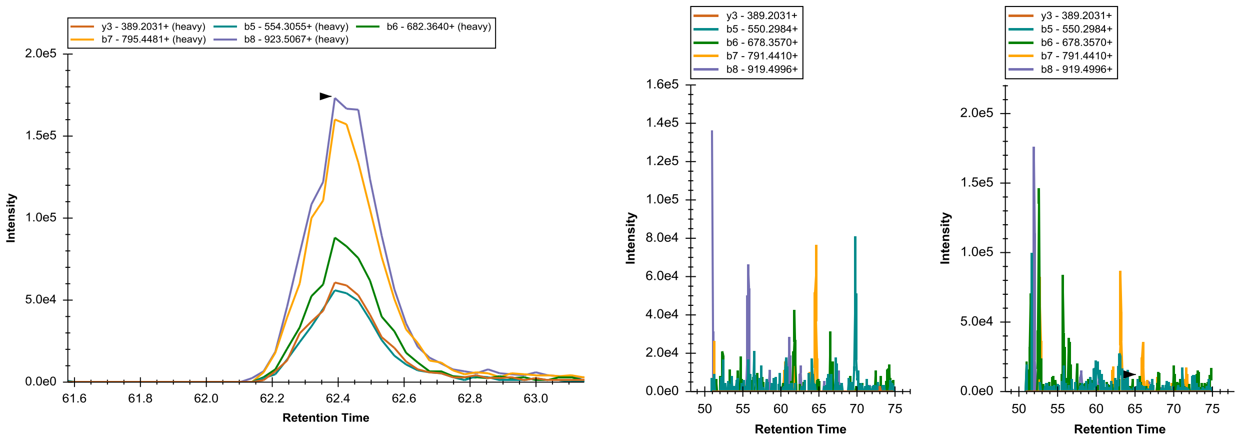
y3 - 389.2031+ (heavy)
b5 - 554.3055+ (heavy)
b6 - 682.3640+ (heavy)
b7 - 795.4481+ (heavy)
b8 - 923.5067+ (heavy)
Intensity
Retention Time
y3 - 389.2031+
b5 - 550.2984+
b6 - 678.3570+
b7 - 791.4410+
b8 - 919.4996+
Intensity
Retention Time
y3 - 389.2031+
b5 - 550.2984+
b6 - 678.3570+
b7 - 791.4410+
b8 - 919.4996+
Intensity
Retention Time

Table S10. *Database matches of peptides observed in Mel dataset.*

Database (DB) matches of peptides represented in the Mel dataset. (ID: identifier; WT^{lig}: wild-type peptide corresponding to PNE; PNE: predicted mutated neoepitope; IEDB: immune epitope database, ttps://www.iedb.org/).

In-house DB	Patient ID	WT^{lig}	PNE	IEDB	Gene
298	Mel5	YIDEQFERY	YIDERFERY	14	SEPT2
47	Mel5	DEEEWLIHY	DEEEWLIYY	1	CABIN1
41	Mel5	KTFPFHFMF	KTFPFYFMF	1	GUCY1A1
30	Mel5	EELEGQSF	KELEGQSF	0	GIMAP7
28	Mel5	DEFQSLVPIL	NEFQSLVPIL	0	MIR127
26	Mel5	LSDHLINQGY	FSDHLINQGY	4	SLC17A9
26	Mel5	VTEKAWNYY	VTKKAWNYY	2	PITPNC1
24	Mel5	RPRETRVIAV	RLRETRVIAV	4	CIITA
23	Mel5	TEQSPTRVL	TEQSSTRVL	1	GUSB
20	Mel5	ETSEQVTRW	ETSKQVTRW	0	GABPA
16	Mel5	TEALNHHNL	TEALNYHNL	0	HLA-DQB2
10	Mel5	FLITSNNQL	FFITSNNQL	0	POLN
8	Mel5	SPMDDGFVSL	SPMNDGFVSL	1	FNTA
7	Mel5	FAYGGHPYPF	FAYGGHPYLF	2	DESI2
7	Mel5	HEKHESENL	HEKHEIENL	0	COL6A2
6	Mel5	LAKERESAL	LAKKRESAL	2	RFXANK
6	Mel5	STFEERSYW	STFEKRSYW	1	HPSE
6	Mel5	RGSAVVGQY	RGDAVVGQY	0	H6PD
5	Mel5	EEAQVMKLL	KEAQVMKLL	0	FGR
5	Mel5	AEKEQELL	AEKKQELL	0	GBP4
5	Mel5	AENAAKALL	AENAAKVLL	0	MCC
4	Mel5	FYFPTPTVL	FYFSTPTVL	0	AP000812.1
4	Mel5	SESSSQVLW	SESLSQVLW	0	AP003721.1

In-house DB	Patient ID	WT^lig	PNE	IEDB	Gene
4	Mel5	HESENLYSI	HEIENLYSI	0	COL6A2
4	Mel5	DEFGRFGSSL	NEFGRFGSSL	0	ITGA5
3	Mel5	NVPDSFNEV	IVPDSFNEV	2	INTS2
3	Mel5	SLSGKIQKL	SLSEKIQKL	0	MGAT4A
3	Mel5	YELDFKAFV	YKLDFKAFV	0	PARP12
3	Mel5	FLIGAGIAAY	FLIEAGIAAY	0	PLPPR3
3	Mel5	FLIGAGIAAY	FLIKAGIAAY	0	PLPPR3
3	Mel5	FLIGAGIAAY	FLIRAGIAAY	0	PLPPR3
3	Mel5	NESTKPPLP	NESTKPPLL	0	USP29
2	Mel5	VQRKVVPTF	VQRKVVSTF	1	HAUS5
2	Mel5	TLKVTSAL	TLKVTSPL	0	BCAM
2	Mel5	AIKKKLTGI	VIKKKLTGI	0	CFL1
2	Mel5	KPFDLVIPF	KPFDLVISF	0	FLNB
2	Mel5	YIDEFQSLV	YINEFQSLV	0	MIR127
2	Mel5	RVRELAVAL	RVRELVVAL	0	MSLN
2	Mel5	ENQPKIQEL	ENQPKIQKL	0	SCN11A
2	Mel5	LSELERVL	LSELKRVL	0	THEG
1	Mel5	HSDPVILNV	HSNPVILNV	1	CEACAM5
1	Mel5	TRFTLPRFL	TQFTLPRFL	1	GAPT
1	Mel5	NEAIRTSTL	NEVIRTSTL	1	GRIA1
1	Mel5	KIRSKVEL	KICSKVEL	0	AC068234.1
1	Mel5	TVGNVLTLL	IVGNVLTLL	0	AC079313.1
1	Mel5	KEFMTPRKL	KEFLTPRKL	0	AL157871.4
1	Mel5	LLENSLSTY	LLEKSLSTY	0	ANGPT1
1	Mel5	HSDPVILNVLY	HSNPVILNVLY	0	CEACAM5
1	Mel5	YIAPGMKVY	YIAPGMKVH	0	EIF2B5
1	Mel5	NEVLWAVV	NEALWAVV	0	GOLPH3

In-house DB	Patient ID	WT^{lig}	PNE	IEDB	Gene
1	Mel5	LEVERPLPM	LEVERPLSM	0	HDGF
1	Mel5	HLYSMQNSY	HLYFMQNSY	0	LMX1A
1	Mel5	KAVSGSIVL	KAVFGSIVL	0	MR1
1	Mel5	YYEIGPVSF	YYEIGPVFF	0	MRAP2
1	Mel5	SSPLRVTSL	SSPLWVTSL	0	MUC16
1	Mel5	NAIGSASVV	NVIGSASVV	0	NPTN
1	Mel5	YEFRVVAV	YKFRVVAV	0	PTPRD
1	Mel5	RQPQFIQGY	RQPQFIQSY	0	ROBO2
1	Mel5	AQVAPVSAL	AQVAPVLAL	0	TM7SF2
1	Mel5	VSKPDVISL	VSKLDVISL	0	ZNF583
0	Mel5	SQHQETPVY	SQHQKTPVY	2	ENTPD1
0	Mel5	SPEGRLYQVEY	SPEGCLYQVEY	2	PSMA4
0	Mel5	YIDEQFERYL	YIDERFERYL	1	SEPT2
0	Mel5	AAKGLPVLKY	AAKELPVLKY	1	AGPAT3
0	Mel5	APRLLQFPEL	APRLLQFSEL	1	EP400
0	Mel5	REVTQDDL	REVTQDNL	1	MED26
0	Mel5	SLIKSVAGV	FLIKSVAGV	1	PIEZO2
0	Mel5	KTKPSPSQF	KTKPSSSQF	1	PNPT1
0	Mel5	EVTDPEKFVY	EVTDSEKFVY	1	TRMT44
0	Mel5	TVGTRNGSI	TVGTRNSSI	1	USH2A
45	Mel8	VEKVVLVSL	VEKLVLVSL	1	C3
41	Mel12	GLISSLNEI	GLISRLNEI	0	SLCO2A1
38	Mel12	APSLHALLL	APRLHALLL	0	GIMAP8
14	Mel12	NRTPSTVTL	NRTSSTVTL	0	KIDINS220
10	Mel12	ATDNMMLEFY	ATNNMMLEFY	0	ARHGAP44
4	Mel12	TEAQRFSSL	TEVQRFSSL	2	ABCG1
2	Mel12	LSAPSLHAL	LSAPRLHAL	0	GIMAP8

In-house DB	Patient ID	WT^{lig}	PNE	IEDB	Gene
1	Mel12	ILDTAGQEEY	ILDTAGREEY	1	CSDE1
1	Mel12	YSDRLVILL	YSDCLVILL	0	DYNC2H1
1	Mel12	GLLKVHYSDRL	GLLKVHYSDCL	0	DYNC2H1
1	Mel12	IFKEHNFSF	IFKEHNYSF	0	FLT3
1	Mel12	APSLHALL	APRLHALL	0	GIMAP8
1	Mel12	STVVGLTVVY	STVVGLIVVY	0	GPR37L1
1	Mel12	FKFDVGTNKY	FTFDVGTNKY	0	INPP5J
1	Mel12	TVALRAAAY	TVALWAAAY	0	LIPE
1	Mel12	LMDSCLHTPMY	LMDSCLHIPMY	0	OR5B2
1	Mel12	ARSKDISYM	ARSKDISYI	0	OR7D4
1	Mel12	DEEGNQFVAY	DEERNQFVAY	0	PAF1
1	Mel12	NPRQQMNGL	NPRQQMNRL	0	TPO
0	Mel12	ILDRLLDGY	ILDKLLDGY	1	GABRA2
0	Mel12	FDLLTEKKTI	FDLLTKKKTI	1	OR4C5
177	Mel15	RPILTIITL	RPILTISTL	8	TP53
141	Mel15	AVILRALSL	AVILRALFL	13	HLA-DPA1
131	Mel15	SPGPSRPGL	FPGPSRPGL	10	ARHGEF1
41	Mel15	FLFPRSIDV	FLFPCSIDV	4	TMEM106B
35	Mel15	RLFPGLAIK	RLFLGLAIK	7	KIF2C
33	Mel15	EISAPSQQR	EILAPSQQR	0	PRPF8
23	Mel15	RTYSLGSALR	RTYSLSSALR	1	VIM
22	Mel15	KATEYIQYM	KASEYIQYM	0	MAX
21	Mel15	FQYAKESYI	FQYAKELYI	1	AL035530.2
14	Mel15	FRFDGVTSM	FRFDGVTFM	3	GBE1
14	Mel15	FQVGDLVQV	FQVDDLVQV	0	MIB1
13	Mel15	FTRAFDQLRM	FTRAFDQLRI	0	TKTL2
11	Mel15	RTYSLGSAL	RTYSLSSAL	1	VIM

In-house DB	Patient ID	WT^{lig}	PNE	IEDB	Gene
11	Mel15	VIENEAGDER	VIENEAGDKR	0	ITIH2
8	Mel15	GLDETIAKL	GLNETIAKL	0	AC005323.2
8	Mel15	LQAETSQQL	LQAETFQQL	0	SNX6
8	Mel15	GRIAFSLKY	GRIAFFLKY	0	SYTL4
7	Mel15	RVYDIPPKFFY	RIYDIPPKFFY	1	AF241726.2
7	Mel15	GRVGIILTL	GRVGIILTV	0	AC000061.1
7	Mel15	RVYDIPPKF	RIYDIPPKF	0	AF241726.2
6	Mel15	GSALRPSTSR	SSALRPSTSR	1	VIM
6	Mel15	GTFSLDAANPK	GTFSLDAASPK	0	MIR4758
5	Mel15	RLFPGLAIKI	RLFLGLAIKI	1	KIF2C
5	Mel15	TSLKFFLNK	TSLKFFFNK	0	GLRX
4	Mel15	ARTKQTARK	ARIKQTARK	0	H3F3C
4	Mel15	DILTPIISL	DILTSIISL	0	MALRD1
4	Mel15	SPVPATPIL	SPVLATPIL	0	TRIM9
3	Mel15	RLYKPILWR	RLYKLILWR	3	NCAPG2
3	Mel15	ALDLGGTNF	ALDLGGTYF	2	HKDC1
3	Mel15	AFHPQPVSR	AFHPQPVSK	2	LRIG1
3	Mel15	KGGPLDGTYRL	KRGPLDGTYRL	0	CA2
3	Mel15	LALDLGGTN	LALDLGGTY	0	HKDC1
3	Mel15	GTVIPSNNEK	GTVILSNNEK	0	SLC12A1
2	Mel15	MPYHIQRTI	MPYHIQLTI	3	CYSLTR1
2	Mel15	DVSVLNSVRR	DVSVLNSVRC	0	CDH5
2	Mel15	AMVDIVRAL	AMMDIVRAL	0	GRM6
2	Mel15	FVVTFPRAY	FVVTFSRAY	0	KDM5A
2	Mel15	ETPKPGTCVKR	ETLKPGTCVKR	0	NUP153
2	Mel15	LRNETNLAY	LRNKTNLAY	0	NYAP2
1	Mel15	KSYFPPKGY	KSYFSPKGY	2	CPEB4

In-house DB	Patient ID	WT^{lig}	PNE	IEDB	Gene
1	Mel15	ASGSIVLFY	ASRSIVLFY	1	LRBA
1	Mel15	ISFITPHAF	ISFITPYAF	1	LRFN5
1	Mel15	DAIKHLDDLK	DAIKHLDNLK	0	AC104389.2
1	Mel15	SPGSGVVTYY	SLGSGVVTYY	0	ACO1
1	Mel15	EVAPTPLDELR	EVALTPLDELR	0	BAD
1	Mel15	EDLSQHPAGY	KDLSQHPAGY	0	CFAP46
1	Mel15	KSRDPRVLR	KSRDPRVFR	0	DDX60
1	Mel15	RIHKPDPWLSK	KIHKPDPWLSK	0	EHHADH-AS1
1	Mel15	YDAVRINQL	YVAVRINQL	0	FERMT1
1	Mel15	EVGDNDLVY	EVGVNDLVY	0	LINGO2
1	Mel15	ERYFSGLIYT	KRYFSGLIYT	0	MYH11
1	Mel15	RAADVRDAM	RAADVQDAM	0	MYO3B
1	Mel15	RRTQRYFME	RRTQRYFMK	0	NEK10
1	Mel15	QAIEFVNQY	QAIKFVNQY	0	NOS2
1	Mel15	AVQGGLDTSK	AVQGSLDTSK	0	PLIN4
1	Mel15	DIGPYQSGR	DISPYQSGR	0	SFRP1
1	Mel15	TILKNTWPK	TILKNTRPK	0	TTC3
1	Mel15	TRTYSLGSAL	TRTYSLSSAL	0	VIM
0	Mel15	YLSGANLNL	YLSRANLNL	12	AC243967.1
0	Mel15	GRKPPLLKK	GRKSPLLKK	3	SCAF11
0	Mel15	REKRTTVVAQL	KEKRTTVVAQL	2	EIF3E
0	Mel15	YLNENPLRA	YLNENLLRA	2	PAK2
0	Mel15	FPHRQPLRY	FLHRQPLRY	1	AC087289.3
0	Mel15	IRYLFQDAF	IRYLFQEAF	1	FASTKD2
0	Mel15	STIEEFSYIRR	SIIEEFSYIRR	1	INO80C
0	Mel15	TRAKLRPSM	MRAKLRPSM	1	IRAK3
0	Mel15	RSGPFGQIFR	RSGSFGQIFR	1	TUBB2B

In-house DB	Patient ID	WT^lig	PNE	IEDB	Gene
0	Mel15	GSYGAWYPLLK	RSYGAWYPLLK	1	UNC13C
102	Mel16	SAFPFLQEY	SAFSFLQEY	1	GSTA4
28	Mel16	LSAFPFLQEY	LSAFSFLQEY	0	GSTA4
2	Mel16	YPETQHVPL	YPKTQHVPL	0	CYP20A1

Table S11. *Database matches of peptides observed in the HCC cohort.*

Database matches of peptides observed in the HCC cohort. (ID: identifier; WT^{lig}: wild-type peptide corresponding to PNE; PNE: predicted mutated neoepitope; IEDB: immune epitope database, https://www.iedb.org/; COSMIC: catalogue of somatic mutations in cancer, https://cancer.sanger.ac.uk/cosmic).

In-house	Patient	WT^{lig}	PNE	IEDB	COSMIC	Gene
11	HCC023	TRAALQKRY	TRAARQKRY	0	0	RHOB
6	HCC023	FSDIHAGELY	FSDIYAGELY	3	0	IDS
1	HCC023	ADVLIKAAL	ADVLIEAAL	0	0	HUWE1
1	HCC023	FLLELGSRV	FLLELSSRV	0	0	PPP1R3D, FAM217B
1	HCC023	REFTQLLL	REFMQLLL	0	0	WDFY4
13	HCC024	RVVGAMQLY	HVVGAMQLY	0	0	CLTC
9	HCC024	FILTHVDQL	FILTHMDQL	1	0	ARHGAP30
59	HCC025	KTYETTLEK	ETYETTLEK	1	0	ALB
7	HCC025	SVQTQPAIKK	SVQTQPAIKN	2	0	BARD1
2	HCC025	EEQIAYAM	AEQIAYAM	0	0	PSMD4
2	HCC025	MLYEEDLQNL	MLYEEDVQNL	0	0	ZHX3
1	HCC025	AKTYETTL	AETYETTL	0	0	ALB
1	HCC025	LEQPPNLEL	LEQPQNLEL	0	0	CD6
1	HCC025	MGLYHGQVL	MVLYHGQVL	0	0	HADHA
0	HCC025	YLDSGIHSGA	YLDAGIHSGA	4	0	CTNNB1
17	HCC026	SITSELHAV	SITSELRAV	1	0	RECQL
16	HCC026	QILEGVYYL	QIFEGVYYL	1	0	STK17B
14	HCC026	NSKRKAETW	NSKRKAETL	3	0	STK38
5	HCC026	LTDIHGNVLQY	ITDIHGNVLQY	8	0	BPNT1

In-house	Patient	WT^{lig}	PNE	IEDB	COSMIC	Gene
3	HCC026	KQILEGVYYL	KQIFEGVYYL	0	0	STK17B
2	HCC026	VRDGATLIL	VRDGTTLIL	0	0	PLXNB2
1	HCC026	RLATSYIAY	CLATSYIAY	0	0	HAND1
1	HCC026	AFKGLKSLEY	AFKGLKSLKY	0	0	LUM
1	HCC026	AAFKGLKSLEY	AAFKGLKSLKY	0	0	LUM
0	HCC026	YLDSGIHSGA	YLDSGIHYGA	4	0	CTNNB1
	HCC026	RLIKQILEGV	RLIKQIFEGV	1	0	STK17B
119	HCC027	YITERIIAV	YITERIVAV	3	0	TNS3
54	HCC027	TERIIAVSF	TERIVAVSF	1	0	TNS3
7	HCC027	ATMEDTVEY	ATMEDAVEY	3	0	ECD
1	HCC027	TEVPDFNKMY	TEVPDFSKMY	0	0	TXNL4A
33	HCC028	LPAHIPYQEL	LPTHIPYQEL	3	0	SPECC1L-ADORA2A
5	HCC028	RPRPPTLL	RPGPPTLL	2	0	EIF3B
5	HCC028	GFYPGSIEV	GFYPGSVEV	2	0	HLA-DRB5
3	HCC028	AKTDVLISV	AKTEVLISV	0	0	TEX15
1	HCC028	FYPGSIEVRW	FYPGSVEVRW	0	0	HLA-DRB5
1	HCC028	THPDVQQKL	SHPDVQQKL	0	0	RP11-757A13.1
0	HCC028	YLDSGIHSGA	YLYSGIHSGA	4	0	CTNNB1
0	HCC028	RPRPPTLLSQ	RPGPPTLLSQ	1	0	EIF3B
0	HCC028	YLPAHIPYQEL	YLPTHIPYQEL	1	0	SPECC1L-ADORA2A
18	HCC030	SLYGLGGSK	SLYDLGGSK	0	0	KRT6C
3	HCC030	QLLPNVTTV	QLLPNITTV	0	0	GGT1
1	HCC030	FIADAHEKISV	FIADAHEKIAV	0	0	C10orf76
1	HCC030	SLYGLGGSKR	SLYDLGGSKR	0	0	KRT6C
0	HCC030	YLDSGIHSGA	YLDFGIHSGA	4	0	CTNNB1

In-house	Patient	WT^{lig}	PNE	IEDB	COSMIC	Gene
0	HCC030	LRLTGKIDF	LRLTGKIDL	1	0	APOB
0	HCC030	ILKERGGSK	ILKEHGGSK	1	0	TOPBP1
16	HCC035	GLAVFQAFL	GLAVFQALL	0	0	RGS3
1	HCC035	ALRVDIEAL	VLRVDIEAL	0	0	SIGLEC1
6	HCC036	NRAATLMML	NRAATLIML	2	0	DNAJC7
5	HCC036	DYPDYKYRPR	DYPDYNYRPR	0	0	SOX11
3	HCC036	MADYPDYKY	MADYPDYNY	2	0	SOX11
2	HCC036	FAYDYDLTHI	FAYDYDLIHI	0	0	RP11-77K12.7, TMEM231, RP11-77K12.8
1	HCC036	RGPPGVAGI	LGPPGVAGI	1	0	COL9A1
1	HCC036	LVVGDIAEL	VVVGDIAEL	0	0	AMT
39	HCC038	AQILQILTV	AQILQILTI	1	0	GCN1L1
7	HCC038	IPAGERPTI	IPAGEPPTI	3	0	ZNF48
2	HCC038	NPYIGGLGI	NPNIGGLGI	0	0	BIRC6
2	HCC038	NLIPIFEEL	NLIPIFEDL	0	0	CTDSP2
2	HCC038	NLIPIFEEL	NLIPIFEEM	0	0	CTDSP2
1	HCC038	RPLFPGTSTL	RPLFPGTSNL	0	0	MAPK15
1	HCC038	NYLTPIKYL	SYLTPIKYL	0	0	PRAMEF1
3	HCC040	GTVFIIQGLR	GTVLIIQGLR	0	0	MIR3135B
1	HCC040	TTAPSLSGK	TTAAPLSGK	6	COSM17661, COSM5663	CTNNB1
1	HCC040	TTAPSLSGK	TTAASLSGK	6	COSM17661	CTNNB1
1	HCC040	TTAPSLSGK	TTAPPLSGK	6	COSM5663	CTNNB1
1	HCC040	TVFIIQGLR	TVLIIQGLR		0	MIR3135B

In-house	Patient	WT^{lig}	PNE	IEDB	COSMIC	Gene
37	HCC041	RSSVILLTY	RSSFILLTY	1	0	SLC22A18
8	HCC041	GRSSVILLTY	GRSSFILLTY	5	0	SLC22A18
1	HCC041	LFNRISLL	LFNRISHL	0	0	CTD-3018O17.3
1	HCC041	LPRHSFGRNAL	WPRHSFGRNAL	0	0	GABRB2
0	HCC041	LEDRRAAVDTY	LEDRGAAVDTY	1	0	HLA-DRB5
0	HCC041	LEDRRAAVDTY	LEDSGAAVDTY	1	0	HLA-DRB5
0	HCC041	LEDRRAAVDTY	LEDSRAAVDTY	1	0	HLA-DRB5
5	HCC042	VTEEEGSANMY	VSEEEGSANMY	2	0	RP11-210N13.1
1	HCC042	YLKDFEIVK	YLKEFEIVK	0	0	ANKRD36C
1	HCC042	VLRVYIGSF	VLRIYIGSF	0	0	CTD-2571L23.8
0	HCC042	SLLNLHLHAL	SLLNLHLHTL	1	0	TRBV3-1
135	HCC043	RLASYLDKV	RLASYLDKL	2	0	KRT19
34	HCC043	AEEWYKSKF	AEEWCKSKF	2	0	VIM
21	HCC043	SRIALKSGY	SSIALKSGY	8	0	FRG1
18	HCC043	DLEPGTMDSV	YLEPGTMDSV	0	0	RP11-683L23.1
13	HCC043	SYLDKVRAL	SYLDKLRAL	0	0	KRT19
12	HCC043	WYKSKFADL	WCKSKFADL	1	0	VIM
7	HCC043	REFGAGPLF	CEFGAGPLF	5	0	SF3B1
7	HCC043	VPRAVLVDL	VPRAVLVYL	1	0	RP11-683L23.1
7	HCC043	YLDKVRAL	YLDKLRAL	0	0	KRT19
5	HCC043	KIAEIEAEM	KIAEMEAEM	0	0	DRG1
3	HCC043	LVDLEPGTM	LVYLEPGTM	1	0	RP11-683L23.1
1	HCC043	ASYLDKVRAL	ASYLDKLRAL	1	0	KRT19
1	HCC043	SRKEREAEL	SRKKREAEL	0	0	PABPC3
1	HCC043	LTLPSALHF	LMLPSALHF	0	0	PCNXL3

In-house	Patient	WT^{lig}	PNE	IEDB	COSMIC	Gene
1	HCC043	YKSKFADL	CKSKFADL	0	0	VIM
18	HCC045	NLDIERPTY	NLDIESPTY	5	0	MZT2B
12	HCC045	KTVTAMDVVY	KTVTAMEVVY	6	0	HIST1H3I
2	HCC045	QENYAGIFHF	QENYVGIFHF	0	0	CAPN8
2	HCC045	ISDDTTHPISY	ISDDTTHTISY	0	0	GGT1
2	HCC045	ISDDTTHPISY	ISDNTTHPISY	0	0	GGT1
2	HCC045	ISDDTTHPISY	ISDNTTHTISY	0	0	GGT1
2	HCC045	DVVYALKRQG	EVVYALKRQG	0	0	HIST1H3I

Table S12. *Cancer-testis antigens (CTA) characterized in HCC.*

Cancer-testis antigens (CTA) with evidence on shotgun proteome level.

Gene	Patient ID
**ATAD2**	HCC025
**LDHC**	HCC034, HCC026, HCC027, HCC024, HCC025, HCC023, HCC036
**NR6A1**	HCC027
**POTEB**	HCC023
**POTEC**	HCC023
**POTEE**	HCC034, HCC026, HCC027, HCC024, HCC025 HCC023, HCC036
**POTEG**	HCC026, HCC023
**POTEH**	HCC026, HCC023
**RBM46**	HCC026, HCC024, HCC025
**TEX15**	HCC026, HCC027, HCC024, HCC023

Table S13. *Identified pathways with differentially expressed genes.*

Pathway ID	Description	Gene ratio	q-value
hsa00830	Retinol metabolism	14/115	1.64e-10
hsa00982	Drug metabolism - cytochrome P450	12/115	6.14e-08
hsa00140	Steroid hormone biosynthesis	10/115	7.65e-07
hsa00980	Metabolism of xenobiotics by cytochrome P450	11/115	7.65e-07
hsa05204	Chemical carcinogenesis	11/115	1.39e-06
hsa00120	Primary bile acid biosynthesis	5/115	0.00012
hsa00590	Arachidonic acid metabolism	7/115	0.0011
hsa00591	Linoleic acid metabolism	5/115	0.0015
hsa00760	Nicotinate and nicotinamide metabolism	5/115	0.0016
hsa00983	Drug metabolism - other enzymes	7/115	0.0034
hsa00380	Tryptophan metabolism	5/115	0.0053
hsa04976	Bile secretion	6/115	0.011
hsa03320	PPAR signaling pathway	6/115	0.012
hsa04979	Cholesterol metabolism	5/115	0.012
