# Supplementary material for: Multi-omics discovery of exome-derived neoantigens in hepatocellular carcinoma
Source: Genome Med. 2019 Apr 30;11:28. doi: 10.1186/s13073-019-0636-8 (PMC6492406; doi:10.1186/s13073-019-0636-8)
Supplement: Supplementary file 2 — Extended Materials and Methods. Comprehensive Materials and Methods section with detailed descriptions of experiments and employed materials and tools. (PDF 516 kb) [file 13073_2019_636_MOESM2_ESM.pdf]

Multi-omics discovery of exome-derived neoantigens in hepatocellular carcinoma

Markus W. Löffler[1,2,3,4,#], Christopher Mohr[5,6,#], Leon Bichmann[2,7,8], Lena Katharina Freudenmann[2,3], Mathias Walzer[2,7,8,9], Christopher M. Schroeder[10], Nico Trautwein[2], Franz J. Hilke[10], Raphael S. Zinser[2], Lena Mühlenbruch[2], Daniel J. Kowalewski[2,11], Heiko Schuster[2,11], Marc Sturm[10], Jakob Matthes[10], Olaf Riess[10, 12], Stefan Czemmel[6], Sven Nahnsen[6], Ingmar Königsrainer[1], Karolin Thiel[1], Silvio Nadalin[1], Stefan Beckert[1,13], Hans Bösmüller[14], Falko Fend[14], Ana Velic[15], Boris Maček[15], Sebastian P. Haen[2,3,16], Luigi Buonaguro[17], Oliver Kohlbacher[3,5,6,7,8,12,18], Stefan Stevanović[2,3], Alfred Königsrainer[1,3], HEPAVAC Consortium and Hans-Georg Rammensee[2,3]

[1] University Hospital Tübingen, Department of General, Visceral and Transplant Surgery, Hoppe-Seyler-Str. 3, D-72076 Tübingen, Germany
[2] University of Tübingen, Interfaculty Institute for Cell Biology, Department of Immunology, Auf der Morgenstelle 15, D-72076 Tübingen, Germany
[3] German Cancer Consortium (DKTK) and German Cancer Research Center (DKFZ) Partner Site Tübingen, Tübingen, Germany
[4] University Hospital Tübingen, Department of Clinical Pharmacology, Auf der Morgenstelle 8, D-72076 Tübingen, Germany
[5] University Hospital Tübingen, Institute for Translational Bioinformatics, Tübingen, Germany
[6] University of Tübingen, Quantitative Biology Center (QBiC), Auf der Morgenstelle 10, D-72076 Tübingen, Germany
[7] University of Tübingen, Center for Bioinformatics, Sand 14, D-72076 Tübingen, Germany
[8] Department of Computer Science, Applied Bioinformatics, Sand 14, D-72076 Tübingen, Germany
[9] Current address: European Molecular Biology Laboratory, European Bioinformatics Institute (EMBL-EBI), Wellcome Trust Genome Campus, Hinxton, Cambridgeshire, CB10 1SD, United Kingdom
[10] University Hospital Tübingen, Institute of Medical Genetics and Applied Genomics, Calwerstr. 7, D-72076 Tübingen, Germany
[11] Current address: Immatics Biotechnologies GmbH, Paul-Ehrlich-Str. 15, D-72076 Tübingen, Germany
[12] NGS Competence Center Tübingen (NCCT), University of Tübingen, Tübingen, Germany
[13] Current address: Department of General and Visceral Surgery, Schwarzwald-Baar Hospital, Klinikstr. 11, D-78052 Villingen-Schwenningen, Germany.
[14] University Hospital Tübingen, Institute of Pathology and Neuropathology, Liebermeisterstr. 8, D-72076 Tübingen, Germany
[15] University of Tübingen, Interfaculty Institute for Cell Biology, Proteome Center Tübingen (PCT), Auf der Morgenstelle 15, D-72076 Tübingen, Germany
[16] University of Tübingen, Internal Medicine, Department for Oncology, Hematology, Immunology, Rheumatology and Pulmonology, Otfried-Müller-Str. 10, D-72076 Tübingen, Germany
[17] Cancer Immunoregulation Unit, Istituto Nazionale per lo Studio e la Cura dei Tumori, "Fondazione Pascale" - IRCCS, 80131 Naples, Italy
[18] Max Planck Institute for Developmental Biology, Biomolecular Interactions, Spemannstr. 35, D-72076 Tübingen, Germany

Corresponding authors: **Markus W. Löffler, MD**, University of Tübingen, Interfaculty Institute for Cell Biology, Department of Immunology, Auf der Morgenstelle 15, D-72076 Tübingen, Germany, E-mail: **markus.loeffler@uni-tuebingen.de**; and **Christopher Mohr, MSc** (Bioinformatics), University of Tübingen, Quantitative Biology Center (QBiC), Auf der Morgenstelle 10, D-72076 Tübingen, Germany, E-mail: **christopher.mohr@uni-tuebingen.de**

Additional File 2.

Extended Materials and Methods

Ethics approval and informed consent

This study was conducted in accordance with the Declaration of Helsinki and approved by the local institutional review board at the University Hospital of Tübingen, Germany. All participants provided written informed consent before study inclusion.

Clinical specimens

Clinical specimens from patients (n=16) undergoing liver resection for hepatocellular carcinomas (HCC) encompassing both non-malignant and malignant liver tissue as well as peripheral blood were obtained directly after surgery and cryopreserved (for patients´ tumor characteristics see **Additional File 1: Table S1**). HCC diagnosis and predominant tumor fraction within samples were histologically confirmed by an expert pathologist. All included patients were negative for chronic viral hepatitis (hepatitis B and/or C) and without systemic pretreatment for their malignancy.

Next-Generation Sequencing

Extraction of DNA/RNA from fresh frozen tissue and PBMCs was performed using the AllPrep DNA/RNA Kit (Qiagen) from fresh frozen tissue and PBMCs, respectively (a sample and analysis overview is provided in **Additional File 1: Table S2**).

For whole exome sequencing (WES) samples (HCC023-027, HCC034, and HCC036) were prepared using the SureSelectXT Human All Exon v5 or v6 Kit (Agilent, Waldbronn, Germany). For whole transcriptome sequencing samples were prepared with the TruSeq Stranded mRNA Kit (Illumina, Eindhoven, Netherlands). Paired-end sequencing was performed with the HiSeq 2500 or NextSeq500 System (Illumina).

For WES, DNA libraries were prepared for samples (HCC028, HCC030, HCC035, and HCC038-045) with SureSelect^{XT} Human All Exon v6 Kit (Agilent, Waldbronn, Germany) and sequenced in paired-end mode on a HiSeq 4000 System (Illumina, Eindhoven, Netherlands). RNA library preparation was performed using the SMARTer Stranded Total RNA-Seq Kit v2 – Pico Input Mammalian (Clontech,

Saint-Germain-en-Laye, France) and sequenced on a HiSeq 4000 System (Illumina, Eindhoven, Netherlands).

HLA typing

Typing at four-digit resolution using WES data was performed by OptiType (1) for HLA class I alleles as previously described (2) and confirmed in selected cases by molecular HLA typing (using clinically validated LUMINEX and sequence-based typing) during clinical routines (see **Additional File 1: Table S3**).

Isolation of naturally presented HLA ligands from tissues for HLA ligandomics

HLA class I-peptide complexes were isolated from HCC and corresponding (non-malignant) liver tissue samples by immunoaffinity purification as described previously (3), using the pan-HLA class I-specific monoclonal antibody W6/32 (4) (produced in-house at the Department of Immunology) and eluted using 0.2 % trifluoroacetic acid.

Mass spectrometric analysis of naturally presented HLA-bound peptides

Peptide extracts were separated by UHPLC (UltiMate 3000 RSLCnano System, Dionex) at a flow rate of 175 nl/min using a 50 µm × 25 cm C18 column (PepMap RSLC, 2 µm particle size, Thermo Fisher) and a linear gradient ranging from 3 to 40 % solvent B over the course of 90 minutes (Solvent A: 0.15 % formic acid; Solvent B: 80 % ACN), as described previously (3, 5). Eluting peptides were analyzed in an online coupled LTQ Orbitrap XL mass spectrometer (Thermo Fisher) operated in automated data-dependent acquisition (DDA) mode. In the Orbitrap, survey scans of peptides with 400-650 m/z as well as 2+ and 3+ as permitted charge states were recorded at a resolution of 60,000 with subsequent selection of the five most abundant precursor ions for collision-induced dissociation (CID). The normalized collision energy was set to 35, activation time to 30 ms and the isolation width to 2.0 m/z. MS/MS spectra were acquired in the linear ion trap (LTQ) and corresponding precursor ions were dynamically excluded for 3 s after fragmentation. Each sample was acquired once or in multiple technical replicates.

To enhance sensitivity of neoantigenic peptide identification, we additionally performed selected ion monitoring (SIM; LTQ Orbitrap XL) and parallel reaction monitoring (PRM) targeted tandem MS (tMS2) (Orbitrap Fusion Lumos, Thermo Fisher) for selected samples.

Heavy isotope-labeled synthetic peptides for the SIM approach (**Additional File 1: Table S7**) were purchased from Thermo Fisher. Synthetic peptide retention times (RT) were assessed in an HLA class I peptide matrix eluted from JY cells. Subsequently, these were used to create a scheduled SIM

method triggering fragmentation of PNEs (2+ precursor ions) independent of their relative abundance in patient peptide extracts. Due to the number of PNEs, three SIM measurements were scheduled and thus several measurements per tumor sample were necessary. SIM scans of HCC025 and HCC026 were performed with the same UHPLC settings as top 5 CID measurements, whereas HCC027 SIM acquisition was performed using a 50 µm × 50 cm C18 column (PepMap RSLC, 2 µm particle size, Thermo Fisher) and a linear gradient ranging from 3 to 40 % solvent B over the course of 140 minutes. The normalized collision energy was set to 35, activation time to 30 ms and the isolation width to 1.5-2.0 m/z.

Heavy isotope-labeled synthetic peptides for PRM tMS2 (**Additional File 1: Table S8**) were manufactured in-house by solid-phase peptide synthesis at a purity >60 %. PRM tMS2 methods targeting the heavy isotope-labeled synthetic peptide or its natural counterpart (2+ and 3+ precursor ions) were created using Skyline v4.1 (6, 7). The retention time (RT) as well as the amount of each synthetic peptide necessary for reliable detection was assessed by titration in an HLA class I peptide matrix eluted from JY cells. Synthetic peptide purity as determined by high performance liquid chromatography (HPLC) and 75 % peptide content (reference values of nitrogen determination: 20 % TFA and 5-10 % H_2O) were considered for weighed-in amounts. Peptides dissolved in 10 % DMSO were spiked at 4 – 20 fmol/µl and 5 µl were injected for PRM tMS2 measurements on an Orbitrap Fusion Lumos. Raw files of these titration measurements were processed with Proteome Discoverer v1.4 (Thermo Fisher) using the SEQUEST HT search engine (8). Based on synthetic peptide RTs±12 min, PRM tMS2 data acquisition of tumor and autologous non-malignant liver samples was scheduled. Peptide extracts were separated by UHPLC (UltiMate 3000 RSLCnano System, Dionex) at a flow rate of 300 nl/min using a 50 µm × 25 cm C18 column (PepMap RSLC, 2 µm particle size, Thermo Fisher) and a linear gradient ranging from 3 to 40 % solvent B over the course of 90 minutes (Solvent A: 0.15 % formic acid; Solvent B: 80 % ACN). Eluting peptides were analyzed in an online coupled Orbitrap Fusion Lumos mass spectrometer (Thermo Fisher). In the Orbitrap, survey scans of precursor ions (HCC025: 320-670 m/z, HCC026: 300-650 m/z; 2+ and 3+ as permitted charge states) were recorded at a resolution of 120,000 with subsequent selection for collision-induced dissociation (CID). The normalized collision energy was set to 35 and the isolation width to 1.4 m/z. At a resolution of 60,000, MS/MS spectra were acquired in the Orbitrap. In addition to PRM tMS2 measurements, we performed one top n run in DDA mode (HCC025: 320-670 m/z, HCC026: 300-650 m/z) per sample. In the Orbitrap, survey scans of precursor ions (HCC025: 320-670 m/z, HCC026: 300-650 m/z; 2+ and 3+ as permitted charge states) were recorded at a resolution of 120,000 with subsequent selection for CID. The normalized collision energy was set to 35 and the isolation width to 1.4 m/z. At a resolution of 30,000, MS/MS spectra were acquired in the Orbitrap and corresponding precursor ions were dynamically excluded for 7 s after fragmentation. Synthetic

peptides in an HLA class I peptide matrix were back-to-back eluted and acquired in scheduled PRM tMS2 (4 fmol/µl) or in top n DDA mode (10 fmol/µl), respectively. DDA measurements of synthetic peptides (10 fmol/µl), were processed with Proteome Discoverer v1.4 (Thermo Fisher) using the SEQUEST HT search engine (8) and served as spectral library for analysis of scheduled PRM tMS2 data in Skyline.

Protein in-gel digestion for shotgun protein identification

Eluted protein samples were purified by SDS-PAGE. Coomassie-stained gel pieces were digested using trypsin. Extracted peptides were desalted using C18 Stage tips and subjected to LC-MS/MS analysis.

Shotgun protein tandem mass spectrometry

Liquid chromatography tandem mass spectrometry (LC-MS/MS) analyses were performed on an EasyLC nano-HPLC (Proxeon Biosystems, Roskilde, Denmark) coupled to an LTQ Orbitrap Elite (Thermo Fisher).

Peptide mixtures were separated on a 15 cm fused silica emitter of 75 µm inner diameter (Proxeon), in-house packed with reversed-phase ReproSil-Pur C18-AQ 3 µm resin (Dr. Maisch GmbH, Ammerbuch, Germany). Peptides were injected with solvent A (0.5 % acetic acid) at a flow rate of 500 nl/min and separated at 200 nl/min. Separation was performed using a linear 130 min gradient of 5-33 % solvent B (80 % ACN in 0.5 % acetic acid). Each of four samples was run as one technical replicate. LTQ Orbitrap Elite was operated in the positive ion mode. Precursor ions were acquired in the mass range from 300 to 2,000 m/z followed by MS/MS spectra acquisition of the 20 most intense precursor ions. Higher-energy CID (HCD) MS/MS spectra were acquired with a resolution of 15,000 and a target value of 40,000. The normalized collision energy was set to 35, activation time to 0.1 ms and the first mass to 120 Th. Fragmented masses were excluded for 60 s after MS/MS. The target values were 1E6 charges for the MS scans in the Orbitrap and 5,000 charges for the MS/MS scans with a maximum fill time of 100 ms and 150 ms, respectively.

Proteomic data analysis

MS data were processed with MaxQuant software suite v.1.5.2.8 (9). Database search was performed using the Andromeda search engine (10) integrated into the MaxQuant framework. The human reference database was obtained from UniProt (taxonomy ID 9606, containing 91,646 protein entries and 285 commonly occurring laboratory contaminants) and concatenated with the patient-specific mutanome. Endoprotease trypsin was fixed as protease with a maximum of two missed cleavages.

Oxidation of methionines and N-terminal acetylation were specified as variable modifications, whereas carbamidomethylation of cysteines was defined as a fixed modification. Initial maximum allowed mass tolerance was set to six ppm. Re-quantify was enabled. An FDR of 1 % was applied at peptide and protein level.

Bioinformatics

Data management and bioinformatic analysis was performed through the qPortal instance at the Quantitative Biology Center, Tübingen, if not stated otherwise (11).

Variant calling

Generated reads were processed using the megSAP pipeline (https://github.com/imgag/megSAP) and the ngs-bits package (https://github.com/imgag/ngs-bits) by the Department of Medical Genetics and Applied Genomics (Tübingen, Germany) [HCC023-HCC027/HCC034/HCC036]. Adapter trimming was performed with SeqPurge (12). Reads were mapped against the Genome Reference Consortium Human Build 37 (GRCh37) using BWA-mem (13). Samblaster (14) was used for duplicate annotation. Local realignment of reads in target regions was done with ABRA (15). Overlapping reads were trimmed with an in-house tool for reduction of false-positive variants with very low allele frequencies. Somatic variant calling was performed using Strelka and Strelka2 (16, 17). Derived variants were annotated with SnpEff/SnpSift (18, 19), vcflib (https://github.com/ekg/vcflib), and dbNFSP (20). High-confidence variants were obtained using custom filter criteria and further annotated with in-house variant frequencies, tumor RNA depth and allele frequencies. RNA reads were preprocessed to remove adaptor sequences in the same way and then mapped with STAR (21) to the same reference genome. Otherwise, sequenced reads were demultiplexed with Illumina bcl2fastq 2.19. and Skewer 0.2.2 (22) was used for adapter trimming, followed by read mapping with an in-house version of BWA-mem (13) v0.72 against an in-house version of hg19. Local realignment of reads in target regions was done with ABRA (15) and duplicate reads were discarded using SAMtools v0.1.18 (23). Somatic variants, called with a proprietary software (CeGaT GmbH, Tübingen, Germany), were filtered for a minimal coverage of 30x in tumor and non-malignant tissue and an allele frequency greater than 0.05 in tumor tissue and three-fold less in non-malignant tissue. In case of HCC028, HCC030, HCC035, and HCC038-HCC045, somatic mutations were annotated using SnpEff 4.1k (19).

Gene expression analysis

Gene expression values were calculated as fragments per kilobase of exon per million reads mapped (FPKM) of the corresponding transcripts and RNA tumor sequencing depth at the corresponding

variant position. Mapping of RNA reads was done using TopHat 2 (v2.0.12) (24). Adapters were removed beforehand with CutAdapt (--discard-trimmed) based on FastQC results (v0.10). Counts for mapped RNA reads were calculated using HTSeq (0.6.1p2) (25). FPKM values were calculated as follows:

$$FPKM = \frac{10^9 \times C}{N \times L}$$

where, *L* is the exon length in base pairs for the corresponding gene, *C* is the number of reads that mapped to a gene (number of counts from HTSeq run), and *N* is the total number of unique mapped reads in the sample.

Peptide prediction

Peptides of 8-11 amino acids length were constructed by sliding a shifting window of the peptide length over the affected mutated positions. Resulting peptides were filtered against the human proteome (UniProt *UP000005640*, derived: 02/29/16) and the Ensembl proteome reference (release 84, 04/27/2016) to avoid the selection of identical peptides, contained within wild-type proteins. In case of frameshift mutations, the reading frame offset was monitored in order to determine sequences of alternative reading frames, resulting in altered amino acid sequences and therefore yielding neoepitopes. Transcript information was retrieved *via BioMart*, based on the stable database version of GRCh37 (http://feb2014.archive.ensembl.org). HLA-binding prediction was performed with SYFPEITHI (26), netMHC 4.0 (27, 28), and netMHCpan 3.0 (29, 30).

The workflow was implemented using FRED2 (31). All reported predictions include variant details, mutated peptide sequence, HLA allele, prediction method, corresponding binding score, half maximal score, and a qualitative distinction between binding and non-binding peptides, which is based on the score of the corresponding method. SYFPEITHI-predicted peptides were considered binders, when prediction scores exceeded half of the maximal score of the corresponding HLA allotype matrix. According to netMHC and netMHCpan, predicted peptides with affinities (IC_{50} values in nM) ≤ 500 nM were selected. Results were further annotated with gene expression values, protein quantification values, and the results of HLA ligandome analysis.

Database matching

HLA ligandome database queries refer to the in-house database maintained at the Department of Immunology encompassing > 300,000 unique HLA class I peptides identified through MS/MS in diverse tissues (non-malignant as well as with pathologies including malignancies). Database matching was carried out using rSQL, querying for an exact string match of the respective wild-type

ligand (WT^{lig}) matching to the respective predicted neoepitope (PNE). All HLA class I allotypes of our HCC and Mel cohort were covered by respective samples in the database. Each sample containing the respective ligand was counted as a separate match.

333,431 different HLA class I peptides have been identified on benign (n=631), malignant (n=780) or (n=115) human samples with pathologies including both primary tissues and (established) cell lines. In total, the database comprises 2,646,952 HLA class I peptides corresponding to 49 different HLA alleles, encompassing 18 HLA-A, 27 HLA-B, and 14 HLA-C alleles, including duplicates.

Besides neoepitopes, we additionally screened our HCC HLA class I ligandome dataset against cancer-testis antigens (CTAs) as deposited in the CTDatabase (http://www.cta.lncc.br; (32)).

Queries against the Immune Epitope Database (IEDB; http://www.iedb.org/) were performed after filtering for HLA class I ligands, annotated as positive, positive-high, positive-intermediate, and positive-low.

Analysis of differential gene expression

Differential gene expression (DE) was done using the R package DESeq2 (33). The biomaRt package in R was used to map DE gene IDs to Entrez IDs. These Entrez IDs were then used for KEGG pathway enrichment analysis, which was performed in R using the function enrichKEGG from the package clusterProfiler. Pathways were defined to be significantly enriched when the FDR (q-value) for each pathway did not exceed 20 %. Pathway maps were created using the function pathview from the R package pathview by plotting the KEGG graphs and color the DE genes per pathway according to the direction of their log2 fold-changes in each of the 16 patients with green (down-regulated in tumor *versus* non-malignant liver) and red (up-regulated in tumor *versus* non-malignant liver).

Pathway analysis

For pathway analysis, the R package clusterProfiler was used. Resulting KEGG pathway maps in XML format were downloaded through HTTP access from KEGG and parsed using the KEGGgraph R package. Pathway maps were finally plotted using the R package pathview as native KEGG view in png format together with log2 fold-changes of DE for all tumor *versus* autologous non-malignant liver comparisons (downregulated = green, upregulated = red). It is important to note that each coloured DE gene in the pathway graph was detected to be DE in at least one of the tumor *versus* autologous non-malignant liver comparisons while in cases the gene was not DE, the log2 fold-change for that patient is still plotted.

The gene functional classification tool from *The Database for Annotation, Visualization and Integrated Discovery* (DAVID) version 6.8 was used with standard tool parameter settings (including medium classification stringency) to categorize the list of DE genes into functionally related gene groups.

TCGA analysis

To analyze publicly available data of HCC, the R package recount was used to explore and download TCGA data from the recount2 project available at (https://jhubiostatistics.shinyapps.io/recount/). Using R, a RangedSummarizedExperiment object was downloaded that contained the full liver dataset from TCGA (424 samples), divided into three major groups: primary tumor, recurrent tumor and solid tissue normal. For simplicity we treated the primary and recurrent tumor samples as one sample group (tumor) giving a set of two groups: 50 non-malignant samples and 374 tumor samples. Using the R package DESeq2 a pairwise comparison between these two groups were performed and genes were stated as DE when they had a multiple adjusted p value < 0.05 and log2 fold-change >1 or < -1.

HLA ligandomics data analysis

MS data analysis obtained from HLA-immunoprecipitates was assessed using functionality provided by tools of the open-source software library for LC/MS OpenMS 2.3 (34). Identification and post-scoring were performed using the OpenMS adapter to Comet 2016.01 rev. 3 (35) and Percolator (3.1.1) (36). HLA ligand identification was performed against a personalized version of the human reference proteome (Swiss-Prot, reviewed UP000005640), including the patient-specific mutanome. Database search was carried out without enzymatic restriction and oxidation of methionine residues as the only dynamic modification (maximal number of modifications per peptide set to 3). The digest mass range was set to 800-2,500. Precursor charge was fixed to 2-3 and the precursor mass tolerance was set to 5 ppm. In addition, a fragment bin tolerance of 1.0 Da and a fragment bin offset of 0.4 Da was set and neutral losses were included for each peptide spectrum match (PSM). A 5 % PSM FDR threshold was calculated using Percolator, based on a competitive target-decoy approach using reversed decoy sequences and merged identifications of all replicate runs if available. Peptide quantification was achieved using MapAlignerIdentification and FeatureFinderIdentification (37) with default settings. IDs of replicates were treated as internal IDs and the median intensity of consensus features was used as final quantification value. Only quantified identifications were considered to be valid hits. HLA class I annotation was performed using an *in-house* version of SYFPEITHI (26), netMHC 4.0 (27, 28), and netMHCpan 3.0 (29, 30).

Protein quantification analysis of shotgun proteomics data

Label-free protein quantification was done using MaxQuant v1.5.00 (9). Parameter groups were defined for non-malignant liver- and tumor-derived raw files, respectively. The multiplicity was set to one. Protein N-terminal acetylation as well as oxidation of methionine residues were selected as variable modifications, whereas carbamidomethylation of cysteine residues was set as fixed modification. Trypsin was selected as enzyme with specific digestion mode. Further, we specified the match type as *MatchFromAndTo* and set the number of *MaxMissedCleavages* to two. Requantification and matching between runs were enabled. As a reference, we specified the Swiss-Prot reviewed human proteome (*version UP000005640*, derived: 02/16/2016).

References.

1. Szolek A, Schubert B, Mohr C, Sturm M, Feldhahn M, Kohlbacher O. OptiType: precision HLA typing from next-generation sequencing data. Bioinformatics. 2014;30(23):3310-6.
2. Löffler MW, Chandran PA, Laske K, Schroeder C, Bonzheim I, Walzer M, et al. Personalized peptide vaccine-induced immune response associated with long-term survival of a metastatic cholangiocarcinoma patient. J Hepatol. 2016;65(4):849-55.
3. Kowalewski DJ, Stevanovic S. Biochemical large-scale identification of MHC class I ligands. Methods Mol Biol. 2013;960:145-57.
4. Barnstable CJ, Bodmer WF, Brown G, Galfre G, Milstein C, Williams AF, et al. Production of monoclonal antibodies to group A erythrocytes, HLA and other human cell surface antigens-new tools for genetic analysis. Cell. 1978;14(1):9-20.
5. Löffler MW, Kowalewski DJ, Backert L, Bernhardt J, Adam P, Schuster H, et al. Mapping the HLA Ligandome of Colorectal Cancer Reveals an Imprint of Malignant Cell Transformation. Cancer Res. 2018;78(16):4627-41.
6. MacLean B, Tomazela DM, Shulman N, Chambers M, Finney GL, Frewen B, et al. Skyline: an open source document editor for creating and analyzing targeted proteomics experiments. Bioinformatics. 2010;26(7):966-8.
7. Schilling B, Rardin MJ, MacLean BX, Zawadzka AM, Frewen BE, Cusack MP, et al. Platform-independent and label-free quantitation of proteomic data using MS1 extracted ion chromatograms in skyline: application to protein acetylation and phosphorylation. Mol Cell Proteomics. 2012;11(5):202-14.
8. Eng JK, McCormack AL, Yates JR. An approach to correlate tandem mass spectral data of peptides with amino acid sequences in a protein database. J Am Soc Mass Spectrom. 1994;5(11):976-89.
9. Cox J, Mann M. MaxQuant enables high peptide identification rates, individualized p.p.b.-range mass accuracies and proteome-wide protein quantification. Nat Biotechnol. 2008;26(12):1367-72.
10. Cox J, Neuhauser N, Michalski A, Scheltema RA, Olsen JV, Mann M. Andromeda: a peptide search engine integrated into the MaxQuant environment. J Proteome Res. 2011;10(4):1794-805.
11. Mohr C, Friedrich A, Wojnar D, Kenar E, Polatkan AC, Codrea MC, et al. qPortal: A platform for data-driven biomedical research. PLoS One. 2018;13(1):e0191603.
12. Sturm M, Schroeder C, Bauer P. SeqPurge: highly-sensitive adapter trimming for paired-end NGS data. BMC Bioinformatics. 2016;17:208.
13. Li H, Durbin R. Fast and accurate short read alignment with Burrows-Wheeler transform. Bioinformatics. 2009;25(14):1754-60.
14. Faust GG, Hall IM. SAMBLASTER: fast duplicate marking and structural variant read extraction. Bioinformatics. 2014;30(17):2503-5.
15. Mose LE, Wilkerson MD, Hayes DN, Perou CM, Parker JS. ABRA: improved coding indel detection via assembly-based realignment. Bioinformatics. 2014;30(19):2813-5.
16. Saunders CT, Wong WS, Swamy S, Becq J, Murray LJ, Cheetham RK. Strelka: accurate somatic small-variant calling from sequenced tumor-normal sample pairs. Bioinformatics. 2012;28(14):1811-7.
17. Kim S, Scheffler K, Halpern AL, Bekritsky MA, Noh E, Kallberg M, et al. Strelka2: fast and accurate calling of germline and somatic variants. Nat Methods. 2018;15(8):591-4.
18. Cingolani P, Patel VM, Coon M, Nguyen T, Land SJ, Ruden DM, et al. Using Drosophila melanogaster as a Model for Genotoxic Chemical Mutational Studies with a New Program, SnpSift. Front Genet. 2012;3:35.

19. Cingolani P, Platts A, Wang le L, Coon M, Nguyen T, Wang L, et al. A program for annotating and predicting the effects of single nucleotide polymorphisms, SnpEff: SNPs in the genome of Drosophila melanogaster strain w1118; iso-2; iso-3. Fly (Austin). 2012;6(2):80-92.
20. Liu X, Jian X, Boerwinkle E. dbNSFP: a lightweight database of human nonsynonymous SNPs and their functional predictions. Hum Mutat. 2011;32(8):894-9.
21. Dobin A, Davis CA, Schlesinger F, Drenkow J, Zaleski C, Jha S, et al. STAR: ultrafast universal RNA-seq aligner. Bioinformatics. 2013;29(1):15-21.
22. Jiang H, Lei R, Ding SW, Zhu S. Skewer: a fast and accurate adapter trimmer for next-generation sequencing paired-end reads. BMC Bioinformatics. 2014;15:182.
23. Li H, Handsaker B, Wysoker A, Fennell T, Ruan J, Homer N, et al. The Sequence Alignment/Map format and SAMtools. Bioinformatics. 2009;25(16):2078-9.
24. Kim D, Pertea G, Trapnell C, Pimentel H, Kelley R, Salzberg SL. TopHat2: accurate alignment of transcriptomes in the presence of insertions, deletions and gene fusions. Genome Biol. 2013;14(4):R36.
25. Anders S, Pyl PT, Huber W. HTSeq--a Python framework to work with high-throughput sequencing data. Bioinformatics. 2015;31(2):166-9.
26. Rammensee H, Bachmann J, Emmerich NP, Bachor OA, Stevanovic S. SYFPEITHI: database for MHC ligands and peptide motifs. Immunogenetics. 1999;50(3-4):213-9.
27. Andreatta M, Nielsen M. Gapped sequence alignment using artificial neural networks: application to the MHC class I system. Bioinformatics. 2016;32(4):511-7.
28. Nielsen M, Lundegaard C, Worning P, Lauemoller SL, Lamberth K, Buus S, et al. Reliable prediction of T-cell epitopes using neural networks with novel sequence representations. Protein Sci. 2003;12(5):1007-17.
29. Hoof I, Peters B, Sidney J, Pedersen LE, Sette A, Lund O, et al. NetMHCpan, a method for MHC class I binding prediction beyond humans. Immunogenetics. 2009;61(1):1-13.
30. Nielsen M, Andreatta M. NetMHCpan-3.0; improved prediction of binding to MHC class I molecules integrating information from multiple receptor and peptide length datasets. Genome Med. 2016;8(1):33.
31. Schubert B, Walzer M, Brachvogel HP, Szolek A, Mohr C, Kohlbacher O. FRED 2: an immunoinformatics framework for Python. Bioinformatics. 2016;32(13):2044-6.
32. Almeida LG, Sakabe NJ, deOliveira AR, Silva MC, Mundstein AS, Cohen T, et al. CTdatabase: a knowledge-base of high-throughput and curated data on cancer-testis antigens. Nucleic Acids Res. 2009;37:D816-9.
33. Love MI, Huber W, Anders S. Moderated estimation of fold change and dispersion for RNA-seq data with DESeq2. Genome Biol. 2014;15(12):550.
34. Bertsch A, Gropl C, Reinert K, Kohlbacher O. OpenMS and TOPP: open source software for LC-MS data analysis. Methods Mol Biol. 2011;696:353-67.
35. Eng JK, Jahan TA, Hoopmann MR. Comet: an open-source MS/MS sequence database search tool. Proteomics. 2013;13(1):22-4.
36. Kall L, Canterbury JD, Weston J, Noble WS, MacCoss MJ. Semi-supervised learning for peptide identification from shotgun proteomics datasets. Nat Methods. 2007;4(11):923-5.
37. Weisser H, Choudhary JS. Targeted Feature Detection for Data-Dependent Shotgun Proteomics. J Proteome Res. 2017;16(8):2964-74.
38. Chalmers ZR, Connelly CF, Fabrizio D, Gay L, Ali SM, Ennis R, et al. Analysis of 100,000 human cancer genomes reveals the landscape of tumor mutational burden. Genome Med. 2017;9(1):34.
39. Bassani-Sternberg M, Braunlein E, Klar R, Engleitner T, Sinitcyn P, Audehm S, et al. Direct identification of clinically relevant neoepitopes presented on native human melanoma tissue by mass spectrometry. Nat Commun. 2016;7:13404.
