# Supplementary material for: Multi-omics discovery of exome-derived neoantigens in hepatocellular carcinoma
Source: Genome Med. 2019 Apr 30;11:28. doi: 10.1186/s13073-019-0636-8 (PMC6492406; doi:10.1186/s13073-019-0636-8)
Supplement: Supplementary file 3 — Supplementary Figures. Figure S1. HLA ligandomics yields. Figure S2. Comparison of the tumor mutational burden in Mel and HCC. Figure S3. Differential expression heatmap. Figure S4. Principal component analysis (PCA) on principal component one level. (PDF 642 kb) [file 13073_2019_636_MOESM3_ESM.pdf]

Multi-omics discovery of exome-derived neoantigens in hepatocellular carcinoma

Markus W. Löffler[1,2,3,4,#], Christopher Mohr[5,6,#], Leon Bichmann[2,7,8], Lena Katharina Freudenmann[2,3], Mathias Walzer[2,7,8,9], Christopher M. Schroeder[10], Nico Trautwein[2], Franz J. Hilke[10], Raphael S. Zinser[2], Lena Mühlenbruch[2], Daniel J. Kowalewski[2,11], Heiko Schuster[2,11], Marc Sturm[10], Jakob Matthes[10], Olaf Riess[10, 12], Stefan Czemmel[6], Sven Nahnsen[6], Ingmar Königsrainer[1], Karolin Thiel[1], Silvio Nadalin[1], Stefan Beckert[1,13], Hans Bösmüller[14], Falko Fend[14], Ana Velic[15], Boris Maček[15], Sebastian P. Haen[2,3,16], Luigi Buonaguro[17], Oliver Kohlbacher[3,5,6,7,8,12,18], Stefan Stevanović[2,3], Alfred Königsrainer[1,3], HEPAVAC Consortium and Hans-Georg Rammensee[2,3]

[1] University Hospital Tübingen, Department of General, Visceral and Transplant Surgery, Hoppe-Seyler-Str. 3, D-72076 Tübingen, Germany
[2] University of Tübingen, Interfaculty Institute for Cell Biology, Department of Immunology, Auf der Morgenstelle 15, D-72076 Tübingen, Germany
[3] German Cancer Consortium (DKTK) and German Cancer Research Center (DKFZ) Partner Site Tübingen, Tübingen, Germany
[4] University Hospital Tübingen, Department of Clinical Pharmacology, Auf der Morgenstelle 8, D-72076 Tübingen, Germany
[5] University Hospital Tübingen, Institute for Translational Bioinformatics, Tübingen, Germany
[6] University of Tübingen, Quantitative Biology Center (QBiC), Auf der Morgenstelle 10, D-72076 Tübingen, Germany
[7] University of Tübingen, Center for Bioinformatics, Sand 14, D-72076 Tübingen, Germany
[8] Department of Computer Science, Applied Bioinformatics, Sand 14, D-72076 Tübingen, Germany
[9] Current address: European Molecular Biology Laboratory, European Bioinformatics Institute (EMBL-EBI), Wellcome Trust Genome Campus, Hinxton, Cambridgeshire, CB10 1SD, United Kingdom
[10] University Hospital Tübingen, Institute of Medical Genetics and Applied Genomics, Calwerstr. 7, D-72076 Tübingen, Germany
[11] Current address: Immatics Biotechnologies GmbH, Paul-Ehrlich-Str. 15, D-72076 Tübingen, Germany
[12] NGS Competence Center Tübingen (NCCT), University of Tübingen, Tübingen, Germany
[13] Current address: Department of General and Visceral Surgery, Schwarzwald-Baar Hospital, Klinikstr. 11, D-78052 Villingen-Schwenningen, Germany.
[14] University Hospital Tübingen, Institute of Pathology and Neuropathology, Liebermeisterstr. 8, D-72076 Tübingen, Germany
[15] University of Tübingen, Interfaculty Institute for Cell Biology, Proteome Center Tübingen (PCT), Auf der Morgenstelle 15, D-72076 Tübingen, Germany
[16] University of Tübingen, Internal Medicine, Department for Oncology, Hematology, Immunology, Rheumatology and Pulmonology, Otfried-Müller-Str. 10, D-72076 Tübingen, Germany
[17] Cancer Immunoregulation Unit, Istituto Nazionale per lo Studio e la Cura dei Tumori, "Fondazione Pascale" - IRCCS, 80131 Naples, Italy
[18] Max Planck Institute for Developmental Biology, Biomolecular Interactions, Spemannstr. 35, D-72076 Tübingen, Germany

Corresponding authors: **Markus W. Löffler, MD**, University of Tübingen, Interfaculty Institute for Cell Biology, Department of Immunology, Auf der Morgenstelle 15, D-72076 Tübingen, Germany, E-mail: **markus.loeffler@uni-tuebingen.de**; and **Christopher Mohr, MSc** (Bioinformatics), University of Tübingen, Quantitative Biology Center (QBiC), Auf der Morgenstelle 10, D-72076 Tübingen, Germany, E-mail: **christopher.mohr@uni-tuebingen.de**

Supplementary Figures

Additional File 3.

Contents

Figure S1. *HLA ligandomics yields.* 3

Figure S2. *Comparison of the tumor mutational burden (TMB) in Mel and HCC.* 4

Figure S3. *Differential expression heatmap.* 5-6

Figure S4. *Principal component analysis (PCA) on principal component one level.* 7

References. 8

Figure S1. *HLA ligandomics yields.*

Numbers of naturally presented HLA class I-eluted peptides identified by LC-MS/MS on HCC (T) and non-malignant liver tissue (N). For each patient (n=16), the total number of identified peptides (black) and the number of peptides that were additionally predicted to bind to an HLA class I allotype of the respective patient are given (grey). The purity, defined as the percentage of predicted binders among all identified peptides is denoted by blue dots. The average number of identified HLA class I ligands per HCC sample is given as dotted line.

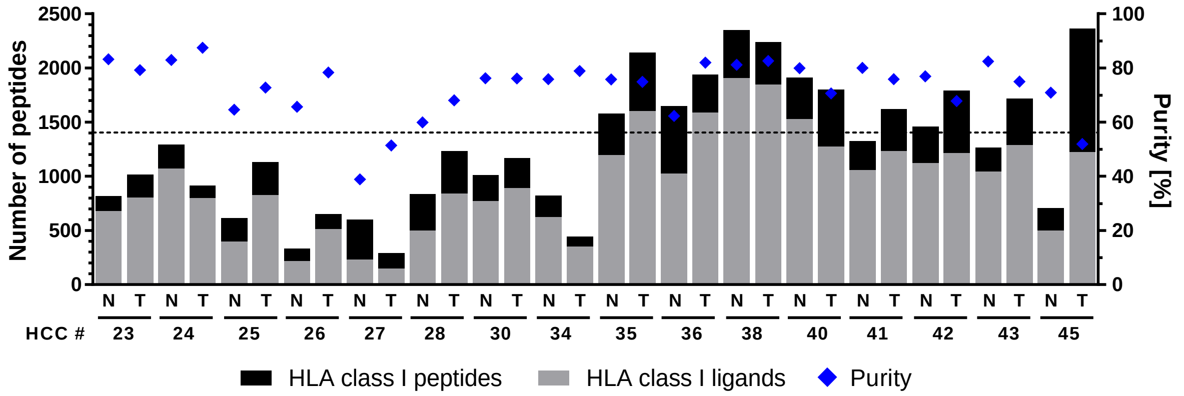

Figure S2. *Comparison of the tumor mutational burden (TMB) in Mel and HCC.*

Mutation numbers of TCGA cases (A) of Mel (n=467) and HCC (n=363) were retrieved from Genomics Data Commons Data Portal (https://portal.gdc.cancer.gov/, Access Date: 2018-09-14/ 2018-09-16). Variants were filtered for missense variants, frameshift variants, inframe deletions, inframe insertions, and coding sequence variants. Variants that were called by Mutect2 are considered. The number of Var^{ns} for each patient (n=16) in our HCC cohort (B), and (C) the Mel dataset of Bassani-Sternberg *et al. (1)* (n=5), and mean number of Var^{ns} (±SD), are illustrated below.

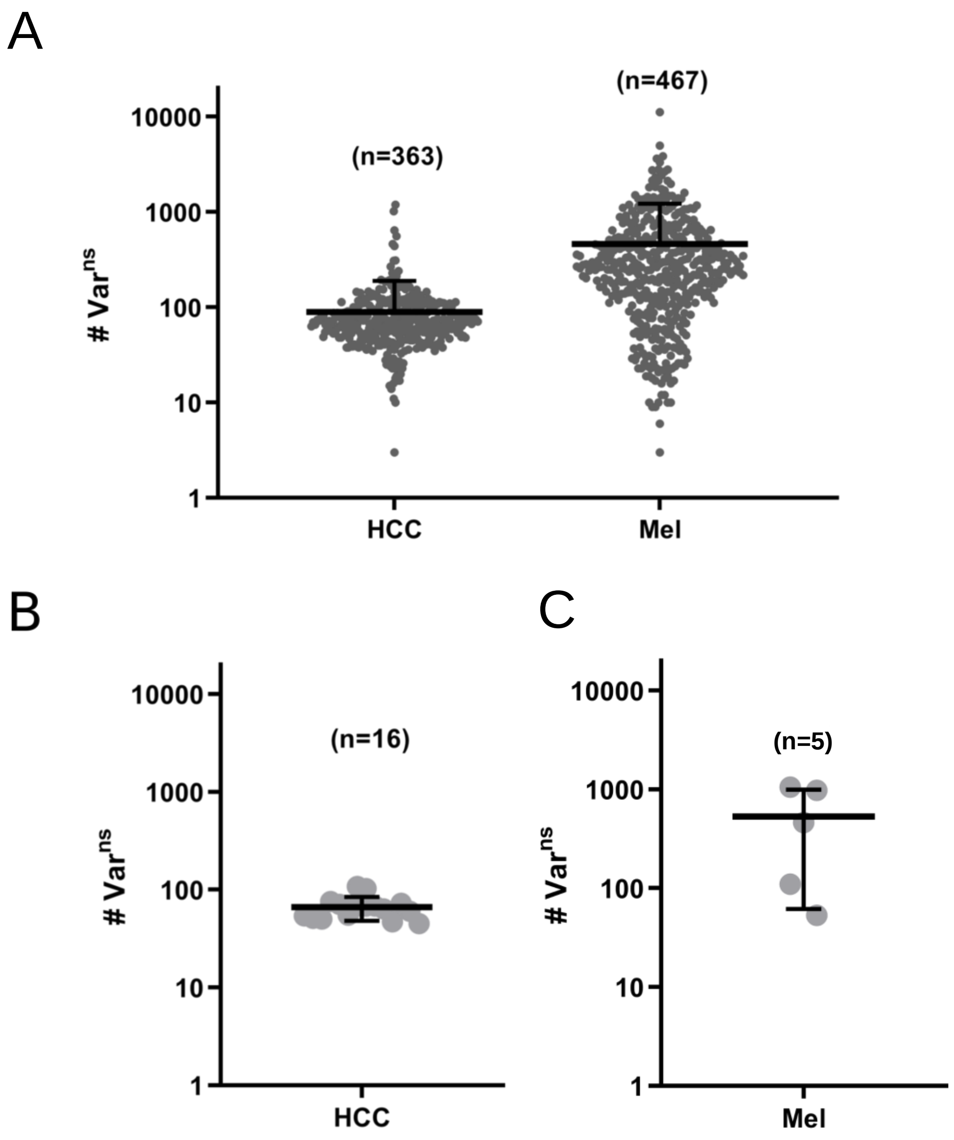

Figure S3. *Differential expression heatmap.*

Heatmap-based visualization of differentially expressed genes (n=213) found in HCC this study. Samples are depicted on the lower border of the plot and genes are depicted on the right side. Expression levels are shown as variance stabilized transformed (vst) counts (see DESeq2 documentation for details; DOI: 10.18129/B9.bioc.DESeq2). Blue and red colors represent high and low expression, respectively. Dendrograms for rows and columns are based on Euclidean distance as distance measure and complete linkage as hierarchical clustering method.

(please note the following page)

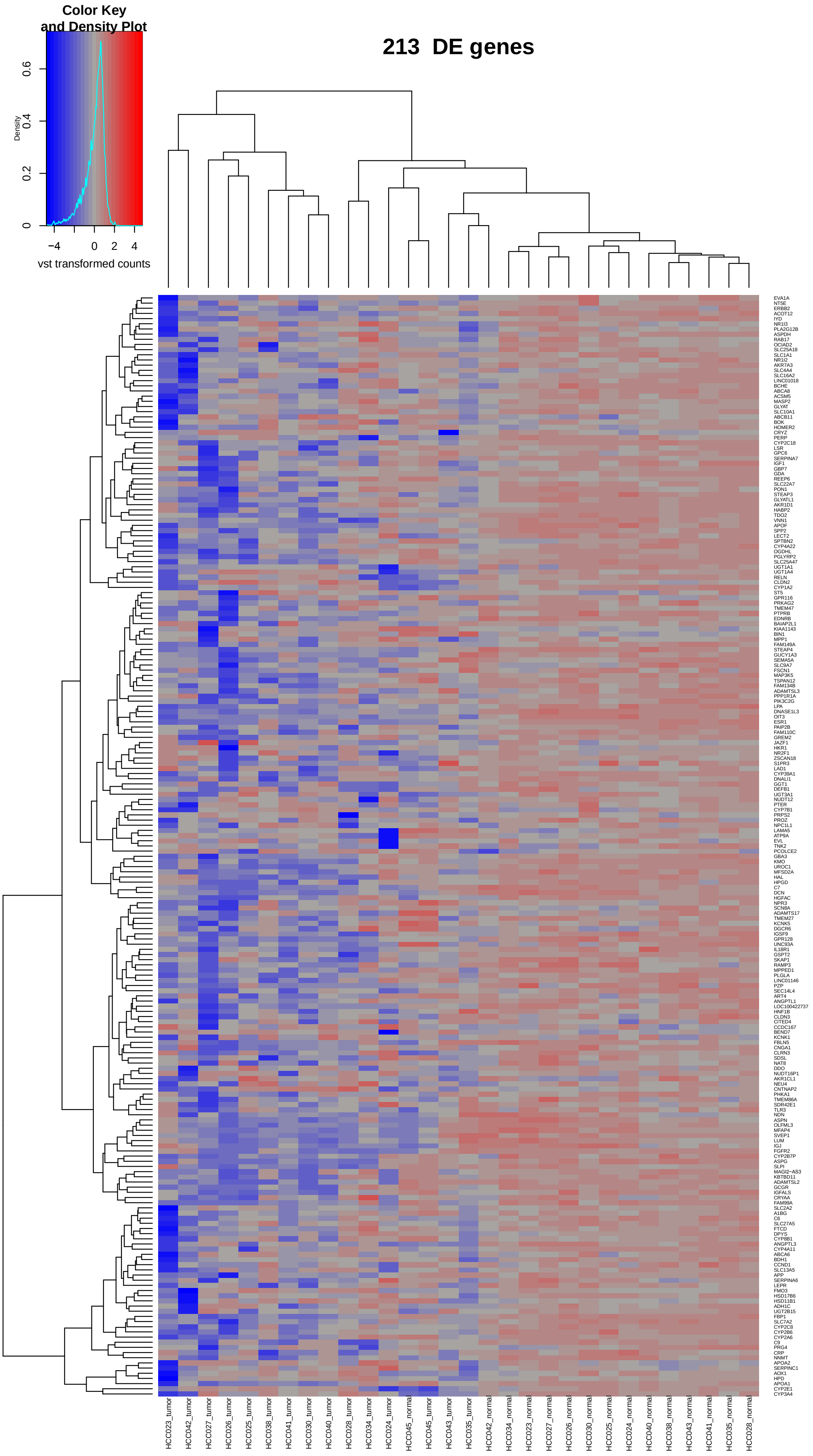
Color Key
and Density Plot
Density
0.6
0.4
0.2
0
−4
0
2
4
vst transformed counts
213 DE genes
HCC023_tumor
HCC042_tumor
HCC027_tumor
HCC026_tumor
HCC025_tumor
HCC038_tumor
HCC041_tumor
HCC030_tumor
HCC040_tumor
HCC028_tumor
HCC034_tumor
HCC024_tumor
HCC045_normal
HCC045_tumor
HCC043_tumor
HCC035_tumor
HCC042_normal
HCC034_normal
HCC023_normal
HCC027_normal
HCC026_normal
HCC030_normal
HCC025_normal
HCC024_normal
HCC040_normal
HCC038_normal
HCC043_normal
HCC041_normal
HCC035_normal
HCC028_normal

Figure S4. *Principal component analysis (PCA) on principal component one level.*

Principal component analysis (PCA) of regularized log (rlog) transformed RNA-Seq data for non-malignant (red) and tumor (blue) samples per HCC patient. The plot shows the first two principal components (PC1 and PC2) that account for 29% and 16% of the total variation of the data.

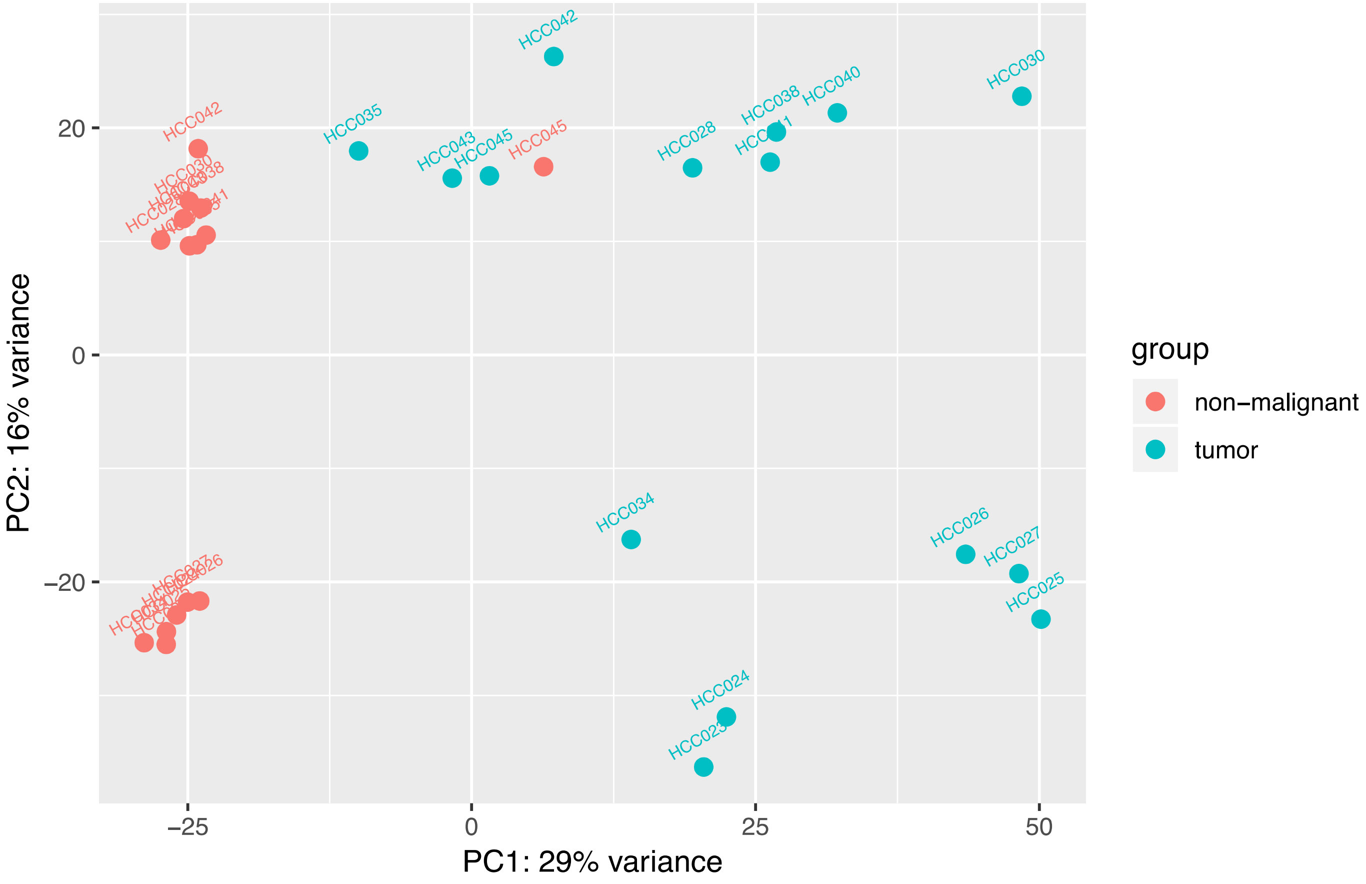

References.

1. Bassani-Sternberg M*, et al.* (2016) Direct identification of clinically relevant neoepitopes presented on native human melanoma tissue by mass spectrometry. *Nat Commun* 7:13404.
